# Global Analysis and Comparison of the Transcriptomes and Proteomes of Group A *Streptococcus* Biofilms

**DOI:** 10.1128/mSystems.00149-16

**Published:** 2016-12-06

**Authors:** Jeffrey A. Freiberg, Yoann Le Breton, Bao Q. Tran, Alison J. Scott, Janette M. Harro, Robert K. Ernst, Young Ah Goo, Emmanuel F. Mongodin, David R. Goodlett, Kevin S. McIver, Mark E. Shirtliff

**Affiliations:** a Department of Microbial Pathogenesis, University of Maryland School of Dentistry, Baltimore, Maryland, USA; b Department of Cell Biology & Molecular Genetics and Maryland Pathogen Research Institute, University of Maryland—College Park, College Park, Maryland, USA; c Department of Pharmaceutical Sciences, University of Maryland School of Pharmacy, Baltimore, Maryland, USA; d Department of Microbiology and Immunology, University of Maryland School of Medicine, Baltimore, Maryland, USA; e Institute for Genome Sciences, University of Maryland School of Medicine, Baltimore, Maryland, USA; University of California San Diego

**Keywords:** LC-MS/MS, RNA-seq, shotgun proteomics, *Streptococcus pyogenes*, transcriptomics

## Abstract

To gain a better understanding of the genes and proteins involved in group A *Streptococcus* (GAS; *Streptococcus pyogenes*) biofilm growth, we analyzed the transcriptome, cellular proteome, and cell wall proteome from biofilms at different stages and compared them to those of plankton-stage GAS. Using high-throughput RNA sequencing (RNA-seq) and liquid chromatography-tandem mass spectrometry (LC-MS/MS) shotgun proteomics, we found distinct expression profiles in the transcriptome and proteome. A total of 46 genes and 41 proteins showed expression across the majority of biofilm time points that was consistently higher or consistently lower than that seen across the majority of planktonic time points. However, there was little overlap between the genes and proteins on these two lists. In line with other studies comparing transcriptomic and proteomic data, the overall correlation between the two data sets was modest. Furthermore, correlation was poorest for biofilm samples. This suggests a high degree of regulation of protein expression by nontranscriptional mechanisms. This report illustrates the benefits and weaknesses of two different approaches to global expression profiling, and it also demonstrates the advantage of using proteomics in conjunction with transcriptomics to gain a more complete picture of global expression within biofilms. In addition, this report provides the fullest characterization of expression patterns in GAS biofilms currently available.

**IMPORTANCE** Prokaryotes are thought to regulate their proteomes largely at the level of transcription. However, the results from this first set of global transcriptomic and proteomic analyses of paired microbial samples presented here show that this assumption is false for the majority of genes and their products in *S. pyogenes*. In addition, the tenuousness of the link between transcription and translation becomes even more pronounced when microbes exist in a biofilm or a stationary planktonic state. Since the transcriptome level does not usually equal the proteome level, the validity attributed to gene expression studies as well as proteomic studies in microbial analyses must be brought into question. Therefore, the results attained by either approach, whether RNA-seq or shotgun proteomics, must be taken in context and evaluated with particular care since they are by no means interchangeable.

## Abstract (precis)

Prokaryotes are thought to regulate their proteomes largely at the level of transcription. However, the results from this first set of global transcriptomic and proteomic analyses of paired microbial samples presented here show that this assumption is false for the majority of genes and their products in *S. pyogenes*. In addition, the tenuousness of the link between transcription and translation becomes even more pronounced when microbes exist in a biofilm or a stationary planktonic state. Since the transcriptome level does not usually equal the proteome level, the validity attributed to gene expression studies as well as proteomic studies in microbial analyses must be brought into question. Therefore, the results attained by either approach, whether RNA-seq or shotgun proteomics, must be taken in context and evaluated with particular care since they are by no means interchangeable.

## INTRODUCTION

The human pathogen *Streptococcus pyogenes* (group A *Streptococcus* [GAS]) is a major cause of morbidity and mortality worldwide. In addition to asymptomatic pharyngeal carriage, GAS can cause a wide variety of different health conditions. These range from simple, superficial infections such as pharyngitis or impetigo to severe life-threatening infections such as necrotizing fasciitis or streptococcal toxic shock syndrome. The breadth of diseases that GAS can cause is due, in part, to its ability to differentially regulate expression of its genome depending on the local environment and the conditions that it encounters. One mechanism by which GAS can adapt to different environments is that of forming a biofilm. Biofilms are defined as sessile, microbially derived communities where cells secrete extracellular matrix while growing either attached to a surface or as a floating microbial conglomerate. Biofilms represent an altered growth phenotype with gene expression and protein production that differ from those seen with planktonic growth (1). GAS has been shown to form biofilms *in vivo* in several different types of infections both in animal models and in clinical samples (2–9).

Despite this strong evidence for the involvement of the biofilm phenotype during GAS infections, very little is known about the genes and proteins involved in GAS biofilm growth. A handful of studies have examined genes involved in biofilm formation and growth in GAS using targeted approaches (4, 5, 8, 10–20). While these studies found multiple genes that appear to play a role in GAS biofilms, most of the genes chosen for analysis were those encoding virulence factors or transcriptional regulators that were already well studied but only for their roles during planktonic growth. There has only been one study to date that used a global approach to measure gene expression in GAS biofilms. Cho and Caparon (3) used microarrays to compare the levels of global RNA expression of GAS biofilms to the levels of both exponential-phase and stationary-phase planktonic growth in an M14 strain. Although they identified a number of genes as being differentially regulated, they compared planktonic growth to biofilm growth at only a single time point. Furthermore, no global characterization of protein expression in GAS biofilms has ever, to our knowledge, been attempted.

In this study, we characterized and compared expression levels for both the transcriptome and the proteome of GAS biofilms at multiple stages of growth. Using a combination of high-throughput RNA sequencing (RNA-seq) and liquid chromatography-tandem mass spectrometry (LC-MS/MS) shotgun proteomics, we identified genes and proteins that are differentially regulated between the planktonic and biofilm growth stages. We were also able to identify differences in the biofilm and planktonic expression patterns of GAS virulence factors. This comprehensive *in vitro* characterization of GAS biofilms will be useful to better understand the role that GAS biofilms play in different types of *S. pyogenes* infections.

## RESULTS

### Transcriptomic analysis of GAS biofilms.

**FIG 1:**
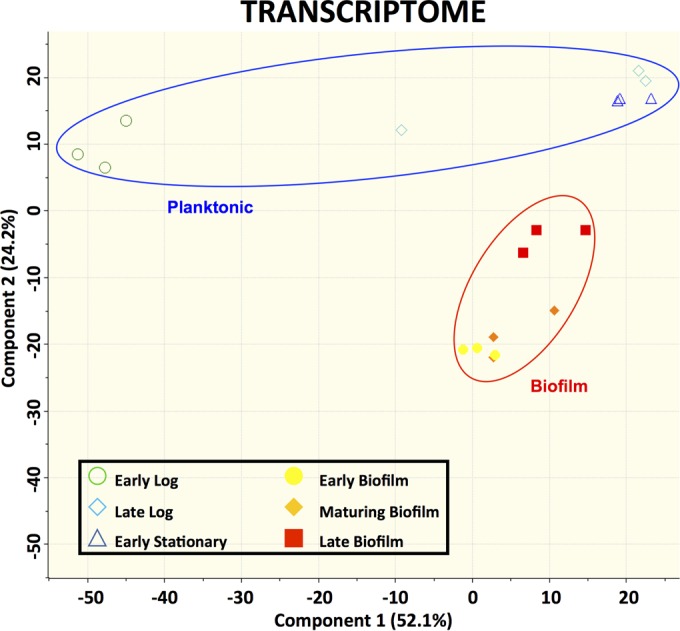
Clustering of biofilm and planktonic samples based on transcriptomic data. Data represent results of principal-component analysis (PCA) of log_2_ FPKM expression values from each sample. The PCA plot represents 1,781 genes for which expression values were available. Biological triplicates are shown by matching symbols.

**FIG 2:**
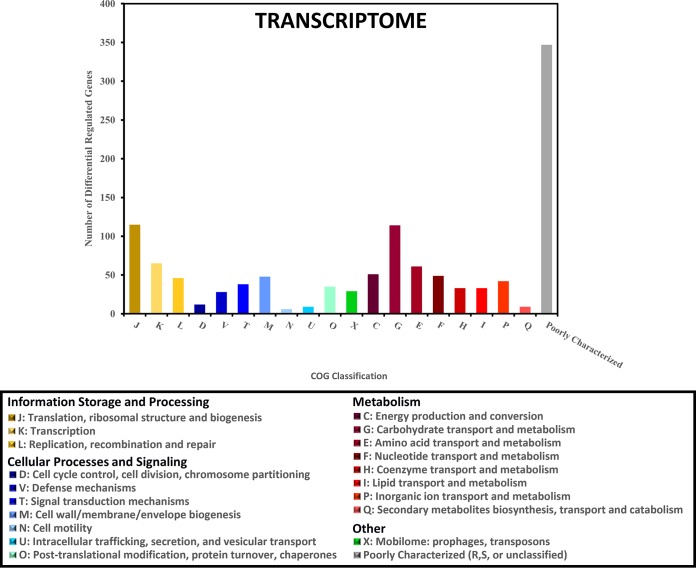
Characterization of differentially expressed genes based on their COG classification. Genes that were determined to have a significant 2-fold difference in expression between at least one biofilm time point and one planktonic time point were categorized based on their COG classification. The numbers of genes in each COG classification are shown for the 1,039 genes with differential expression based on transcriptome data. Letter designations refer to the standard COG abbreviations. Numbers sum to greater than 1,039 due to some genes fitting in two or more COG classifications. The “Poorly Characterized” group includes COG classifications R (general function prediction only) and S (unknown function) in addition to unclassified genes.

RNA extracted from GAS biofilms grown in a continuous flow reactor was sequenced and compared to RNA extracted from planktonic GAS cultures. Principal-component analysis of the data obtained from RNA sequencing revealed that the transcriptomes of the biofilm and planktonic samples at various time points assembled separately from each other into distinct, isolated clusters on principal component 2 (PC2) (Fig. 1). Further analysis of the transcriptomes revealed a large number of genes with differential expression between biofilm and planktonic cultures. There were 1,039 genes, representing approximately 58% of the *S. pyogenes* genome, that showed a significant difference (false-discovery rate [FDR or *q*] < 0.01; log_2_-fold change > 1 or <−1) between at least one biofilm time point and one planktonic time point. The functional breakdown of these 1,039 genes by their assigned Cluster of Orthologous Groups (COG) classification is shown in Fig. 2. Because the 6-day biofilm and 10-day biofilm transcriptomes were nearly identical, with only two genes showing significant differences in expression, only the 10-day (late stage) biofilm was used for further determining significant differences between biofilm growth and planktonic growth. To determine whether any particular COG was overrepresented in our data, the differentially expressed genes at each of the nine biofilm-planktonic time point comparisons were analyzed using the R-package for Bacterium and virus analysis of Orthologous Groups (BOG) (21). BOG analysis revealed that the lists of differentially expressed genes for eight of the nine comparisons were significantly enriched with genes involved in carbohydrate transport and metabolism (COG cluster G) (see Fig. S1 in the supplemental material). No other COG was significantly overrepresented for more than one of the nine comparisons.

10.1128/mSystems.00149-16.2 Figure S1  Differential regulation of biofilm versus planktonic transcriptome according to COG classifications. The numbers of genes in each COG that were differentially regulated at each biofilm versus planktonic time point are shown. Dark bars indicate the numbers of genes in the COG that were upregulated, and light bars indicate the numbers of genes downregulated. COGs were analyzed with the *R*-package BOG (21) to identify COGs with a statistically greater than expected number of genes showing differential expression. *, the adjusted *P* value is <0.05 according to the Mann-Whitney rank sum test. The “Poorly Characterized” group includes COG classifications R (general function prediction only) and S (unknown function) in addition to unclassified genes. Download Figure S1, PDF file, 0.4 MB. Copyright © 2016 Freiberg et al. 2016 Freiberg et al. This content is distributed under the terms of the Creative Commons Attribution 4.0 International license.

To determine which genes were consistently up- or downregulated during biofilm growth, we restricted the list of 1,039 genes to only those that showed a significant difference for more than 75% of the biofilm versus planktonic time point comparisons. This restriction generated a list of 38 genes with consistently higher expression during biofilm growth and eight genes with significantly lower expression during biofilm growth compared to planktonic growth (Table 1). These genes are predicted to make up a total of 35 operons, suggesting that only a small handful of transcripts are consistently up- or downregulated over time during biofilm growth. Among the consistently downregulated transcripts, the majority were involved in carbohydrate transport and metabolism (G).

**TABLE 1:** Genes with consistent differential expression in the GAS transcriptome at biofilm versus planktonic time points

M5005 locus^a^	Gene	Log fold change^b^,^c^									Gene product description^d^	COG cluster(s)^e^	Operon structure^f^
		Early biofilm	Maturing biofilm	Late biofilm	Early biofilm	Maturing biofilm	Late biofilm	Early biofilm	Maturing biofilm	Late biofilm			
		Versus early log phase			Versus late log phase			Versus early stationary phase					
Upregulated													
Spy0076	*rpmJ*	1.98	1.63	1.56	1.45			1.67	1.33	1.25	50S ribosomal protein L36	I	
Spy0446		1.55	1.07	1.37	1.81	1.33	1.63	1.24		1.06	Serine kinase; regulates carbohydrate metabolism	T	A
Spy0447		1.87	1.7	1.43	1.96	1.79	1.52	1.59	1.42	1.15	Glycosyltransferase involved in cell wall biogenesis	M	A
Spy0494		1.75	1.6		3.02	2.87	1.8	3.25	3.1	2.03	Hypothetical protein		
Spy0652		1.4	1.38		2.17	2.14	1.46	1.82	1.79	1.11	Predicted flavin-nucleotide-binding protein	R	
Spy0653	*czcD*	3.04	2.94	1.82	3.18	3.08	1.96	2.53	2.43	1.31	Cobalt-zinc-cadmium resistance protein	P	
Spy0716		3.93	3.98	2.6	2.5	2.55	1.17	2.15	2.19	0.82	Hypothetical protein		
Spy0787		1.02	0.95	1.05	1.11	1.04	1.15	1.18	1.11	1.21	Fe-S-cluster-containing protein	R	
Spy0806	*srtA*	5.42	4.21	2.97	4.39	3.19	1.95	4.07	2.86	1.62	Lantibiotic streptin precursor		
Spy0807	*srtT*	4.07	3.66	3.93	3.06	2.65	2.92	2.48	2.07	2.34	Subtilin transport ATP-binding protein	V	B
Spy0808	*srtF*	3.99	3.54	4.36	2.41	1.96	2.78	1.95	1.5	2.32	Lantibiotic transport ATP-binding protein	V	B
Spy0809	*srtE*	3.6	3.35	3.32	2.19	1.94	1.91	2.57	2.32	2.29	Lantibiotic transport permease protein		B
Spy0810	*srtG*	3.52	3.54	4.01	2.06	2.07	2.54	1.95	1.96	2.43	Lantibiotic transport permease protein		B
Spy0812		2.43	2.13	2.49	2.12	1.82	2.18	1.63	1.34	1.69	Hypothetical protein		
Spy0921		2.72	2.39	2.24	2.14	1.82	1.67	1.82	1.5	1.35	ABC transporter ATP-binding protein	R	
Spy0922	*pdxK*	3.14	2.68	2.33	2.67	2.22	1.86	1.93	1.47	1.12	Putative membrane-spanning protein	S	C
Spy0923		3.17	2.89	2.22	2.37	2.09	1.42	1.9	1.63	0.95	Putative pyridoxal kinase	H	C
Spy0924		4.12	3.64	3.18	2.16	1.68	1.22	1.96	1.48	1.02	Predicted transcriptional regulator of pyridoxine metabolism	K, E	
Spy0948	*ciaR*	3.51	3.45	3.42	2.28	2.22	2.19	1.26	1.2	1.17	Two-component system DNA-binding response regulator	T, K	
Spy1147	*comEC*	1.57	1.24	1.5	1.56	1.23	1.48	1.12		1.05	Late competence protein ComEC; DNA transport	R	
Spy1168		2.75	2.3	2.87	1.86	1.41	1.98	1.56	1.11	1.68	Phage protein		
Spy1265		1.06	0.95	1.65	1.63	1.51	2.21	1.23	1.11	1.81	Ribose operon repressor	K	
Spy1282	*msrA*	2.97	3.2	2.78	1.85	2.07	1.66	1.45	1.68	1.26	Peptide methionine sulfoxide reductase MsrA/MsrB	O	D
Spy1283	*tlpA*	2.53	2.77	2.7	1.64	1.88	1.81	0.97	1.2	1.14	Thiol-disulfide isomerase or thioredoxin	O	D
Spy1284	*ccdA*	2.18	2.5	1.88	1.58	1.9	1.29	1.05	1.38		Cytochrome *c*-type biogenesis protein	C, O	D
Spy1374		1.42	1.94	1.69	1.16	1.69	1.43	0.96	1.49	1.23	Hypothetical protein		
Spy1720	*mga*	0.8	1.08	1.08	1.87	2.15	2.15	1.61	1.89	1.88	M protein *trans*-acting positive regulator		
Spy1721		1.34	1.4	2.03	2.09	2.14	2.78	1.6	1.66	2.3	Uncharacterized *mga*-associated protein		
Spy1722		0.98	1.3	2.19	0.99	1.31	2.2	1.19	1.51	2.4	Uncharacterized *mga*-associated protein		E
Spy1723	*isp*	2.55	3.16	3.29	2.26	2.88	3	1.45	2.06	2.18	Immunogenic secreted protein	M	E
Spy1724	*ihk*	1.76	1.92	2.91	1.52	1.67	2.66	1.08	1.23	2.22	Two-component system histidine kinase	T	F
Spy1725	*irr*	2.97	3.12	3.27	2.56	2.72	2.86	2.21	2.36	2.51	Two-component system response regulator	T, K	F
Spy1726		2.25	2.48	2.74	1.93	2.16	2.42	1.7	1.93	2.19	ABC-type antimicrobial peptide transport system	V	F
Spy1727		2.61	2.55	3.63	2.49	2.42	3.51	1.65		2.67	ABC-type lipoprotein export system, ATPase component	M	F
Spy1728		2.42	3.11	3.93	2.12	2.81	3.63	1.27	1.96	2.78	Multidrug efflux pump subunit	M, V	F
Spy1729		3.48	3.66	3.76	3.11	3.29	3.39	1.7	1.88	1.98	Hypothetical protein		
Spy1798	*spxA*	2.09	1.72	1.7	2.35	1.99	1.97	1.96	1.59	1.57	Transcriptional regulator	P	
Spy1815	*rpmF*	1.57	1.97	0.64	2.61	3.01	1.67	2.39	2.78	1.45	50S ribosomal protein L32	J	
Spy1843		1.54	1.64	1.5	2.2	2.3	2.16	1.3	1.4	1.26	Soluble lytic murein transglycosylase	M	
													
Downregulated													
Spy0233	*plr*	−1.61	−1.53	−1.46	−1.9	−1.81	−1.74	−1.32	−1.24	−1.16	Glyceraldehyde-3-phosphate dehydrogenase (GAPDH)	G	
Spy1384	*glyS*	−2.17	−2.13	−1.46	−2.16	−2.11	−1.44	−1.68	−1.64	−0.97	Glycine-tRNA ligase beta subunit	J	
Spy1481	*manN*	−2.44	−2.42	−1.97	−2.03	−2	−1.56	−1.05	−1.03		PTS, mannose-specific IID component	G	
Spy1666	*rpsO*	−3.34	−3.27	−2.17	−2.2	−2.13	−1.03	−2.01	−1.94	−0.85	30S ribosomal protein S15	J	
Spy1679	*pulA*	−1.62	−1.18	−0.93	−2.23	−1.79	−1.54	−2.47	−2.03	−1.79	Pullulanase/glycogen debranching enzyme	G	
Spy1681	*dexB*	−1.2	−0.9	−1.12	−2.96	−2.67	−2.88	−2.77	−2.47	−2.69	Dextran glucosidase	G	
Spy1682	*msmK*	−0.99	−1.18	−1.24	−2.3	−2.49	−2.56	−1.77	−1.96	−2.02	Multiple sugar transport ATP-binding protein	G	
Spy1683	*lrp*	−1.59	−1.73	−1.44	−1.38	−1.51	−1.22	−1.21	−1.34	−1.05	Leucine-rich protein	K	

a “Upregulated” and “Downregulated” refer to genes upregulated and downregulated in >75% of biofilm versus planktonic time point comparisons, respectively.

b Numbers represent log_2_ fold change between the biofilm and planktonic time points.

c Missing numbers indicate that differential expression data were not statistically significant for the given comparison.

d Gene descriptions derived from the NCBI and/or Uniprot database. PTS, phosphotransferase system.

e Letters refer to the functional categories of the assigned Cluster of Orthologous Group (COG) (http://www.ncbi.nlm.nih.gov/COG).

f Identical letters indicate genes predicted to be in the same operon according to analysis by the program Rockhopper.

In addition to the 46 genes that were consistently up- or downregulated in biofilm samples, another group of 48 genes spread across 27 operons showed significant differences in gene expression between the majority of biofilm and planktonic time points (Table 2). These 48 genes were all more highly expressed at every biofilm time point compared to early-log-phase planktonic cultures. However, these same genes all showed even greater expression in the late log and stationary phases of planktonic growth than at all biofilm time points. As with many other genes showing differential expression between biofilm and planktonic growth, the majority of these 48 genes were involved in carbohydrate transport and metabolism.

**TABLE 2:** Genes upregulated during biofilm growth versus early log phase but downregulated during biofilm growth versus late log and stationary phases

M5005 locus^a^	Gene	Log fold change^b^,^c^									Putative gene product or function^d^	COG cluster(s)^e^	Operon structure^f^
		Early biofilm	Maturing biofilm	Late biofilm	Early biofilm	Maturing biofilm	Late biofilm	Early biofilm	Maturing biofilm	Late biofilm			
		Versus early log phase			Versus late log phase			Versus early stationary phase					
Spy0040	*adhA*	1.93	2.02	2.2	−2.73	−2.64	−2.46	−1.86	−1.77	−1.59	Alcohol dehydrogenase	R	
Spy0118		1.4	1.33		−1.03	−1.09	−1.45	−1.47	−1.53	−1.89	LysR family transcriptional regulator	K	
Spy0151	*ulaD*			1.69	−2.57	−2.46	−2.02	−1.88	−1.77	−1.33	3-Keto-l-gulonate-6-phosphate decarboxylase	G	A
Spy0152			1.55	1.86	−3.05	−2.89	−2.58	−2.03	−1.87	−1.56	Putative l-xylulose 5-phosphate 3-epimerase	G	A
Spy0153	*araD*			1.68	−3.89	−2.84	−2.35	−3.13	−2.08	−1.59	l-Ribulose-5-phosphate 4-epimerase	G	A
Spy0212		2.02	1.85	1.61	−1.96	−2.13	−2.38	−1.7	−1.86	−2.11	N-Acetylmannosamine-6-phosphate 2-epimerase	G	B
Spy0213		1.82	1.83	1.55	−2.07	−2.05	−2.34	−1.83	−1.82	−2.1	N-Acetylneuraminate-binding protein	G	B
Spy0214		1.08	1.08		−1.4	−1.41	−1.63	−1.34	−1.34	−1.57	N-Acetylneuraminate transport system permease	G	
Spy0340	*lctO*	5.77	5.57	5.17	−2.6	−2.8	−3.2	−2.28	−2.47	−2.88	l-Lactate oxidase	C, I	
Spy0341		4.37	4.72	4.2	−2.27	−1.93	−2.45	−2.7	−2.36	−2.88	Lactocepin	O	
Spy0534		2.31	2.18	2.8	−1.63	−1.77	−1.14	−1.74	−1.87	−1.25	Acetoin reductase	I, Q	
Spy0790	*gabD*	1.7	1.92	2.01	−1.34	−1.12	−1.04	−1.83	−1.61	−1.52	Succinate-semialdehyde dehydrogenase	C	
Spy0834		4.06	3.77	3.47	−0.85	−1.14	−1.44	−0.83	−1.12	−1.42	Zn-dependent alcohol dehydrogenase	E	
Spy0971		1.09	1.42	2.11	−1.74	−1.41	−0.72	−2.91	−2.58	−1.89	Gls24 family general stress protein	S	C
Spy0974		1.14	1.41	2.75	−2.26	−2	−0.66	−2.71	−2.45	−1.11	Small integral membrane protein	S	C
Spy0975		1.85	2.1	2.83	−1.68	−1.43		−2.22	−1.97	−1.24	Hypothetical protein		C
Spy1062	*malA*	1.65	1.69	2.63	−2.15	−2.11	−1.17	−2.53	−2.49	−1.55	Maltodextrose utilization protein	G	D
Spy1063	*malD*	2.59	2.5	2.16	−1.32	−1.41	−1.75	−1.37	−1.46	−1.79	Maltodextrin transport system permease protein	G	D
Spy1064	*malC*	3.04	2.85	2.27	−0.95	−1.13	−1.71	−1.25	−1.44	−2.01	Maltose transport system permease protein	G	D
Spy1065	*amyA*	2.62	2.52	2.79	−1.1	−1.2	−0.93	−1.73	−1.83	−1.56	Alpha-amylase	G	D
Spy1066	*amyB*	2.27	2.05	2.33	−1.51	−1.73	−1.44	−1.78	−2.01	−1.72	Cyclomaltodextrinase	G	D
Spy1067	*malX*	3	2.76	2.78	−1.32	−1.56	−1.54	−1.62	−1.85	−1.84	Maltose/maltodextrin-binding protein	G	
Spy1093		3.09	2.98	2.63	−1.84	−1.95	−2.3	−1.69	−1.81	−2.15	Hypothetical protein	R	
Spy1270	*arcC*	4.2	4.39	5.17	−3.65	−3.46	−2.68	−3.67	−3.48	−2.7	Carbamate kinase	E	E
Spy1271	*arcT*	5.42	5.42	5.7	−2.85	−2.85	−2.58	−2.66	−2.66	−2.39	Xaa-His dipeptidase	E	E
Spy1272	*arcD*	4.63	4.76	4.92	−3.53	−3.39	−3.24	−2.94	−2.8	−2.65	Arginine/ornithine antiporter	R	E
Spy1273	*arcB*	4.73	4.72	4.72	−3.6	−3.6	−3.61	−2.62	−2.63	−2.63	Ornithine carbamoyltransferase	E	F
Spy1274		4.82	4.92	6.04	−3.94	−3.84	−2.72	−3.32	−3.22	−2.1	Acetyltransferase	-	F
Spy1275	*arcA*	5.4	6.02	5.9	−3.54	−2.92	−3.05	−2.63	−2	−2.13	Arginine deiminase	E	F
Spy1314	*hyl*	1.12	1.35	1.17	−1.72	−1.5	−1.67	−1.7	−1.48	−1.65	Hyaluronoglucosaminidase	-	
Spy1316		1.04	2.03	1.66	−1.72	−0.72	−1.09	−2.11	−1.12	−1.49	Hypothetical protein	S	
Spy1376	*tal*	1.14	1.35	1.38	−1.96	−1.74	−1.71	−1.92	−1.7	−1.67	Putative translaldolase	G	
Spy1395	*lacD.1*	3.34	3.58	3.45	−1.75	−1.5	−1.63	−1.55	−1.3	−1.43	Tagatose 1,6-diphosphate aldolase	G	
Spy1396		3.37	3.8	4.18	−2.49	−2.07	−1.69	−2.4	−1.97	−1.6	Tagatose-6-phosphate kinase	-	G
Spy1397	*lacB.1*	3.21	3.48	4.08	−2.6	−2.32	−1.73	−2.22	−1.95	−1.36	Galactose-6-phosphate isomerase subunit LacB	G	G
Spy1398	*lacA.1*	3.56	3.84	4.16	−2.32	−2.04	−1.72	−2.14	−1.86	−1.54	Galactose-6-phosphate isomerase subunit LacA	G	G
Spy1399		4.39	4.46	4.47	−2.95	−2.89	−2.88	−2.51	−2.44	−2.43	PTS, galactose-specific IIC component	G	H
Spy1400		4.61	5.81	5.78	−3.16	−1.97	−2	−2.61		−1.44	PTS, galactose-specific IIB component	G	H
Spy1401		4.3	4.48	5.03	−3.34	−3.16	−2.6	−2.2	−2.02	−1.47	PTS, galactose-specific IIA component	G, T	H
Spy1632	*lacG*	1.62	2.67	2.2	−2.85	−1.81	−2.28	−2.03	−0.99	−1.46	6-Phospho-beta-galactosidase	G	I
Spy1633	*lacE*	2.69	3.99	3.08	−3.63	−2.33	−3.24	−2.52	−1.22	−2.13	PTS, lactose-specific IIBC component	G	I
Spy1634	*lacF*	3.54	4.8	4.1	−3.44	−2.17	−2.87	−2.44		−1.88	PTS, lactose-specific IIA component	G	I
Spy1635	*lacD.2*	3.99	5.45	4.37	−2.92	−1.45	−2.53	−1.99		−1.6	Tagatose 1,6-diphosphate aldolase	G	I
Spy1636	*lacC.2*	3.21	4.26	3.41	−3.19	−2.14	−3	−1.7		−1.51	Tagatose-6-phosphate kinase	G	I
Spy1637	*lacB.2*	4.1	5.17	3.54	−2.11	−1.03	−2.66	−1.05		−1.61	Galactose-6-phosphate isomerase subunit LacB	G	I
Spy1638	*lacA.2*	3.82	4.91	4.11	−2.51	−1.41	−2.21	−1.72		−1.42	Galactose-6-phosphate isomerase subunit LacA	G	I
Spy1744		1.93	2.28	2.41	−1.9	−1.56	−1.42	−1.52	−1.18	−1.04	PTS, cellobiose-specific IIC component	G	
Spy1758		2.04	2.14	2.22	−1.29	−1.19	−1.11	−1.54	−1.44	−1.36	Dipeptidase B	E	
Spy1769	*ahpF*	1.39	1.22	1.08	−0.9	−1.07	−1.21	−2.14	−2.31	−2.46	Peroxiredoxin reductase [NAD(P)H]	V	
Spy1783	*dexS*	3.44	3.28	3.87	−1.51	−1.67	−1.08	−1.87	−2.04	−1.44	Trehalose-6-phosphate hydrolase	G	J
Spy1784		3.54	3.04	3.35	−1.65	−2.15	−1.83	−1.87	−2.37	−2.05	PTS, trehalose-specific IIBC component	G	J

a Data represent genes that were upregulated in biofilm versus the early log phase and downregulated in biofilm versus the late log and stationary phases.

b Numbers represent log_2_ fold change between the biofilm and planktonic time points.

c Missing numbers indicate that differential expression data were not statistically significant for the given comparison.

d Gene descriptions derived from the NCBI and/or Uniprot database.

e Letters refer to the functional categories of the assigned Cluster of Orthologous Group (COG) (http://www.ncbi.nlm.nih.gov/COG).

f Identical letters indicate genes predicted to be in the same operon according to analysis by the program Rockhopper.

### Proteomic analysis of GAS biofilms.

**FIG 3:**
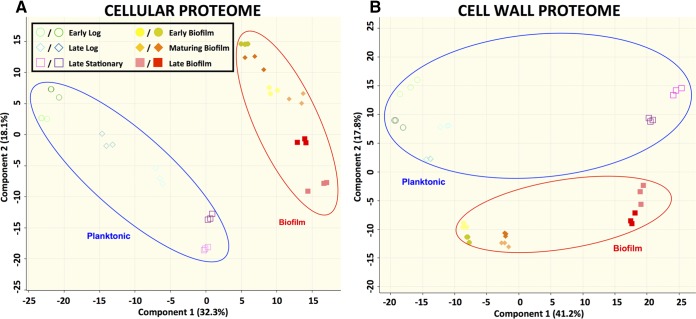
Clustering of biofilm and planktonic samples based on proteomic data. Data represent results of principal-component analysis (PCA) of log_2_ LFQ intensity values from each sample in either the cellular proteome (A) or the cell wall proteome (B). The PCA plot represents 532 proteins (cellular proteome) or 489 proteins (cell wall proteome) for which expression values were available. Technical triplicates are shown by matching symbols and colors. Biological duplicates are shown by matching symbols.

**FIG 4:**
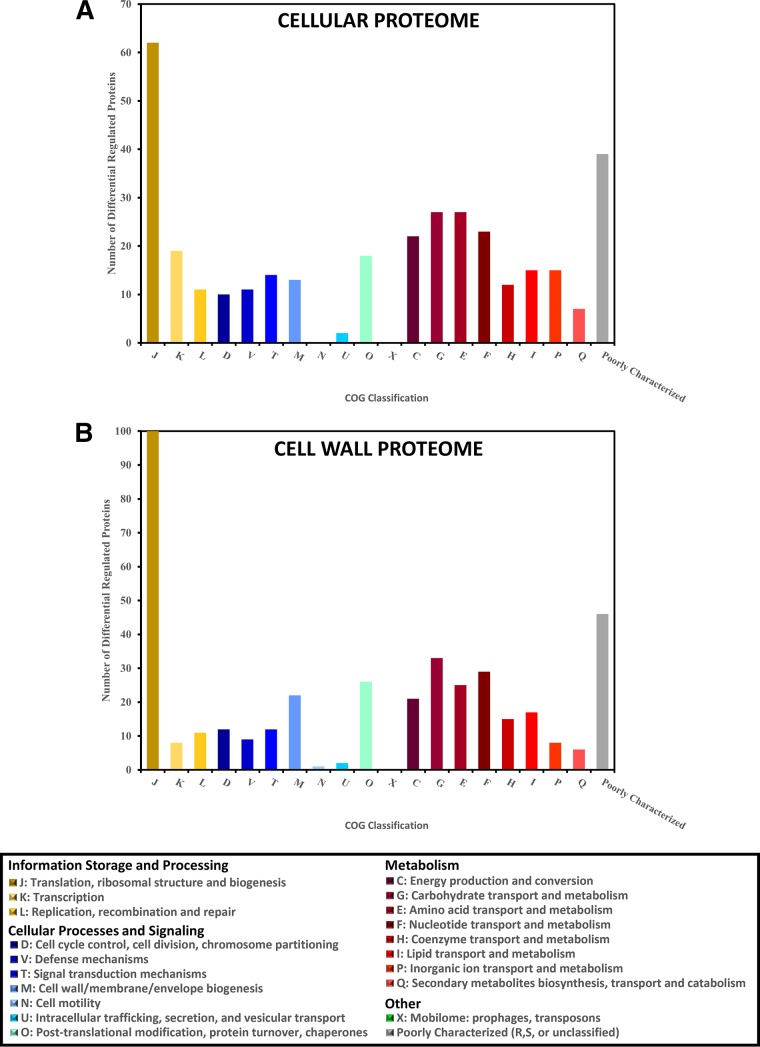
Characterization of differentially expressed proteins based on their COG classification. Proteins that were determined to have a significant 2-fold difference in expression between at least one biofilm time point and one planktonic time point were categorized based on their COG classification. The numbers of proteins in each COG classification are shown for the 320 proteins with differential expression based on cellular proteome data (A) and for the 376 proteins with differential expression based on cell wall proteome data (B). Letter designations refer to the standard COG abbreviations. Numbers sum to greater than 320 or 376 due to some proteins fitting in two or more COG classifications. The “Poorly Characterized” group includes COG classifications R (general function prediction only) and S (unknown function) in addition to unclassified proteins.

LC-MS/MS was able to identify nearly one-third of the proteins in the predicted *S. pyogenes* proteome. Similarly to what was seen with the transcriptomic data, the proteomes from the biofilm samples clustered together separately from the planktonic proteomes (Fig. 3). Between the cell wall and the cellular fractions, a total of 586 proteins were identified. The mean label-free quantification (LFQ) intensities for these 586 proteins are shown in Tables S1 and S2 in the supplemental material. Of these, only 54 proteins were identified solely in the cell wall fraction. To avoid analyzing expression differences that were unlikely to be biologically relevant, proteins with extremely low abundance (average MS/MS spectral count < 1) were excluded from further analysis. Among the remaining proteins, 467 showed a significant difference (*q* < 0.01; log_2_-fold change > 1 or <−1) between at least one biofilm time point and one planktonic time point in one of the protein fractions. Of these proteins, 147 had significant differences between biofilm and planktonic time points in the cell wall protein fraction, 91 had significant differences in the cellular protein fraction, and 229 had significant differences in both fractions. The functional breakdown of these differentially expressed proteins is shown by their assigned Cluster of Orthologous Groups (COG) classification for the cellular and cell wall fractions in Fig. 4A and B, respectively. BOG analysis revealed relatively few COGs to be significantly enriched at any of the time point comparisons (see Fig. S2 and S3). The notable exception was a significant enrichment in differentially expressed proteins involved in carbohydrate transport and metabolism in the cell wall protein fraction. Comparing all of the cell wall protein fractions from the different samples, all of the stationary-phase versus biofilm-phase time point comparisons had a greater number of differentially expressed proteins in COG cluster G than expected according to BOG analysis (see Fig. S3).

10.1128/mSystems.00149-16.8 Table S1  Mean LFQ intensities for cellular protein fraction. Download Table S1, PDF file, 0.5 MB. Copyright © 2016 Freiberg et al. 2016 Freiberg et al. This content is distributed under the terms of the Creative Commons Attribution 4.0 International license.

10.1128/mSystems.00149-16.9 Table S2  Mean LFQ intensities for cell wall protein fraction. Download Table S2, PDF file, 0.5 MB. Copyright © 2016 Freiberg et al. 2016 Freiberg et al. This content is distributed under the terms of the Creative Commons Attribution 4.0 International license.

10.1128/mSystems.00149-16.10 Table S3  Real-time RT-PCR primers used in this study. Download Table S3, PDF file, 0.1 MB. Copyright © 2016 Freiberg et al. 2016 Freiberg et al. This content is distributed under the terms of the Creative Commons Attribution 4.0 International license.

10.1128/mSystems.00149-16.3 Figure S2  Differential regulation of biofilm versus planktonic cellular proteome according to COG classifications. The numbers of cellular proteins in each COG that were differentially regulated at each biofilm versus planktonic time point are shown. Dark bars indicate the numbers of cellular proteins in the COG that were upregulated, and light bars indicate the numbers of cellular proteins downregulated. COGs were analyzed with the *R*-package BOG (21) to identify COGs with a statistically greater than expected number of cellular proteins showing differential expression. *, the adjusted *P* value is <0.05 according to the Mann-Whitney rank sum test. The “Poorly Characterized” group includes COG classifications R (general function prediction only) and S (unknown function) in addition to unclassified proteins. Download Figure S2, PDF file, 0.2 MB. Copyright © 2016 Freiberg et al. 2016 Freiberg et al. This content is distributed under the terms of the Creative Commons Attribution 4.0 International license.

10.1128/mSystems.00149-16.4 Figure S3  Differential regulation of biofilm versus planktonic cell wall proteome according to COG classifications. The numbers of cell wall proteins in each COG that were differentially regulated at each biofilm versus planktonic time point are shown. Dark bars indicate the numbers of cell wall proteins in the COG that were upregulated, and light bars indicate the numbers of cell wall proteins downregulated. COGs were analyzed with the *R*-package BOG (21) to identify COGs with a statistically greater than expected number of cell wall proteins showing differential expression. *, the adjusted *P* value is <0.05 according to the Mann-Whitney rank sum test. The “Poorly Characterized” group includes COG classifications R (general function prediction only) and S (unknown function) in addition to unclassified proteins. Download Figure S3, PDF file, 0.2 MB. Copyright © 2016 Freiberg et al. 2016 Freiberg et al. This content is distributed under the terms of the Creative Commons Attribution 4.0 International license.

Similarly to the transcriptome analysis, we restricted the list of significantly differentially expressed proteins to those that showed a significant difference for more than 75% of the biofilm versus planktonic time point comparisons. This narrowed down the 467 proteins to 41 proteins that were either consistently upregulated or consistently downregulated over time during biofilm growth. Of these 41 proteins, 8 had differential expression in only the cell wall fraction, 17 had differential expression in only the cellular fraction, and 16 had differential expression in both fractions (Tables 3 and 4). Over 80% of the differentially expressed proteins were upregulated during biofilm growth, with only 8 of the 41 proteins being consistently downregulated during biofilm growth.

**TABLE 3:** Proteins with consistent differential expression in the GAS cellular proteome at biofilm versus planktonic time points

M5005 locus^a^	Gene	Log fold change^b^,^c^									Protein description^d^	COG cluster(s)^e^
		Early biofilm	Maturing biofilm	Late biofilm	Early biofilm	Maturing biofilm	Late biofilm	Early biofilm	Maturing biofilm	Late biofilm		
		Versus early log phase			Versus late log phase			Versus late stationary phase				
Upregulated												
Spy0751	*acoA*	1.69	1.93	2.39		1.36	1.81	1.16	1.41	1.87	Pyruvate dehydrogenase E1 component alpha subunit	C
Spy0752	*acoB*	1.64	1.91	2.24	1.29	1.56	1.89	1.16	1.43	1.76	Pyruvate dehydrogenase E1 component beta subunit	C
Spy0753	*acoC*	2.03	1.99	2.92	1.55	1.51	2.45	0.89	0.85	1.78	Branched-chain alpha-keto acid dehydrogenase subunit E2	C
Spy0755	*acoL*	1.84	2.1	2.2	1.96	2.22	2.32	1.9	2.17	2.27	Dihydrolipoamide dehydrogenase	C
Spy0778	*msrB*	4.09	4.05	2.8	3.39	3.34	2.1	3.68	3.63	2.38	Methionine sulfoxide reductase B	O
Spy0781	*ptsB*	5.54	5.3	5.19	5.29	5.05	4.95	2.12	1.88	1.77	PTS mannose/fructose family IIB subunit	G
Spy0790	*gabD*	4.46	4.58	3.24	4.92	5.04	3.7	4.7	4.82	3.48	Succinate-semialdehyde dehydrogenase	C
Spy0792		1.57	1.53	1.58		2	2.05	1.56	1.51	1.57	NAD(P)H-dependent quinone reductase	C
Spy0851	*pta*	2.79	2.7	2.32	1.37	1.27	0.9	1.4	1.3	0.92	Phosphotransacetylase	C
Spy0867	*glyA*	1.18	1.49	1.41		1.54	1.46	1.78	2.1	2.02	Serine hydroxymethyltransferase	E
M1GAS476_1104^f^		3.24	2.95	2.91	2.01	1.72	1.68	2.13	1.84	1.8	Hypothetical protein	
Spy1067	*malX*	3.85	3.57	4.13	3.06	2.77	3.34	2.12	1.83	2.4	Maltose/maltodextrin-binding protein	G
Spy1235		4.87	4.96	4.4	2.43	2.52	1.96	2.46	2.55	1.99	Phosphoglucomutase	G
Spy1270	*arcC*	5.99	5.78	4.77		3.34	2.33	3.63	3.42	2.42	Carbamate kinase	E
Spy1271	*arcT*	4.74	4.87	3.67	4.48	4.61	3.41	3.66	3.79	2.59	Xaa-His dipeptidase	E
Spy1273	*arcB*	5.81	5.87	5.08	3.35	3.41	2.62	3.98	4.04	3.25	Ornithine carbamoyltransferase	E
Spy1275	*arcA*	7.5	7.29	6.64	5.39	5.18	4.53	2.97	2.76	2.11	Arginine deiminase	E
Spy1329	*cysM*	1.92	1.9	1.93	1.63	1.61	1.64	1.68	1.66	1.69	Cysteine synthase	E
Spy1356	*pepC*	0.95	0.87	1.04	1.35	1.26	1.43	2	1.91	2.08	Aminopeptidase C	E
Spy1387		2.31	2.59	3.18		1.07	1.65	0.78	1.07	1.65	Aldo/keto reductase	Q
Spy1388	*nagA*	2.03	2.01	1.18	1.86	1.85	1.02	1.75	1.74	0.91	N-Acetylglucosamine-6-phosphate deacetylase	G
Spy1400		2.92	3.08	2.54	3.59	3.75	3.2	2.05	2.21	1.66	PTS, galactose-specific IIB component	G
Spy1587	*udp*	4.14	4.05	2.56	2.35	2.26		2.39	2.3		Uridine phosphorylase	F
Spy1635	*lacD2*	3.63	3.68	2.27	3.16	3.22	1.81	2.41	2.47	1.05	Tagatose 1,6-diphosphate aldolase	G
Spy1678		3.26	3.34	2.62	1.91	1.98	1.26	1.95	2.03	1.31	Thioredoxin	O
Spy1734		6.49	6.97	5.64	3.59	4.07	2.74	3.54	4.02	2.69	Streptopain inhibitor	
Spy1768	*ahpC*	2.26	2.41	2.62	2.04	2.19	2.4	0.94	1.1	1.31	Peroxiredoxin reductase [NAD(P)H]	V
												
Downregulated												
Spy0249	*oppA*	−2.23	−2.55	−2.79	−1.96	−2.28	−2.52	−0.77	−1.09	−1.33	Oligopeptide-binding protein	E
Spy1076	*glnH*	−2.28	−2.37	−4.07		−2.1	−3.79	−1.01	−1.1	−2.79	Transporter	E, T
Spy1597		−3.21	−3.22	−3.97	−2.59	−2.6	−3.35	−1.7	−1.71	−2.46	MerR family transcriptional regulator	K
Spy1719	*emm1.0*	−6.46	−9.16	−12.93	−6.15	−8.85	−12.62	−3.03	−5.73	−9.5	M protein	D
Spy1842	*sdhA*	−1.4	−1.53	−1.72		−2.01	−2.2	−1.45	−1.59	−1.78	l-serine dehydratase	E
Spy1848		−1.69	−1.83	−1.78	−1.54	−1.68	−1.63	−1.5	−1.63	−1.58	Hypothetical protein	D

a Upregulated” and “Downregulated” refer to cellular proteins upregulated and downregulated in >75% of biofilm versus planktonic time point comparisons, respectively.

b Numbers represent log_2_ fold change between the biofilm and planktonic time points.

c Missing numbers indicate that differential expression data were not statistically significant for the given comparison.

d Gene descriptions derived from the NCBI and/or UniProt database.

e Letters refer to the functional categories of the assigned Cluster of Orthologous Group (COG) (http://www.ncbi.nlm.nih.gov/COG).

f Protein not annotated in the M5005 genome; annotation refers to *S. pyogenes* M1 strain 476.

**TABLE 4:** Proteins with consistent differential expression in the GAS cell wall proteome at biofilm versus planktonic time points

M5005 locus^a^	Protein	Log fold change^b^,^c^									Protein description^d^	COG cluster(s)^e^
		Early biofilm	Maturing biofilm	Late biofilm	Early biofilm	Maturing biofilm	Late biofilm	Early biofilm	Maturing biofilm	Late biofilm		
		Versus early log phase			Versus late log phase			Versus late stationary phase				
Upregulated												
Spy0270		2.14	2.43	3.47	1.76	2.05	3.09	−0.23		1.1	Cysteine ABC transporter, substrate-binding protein	E, T
Spy0627	Gor	1.98	2.34	3.72	1.27	1.62	3.01	−0.74		1	Glutathione reductase	C
Spy0751	acoA	1.49	1.44		1.11	1.06		3.68	3.63	2.68	Pyruvate dehydrogenase E1 component alpha subunit	C
Spy0752	acoB	1.84	1.62		1.34	1.12		3.24	3.02	2.07	Pyruvate dehydrogenase E1 component beta subunit	C
Spy0753	acoC	1.71	1.63	1.12	1.34	1.25	0.74	3.69	3.61	3.09	Branched-chain alpha-keto acid dehydrogenase subunit E2	C
Spy0755	acoL	1.61	1.56	2.61	1.31	1.26	2.31	1.83	1.78	2.83	Dihydrolipoamide dehydrogenase	C
Spy0781	ptsB	6.42	5.93	4.43	5.97	5.48	3.98	4.15	3.66	2.16	PTS mannose/fructose family IIB subunit	G
Spy0790	gabD	4.92	5.41	7.13	4.45	4.94	6.66	0.57	1.05	2.77	Succinate-semialdehyde dehydrogenase	C
Spy0792		1.51	1.35		3.04	2.87	1.58	5.41	5.24	3.95	NAD(P)H-dependent quinone reductase	C
Spy1067	malX	5.16	5.68	6.06	2.48	2.99	3.37		0.68	1.06	Maltose/maltodextrin-binding protein	G
Spy1145	sodA	2.4	2.53	3.57	1.05	1.19	2.23			1.32	Manganese superoxide dismutase	P
Spy1235		3.12	3.06	1.81	1.79	1.74		5.94	5.89	4.63	Phosphoglucomutase	G
Spy1270	arcC	5.41	6.21	7.3	3.51	4.3	5.4			1.82	Carbamate kinase	E
Spy1271	arcT	4.11	4.75	3.95	4.57	5.21	4.41	2.68	3.32	2.52	Xaa-His dipeptidase	E
Spy1273	arcB	7.1	7.77	9.04	2.8	3.46	4.74		0.65	1.92	Ornithine carbamoyltransferase	E
Spy1275	arcA	6.47	7.07	8.24	2.72	3.33	4.49		1.06	2.23	Arginine deiminase	E
Spy1376	tal	3.11	3.48	4.56	3.17	3.54	4.62			1.49	Putative translaldolase	G
Spy1400		1.98	2.14	1.72	1.98	2.14	1.72	1.76	1.92		PTS, galactose-specific IIB component	G
Spy1732	prsA2	1.87	2.41	1.12	0.99	1.54		2.73	3.27	1.98	Peptidylproline *cis*-trans-isomerase	O
Spy1734		3.75	4.29	4.31	3.03	3.57	3.59	1.65	2.19	2.21	Streptopain inhibitor	
Spy1769	ahpF	1.54	1.6		1.49	1.55		2.85	2.91	1.42	Peroxiredoxin reductase [NAD(P)H]	V
												
Downregulated												
Spy1709		−1.66	−1.64	−2.59			−1.42	−2.84	−2.82	−3.77	Hypothetical protein	S
Spy1714		−3.43	−3.68	−3.13	−3.33	−3.58	−3.03	−3.07	−3.32	−2.77	Fibronectin-binding protein	D
Spy1719	emm1.0	−3.05	−4.26	−5.72	−3.45	−4.66	−6.12	−3.3	−4.51	−5.98	M protein	D

a “Upregulated” and “Downregulated” refer to cell wall proteins upregulated and downregulated in >75% of biofilm versus planktonic time point comparisons, respectively.

b Numbers represent log_2_ fold change between the biofilm and planktonic time points.

c Missing numbers indicate that differential expression data were not statistically significant for the given comparison.

d Gene descriptions derived from the NCBI and/or UniProt database.

e Letters refer to the functional categories of the assigned Cluster of Orthologous Group (COG) (http://www.ncbi.nlm.nih.gov/COG).

### Correlation between transcriptome and proteome.

Despite both the biofilm transcriptome analysis and the biofilm proteome analysis revealing differential expression of a large number of genes or proteins involved in carbohydrate transport and metabolism, the overlap between the individual genes and proteins that were identified by each method was modest. Since we were able to identify and obtain quantitative data for only approximately one-third of the proteins in the predicted *S. pyogenes* proteome, our comparison between transcriptomic and proteomic data was limited to the genes for which corresponding proteins were identified by LC-MS/MS. Of the 46 genes found to be consistently up- or downregulated in the biofilm transcriptome (Table 1), only nine of them had a corresponding identified protein product in either the cellular or cell wall protein fractions. None of the corresponding proteins were among the proteins consistently up- or downregulated in the biofilm proteome (Tables 3 and 4). However, seven of the nine corresponding proteins show a trend in their expression that matched the regulation pattern of the corresponding transcript, despite not meeting the criteria for inclusion in Table 3 or 4 (data not shown).

Interestingly, there was a strong relationship between the 48 genes with the distinct pattern of transcript expression shown in Table 2 and the proteins that were consistently upregulated. Of the 27 operons represented in Table 2, 13 had corresponding protein data for at least one protein encoded by the operon. Of those operons with both transcriptomic and proteomic data, 85% (11 of 13) showed significantly greater protein expression for a majority of the biofilm versus planktonic time point comparisons, despite showing the highest transcript levels during late log and stationary planktonic growth. For one of these genes, *arcC*, we subsequently verified its expression patterns using quantitative reverse transcription-PCR (qRT-PCR) and Western blotting (see Fig. S5 and S6).

**FIG 5:**
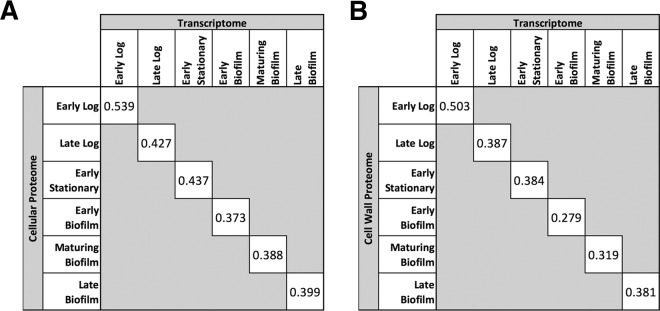
Correlation between transcriptome and proteome. Expression values within each time point were normalized by *z*-scoring, and the Pearson correlation coefficient was calculated for all genes with corresponding proteins identified in either the cellular proteome (A) or the cell wall proteome (B). The graphical representation of the full correlation between the indicated time points is available in the supplemental material.

Overall, the modest correlation between the *S. pyogenes* transcriptome and proteomes could be seen at every time point examined (Fig. 5; see also Fig. S4). All time points had Pearson correlation coefficients of less than 0.55, with the highest correlation being found at the early log time point (Fig. 5). The cellular proteome showed better correlation with the transcriptome than the cell wall proteome did with the transcriptome, and the planktonic proteomes and transcriptomes showed stronger correlations than the biofilm proteomes and transcriptomes (Fig. 5).

10.1128/mSystems.00149-16.5 Figure S4  Multiple scatter plots showing correlation between planktonic and biofilm time points. Scatter plots show the *z*-scored proteome expression values plotted against the transcriptome expression values for all possible time point combinations. All genes with corresponding proteins identified in either the cellular proteome (A) or the cell wall proteome (B) are shown. The numbers in the upper-left-hand section of each box indicate Pearson correlation coefficients. Download Figure S4, PDF file, 0.3 MB. Copyright © 2016 Freiberg et al. 2016 Freiberg et al. This content is distributed under the terms of the Creative Commons Attribution 4.0 International license.

### Differential regulation of virulence factors.

**FIG 6:**
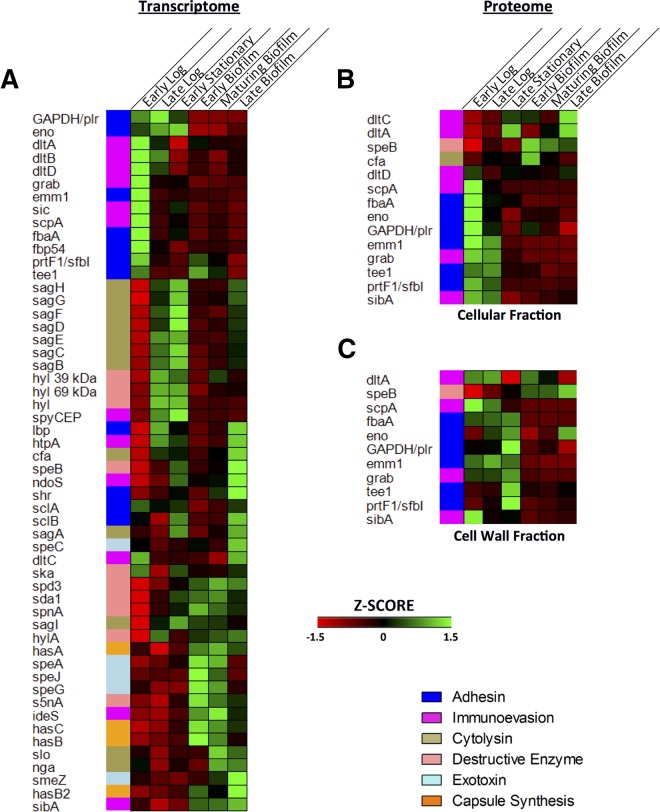
Expression profiles of GAS virulence factors during planktonic and biofilm growth. (A) *z*-scored expression values for 54 characterized and putative GAS virulence factor genes. The putative phage hyaluronidase genes are denoted with the symbol “hyl” followed by the predicted molecular mass. (B) *z*-scored expression values for the 14 of the 54 GAS virulence factors with corresponding proteomic data in the cellular fraction. (C) *z*-scored expression values for the 12 of the 54 GAS virulence factors with corresponding proteomic data in the cell wall fraction.

Based on an extensive review of the literature, we identified 52 genes that had been previously identified as *S. pyogenes* virulence factors (22–54). In addition, our transcriptome analysis revealed the transcription of 2 putative phage hyaluronidase genes. The transcriptome expression profiles for these 54 genes are shown in the heat map in Fig. 6A. It is not surprising that only three of these virulence factors (the GAPDH [glyceraldehyde-3-phosphate dehydrogenase] gene [*GAPDH*]/*plr, emm1, spyCEP*) are identified among the globally and continuously up- or downregulated genes shown in Table 1, since GAS transiently expresses its virulence factors depending on the disease stage.

A number of the virulence genes showed distinct patterns of differential expression. The majority of adhesins showed greater expression during planktonic growth, along with a number of virulence factors that help GAS avoid the innate immune system. During biofilm growth, there was greater expression of genes involved in combating the adaptive immune response, including those encoding the streptococcal superantigens. There was also increased expression of a number of genes that encode destructive enzymes during biofilm growth.

Of the 54 virulence factors identified in the transcriptome, 14 and 11 were found in the cellular and cell wall proteome samples, respectively (Fig. 6B and C). The subset of virulence factors found in the proteome samples showed expression patterns similar to what was seen in the transcriptome. Adhesins and proteins involved in defense against the innate immune response showed greater expression at the planktonic time points, as was seen in the transcriptome. The only exceptions were the proteins involved in d-alanylation of lipoteichoic acid, which showed expression patterns in the proteome that were more mixed. As was the case with the transcriptome, the expression of SpeB was greater in the biofilm proteomes, and expression increased as the biofilm aged. This difference in SpeB expression was verified by both qRT-PCR and Western blotting (see Fig. S5 and S6).

10.1128/mSystems.00149-16.6 Figure S5  Real-time RT-PCR measurement of gene expression. Expression of the *speB*, *arc*, and *emm1* gene transcriptions were measured in total cellular RNA extracts from early log planktonic, late log planktonic, early stationary planktonic, early biofilm (8-h), maturing biofilm (16-h), or late biofilm (10-day) cultures. Transcript levels are represented as the ratio of expression at a given time point to expression in early log phase. Error bars indicate standard errors. Download Figure S5, PDF file, 0.2 MB. Copyright © 2016 Freiberg et al. 2016 Freiberg et al. This content is distributed under the terms of the Creative Commons Attribution 4.0 International license.

10.1128/mSystems.00149-16.7 Figure S6  Western blotting of total cellular protein extracts. A 1-µg volume of total cellular protein extracted from an early log planktonic (lane 1), late log planktonic (lane 2), early stationary planktonic (lane 3), late stationary planktonic (lane 4), early (8-h) biofilm (lane 5), maturing (16-h) biofilm (lane 6), or late (10-day) biofilm (lane 7) culture was separated by SDS-PAGE. Gels were either stained with Coomassie blue as a control (A) or transferred to a polyvinylidene difluoride (PVDF) membrane and probed with anti-SpeB (B) or anti-ArcC (C) antibody. Download Figure S6, PDF file, 0.1 MB. Copyright © 2016 Freiberg et al. 2016 Freiberg et al. This content is distributed under the terms of the Creative Commons Attribution 4.0 International license.

## DISCUSSION

As this was the first study to comprehensively and globally characterize both the transcriptome and the proteome of *in vitro* GAS biofilms, our results give new insight into gene expression and protein production in the context of a biofilm. Despite evidence for differential regulation of more than 50% of both the transcriptome and the identified proteome at some point during biofilm growth, only a handful of genes and proteins could be classified as having biofilm-specific expression patterns. Many of these genes and proteins are either uncharacterized or unappreciated for their role in GAS biofilms. This suggests that our study achieved its main goal of opening up new avenues of understanding for GAS biofilms.

In addition, a number of virulence factors showed expression differences during biofilm growth. The majority of adhesins were upregulated during planktonic growth but had lower expression throughout biofilm growth in both the transcriptome and proteome. This list includes M protein encoded by the *emm* gene, an important and well-studied virulence factor with multiple functions (55). One of the primary roles of the M protein is attachment to host tissues in an infection (56). Although the M protein has previously been shown to be required for biofilm formation in an M14 strain, the same study found that expression of its transcript was downregulated during biofilm growth compared to exponential or stationary planktonic growth (3). Decreased expression of the *emm* transcript during biofilm growth was also reported in a more recent study utilizing an M3 strain (8). While the M protein and other adhesins are likely involved in initial attachment during biofilm growth, they appear to be downregulated at later biofilm time points. Given that the earliest biofilm time point examined in our study was 8 h after inoculation, it is possible that these adhesins were transiently expressed early and then quickly downregulated in the majority of the biofilm before sampling ever occurred.

As was seen both in our study (Fig. 6; see also Fig. S5 and S6 in the supplemental material) and in the earlier work on the GAS biofilm transcriptome (3), expression of the cysteine protease SpeB was higher during biofilm growth than during planktonic growth. This elevated expression of SpeB mimics patterns of SpeB expression seen in soft tissue infections (3, 57). Although overexpression of SpeB has been shown to lead to decreased biofilm formation (7, 15), the increased expression of SpeB during the late stages of biofilm growth may represent an important mechanism for biofilm dispersal. As suggested by work done with a murine model of a GAS biofilm infection, increased SpeB expression led to greater biofilm dispersal and disease dissemination (4).

Despite these differences in the expression of virulence factors, the most significant differences between biofilm and planktonic growth were in genes and proteins involved in metabolism (Fig. 2). This result is similar to what was found in the only previous study examining the GAS biofilm transcriptome (3). In addition, studies analyzing the biofilm transcriptome or proteome of other Gram-positive bacteria have also found differential expression of a number of genes or proteins involved in metabolism (58–65). Given that a biofilm represents a dramatically different approach to growth and requires radically different strategies of nutrient acquisition (66), it is not surprising that these studies have found strong differences in expression patterns in metabolism genes.

As expected, the correlation between the GAS transcriptome and proteome was modest. Other studies have found that the correlation between bacterial transcriptomes and proteomes is highly variable based on the experimental conditions being tested, with correlation coefficients ranging from 0.41 to 0.73 (67–75). Although a moderate correlation was seen at the early log time point (0.539 for the transcriptome versus the cellular proteome, 0.503 versus the cell wall proteome), the correlation rapidly decreased for later planktonic time points and was weak for all of the biofilm time points (Fig. 5). The fact that the strongest correlations were found at the earliest planktonic time points was unsurprising. Bacterial cells in this stage of growth express transcripts that are quickly translated for proteins needed by the cell. These cells also lack high amounts of the pervasive proteins that are produced in other growth phases but are not yet degraded. As growth progresses and both protein products and cellular waste accumulate, the cells and their environment become more complex. This change can be expected to lead to a greater divergence between the transcriptome and proteome.

We believe that the lower correlation seen in the biofilm samples is explained by the additional element of temporospatial heterogeneity that exists within a complex bacterial community. The most metabolically and transcriptionally active cells in a biofilm tend to reside in the outer layers of a biofilm (76). Because bacterial mRNA has an average half-life of less than 10 min (77, 78), the transcriptional profile of the bacteria in the outer layers is overrepresented in the transcriptome. Bacterial proteins have a significantly longer average half-life, bordering on the order of days (79). The half-life for individual proteins, however, is highly variable, and this variation in protein half-life has been shown to account for the majority of the disagreement between the results from bacterial transcriptomes and from proteomes (72). Since we sampled the entirety of the biofilm at once without regard for spatial structure, the proteomic profile that we observed was more representative of the collection of stable, accumulated proteins throughout the biofilm growth process whereas the transcriptomic profile was more representative of recent transcription in the outer layers of the biofilm.

Despite these differences between the GAS proteome and transcriptome, this study demonstrated the benefit of examining these two datasets in conjunction. Label-free liquid chromatography-tandem mass spectrometry provides an excellent tool for measuring differences in protein expression, which is a better approximation of functionally relevant changes than transcript levels. However, our proteomic analysis was still limited to those proteins that we were able to identify and quantify. Although fractionating the proteome into cell wall and cellular samples increased the number of proteins that we could identify by approximately 10%, we were still able to identify only roughly one-third of the predicted GAS proteome. This level of coverage is comparable to that obtained when the proteome of *S. pyogenes* M1 strain SF370 was probed using shotgun LC-MS/MS. Okamoto and Yamada identified 567 proteins by analyzing three different cellular fractions under three different sets of planktonic growth conditions (80).

The gaps in our proteomic data set were apparent for a number of the well-studied GAS virulence factor genes (shown in Fig. 6) whose protein products were not apparent in the proteome. As many of the GAS virulence factors are secreted proteins, specific fractionation and recovery of proteins from the culture supernatant would have likely increased our proteome coverage. Nevertheless, the majority of the virulence factors identified in the proteome fractions showed similar expression patterns in the transcriptome. While our study comprehensively characterized gene expression and protein production of GAS biofilms *in vitro*, questions still remain about the correlation to *in vivo* expression patterns. Although future studies are necessary to fully understand the relationship between the *in vitro* biofilm and *in vivo* global expression, on the basis of earlier work, we believe that *in vitro* GAS biofilms provide a useful model. Using immunoproteomics, we previously identified 28 immunogenic proteins expressed *in vivo* during a biofilm-mediated GAS infection (9). Of those 28 proteins, 26 were also identified by LC-MS/MS in the present study. We found that 15 (58%) of those 26 proteins had significantly higher expression during *in vitro* biofilm growth, while only 6 (23%) had higher expression in planktonic growth. This correlation suggests that the GAS *in vivo-*expressed proteome matches the *in vitro* biofilm proteome better than it matches the *in vitro* planktonic proteome.

In taking a global approach to understanding the GAS biofilm phenotype, we have identified a number of previously ignored genes that may contribute to *S. pyogenes* biofilm growth. In addition, as this was the first study comparing the GAS transcriptome with its proteome under any growth condition, our results demonstrate that nontranscriptional mechanisms likely play a substantial role in determining protein abundance for the majority of GAS genes. This work provides a framework to reach a better understanding of the control of protein expression in GAS biofilms.

## MATERIALS AND METHODS

### Bacterial strain and growth conditions.

For this study, GAS strain 5448 was used. Strain 5448 is an M1T1 strain representative of the clone circulating globally, which has been previously described (81). For all experiments involving liquid culture, GAS was grown at 37°C in Todd-Hewitt broth (BD Laboratories) supplemented with 0.2% yeast extract (Sigma) and then diluted to 1:5 in H_2_O (1:5 THY-B).

Planktonic cultures were inoculated from an overnight culture of GAS. The overnight culture was diluted 1:100 in side-arm flasks containing 1:5 THY-B. Growth in side-arm flasks allowed the monitoring of optical density without addition of additional oxygen to the culture. Samples from planktonic cultures were harvested at 4, 6, 8, and 48 h after inoculation, which corresponded to early log phase, late log phase, early stationary phase, and late stationary phase, respectively.

Biofilm cultures were grown as previously described (9). Briefly, an overnight culture of GAS was diluted 1:100 into prewarmed THY-B and incubated at 37°C until exponential growth began. The exponential-phase culture was inoculated into a continuous flow reactor system (82) containing 1:5 THY-B and was allowed to rest without flow for 3 h before flow was restored at a rate of 0.8 ml/min. Samples from biofilm cultures were harvested from the silicone tubing in the flow reactor at 8 h, 16 h, 6 days, and 10 days after flow was restarted, which corresponded to an early biofilm, a maturing biofilm, a mature biofilm, and a late-stage biofilm, respectively, as determined by microscopic analysis.

### Sample collection.

At the designated time points, separate aliquots were collected from the cultures for transcriptomic and proteomic analysis. Aliquots to be used for transcriptomic analysis were harvested by combining the sample with RNAprotect Bacteria reagent (Qiagen) in a 1:1 ratio and then centrifuging the sample for 10 min at 4,000 × *g* and 4°C. The resulting pellets were resuspended in 1 ml RNAprotect and frozen at −80°C until RNA extraction could be performed. Aliquots to be used for proteomic analysis were harvested by centrifuging the sample for 10 min at 4,000 × *g* and 4°C. The resulting pellet was resuspended in 1 ml of ice-cold protein preservation solution (PPS; 2.8 mM phenylmethylsulfonyl fluoride [PMSF], 50 mM Tris-Cl, 1 mM EDTA [pH 8.0], and 0.01% sodium azide). Samples were recentrifuged for 1 min at 16,000 × *g* and 4°C. The resulting supernatant was discarded, and the cell pellets were frozen at −20°C until protein extraction could be performed.

### RNA isolation.

RNA was isolated as previously described (83). Briefly, RNA was extracted from frozen cell pellets using a Direct-zol RNA Miniprep kit (Zymo Research) with the addition of an extra step for cell disruption using glass beads. The quality and concentration of the isolated RNA were verified both by gel electrophoresis and by using a NanoDrop spectrophotometer (Thermo Scientific). Due to the inability to isolate high-quality RNA from any of the late stationary planktonic samples, this time point was not included in the transcriptomic analysis. Genomic DNA was removed from the remaining total RNA samples using a Turbo DNA-free kit (Ambion). rRNA was removed from the remaining sample using a Ribo-Zero Gram-positive Bacteria rRNA removal kit (Epicentre Technologies) and purified with an Agencourt RNAClean XP kit (Beckman Coulter, Inc.). cDNA libraries were prepared from the purified RNA using a Epicentre ScriptSeq v 2 RNA-seq library preparation kit (Epicentre Technologies). The resulting cDNA was purified using an Agencourt AMPure XP system (Beckman Coulter, Inc.), and then quality and quantity were verified using an Agilent 2100 Bioanalyzer (Agilent Technologies).

### RNA sequencing.

The resulting cDNA libraries were submitted to the University of Maryland Institute for Bioscience and Biotechnology Research (UM-IBBR) Sequencing Facility located at the University of Maryland—College Park. Sequencing data in the Sanger FastQ format were generated using an Illumina HiSeq 1500 system in rapid-run mode (100-nucleotide [nt], single-end reads). Biological triplicates were sequenced for each time point.

### Transcriptome bioinformatic analysis.

RNA sequencing datasets in FastQ format were analyzed for quality using FastQC (http://www.bioinformatics.babraham.ac.uk/projects/fastqc/) (84). Reads were trimmed and Illumina adapters were clipped using Trimmomatic v 0.32 (85) with a leading and trailing minimum score of 3 and a 4-base sliding window minimum score of 15, which resulted in an average of 99.98% of reads surviving (range, 99.93% to 99.99%). Reads were mapped to the GAS MGAS5005 genome (NC_007297.1; NCBI) using Bowtie2 v 2.2.4 (86) run in end-to-end mode with default settings for an average overall alignment rate of 98.80% (range, 95.72% to 99.38%). Transcript abundances were calculated in fragments per kilobase per million mapped reads (FPKM) using Cufflinks v 2.2.1 (87) with a ribosomal masking file for all 5S, 16S, 23S, and tRNA loci (NC_007297.1.gff; NCBI). Cuffdiff (88), a program within the Cufflinks package, was used to calculate differential expression values for genes with an FDR-adjusted *P* value (*q* value) of less than 0.01. Operon structure was predicted from the resulting Bowtie2 alignment files using Rockhopper v 2.03 (89, 90).

### Protein isolation.

The cell wall and cellular protein fractions from each protein sample were isolated separately. The cell wall protein fraction was isolated using PlyC, a bacteriophage lysin previously shown to be effective in isolating cell wall proteins from *S. pyogenes* (91). Briefly, the frozen cell pellets were resuspended in 1 ml PlyC lysis buffer (50 mM ammonium acetate [pH 5.2], 5 mM EDTA, and Roche Complete protease inhibitors). Equal numbers of cells from the samples, as determined by the optical density at 600 nm, were transferred to fresh tubes. The cells were pelleted and resuspended in 1 ml lysis buffer containing 40% (wt/vol) sucrose and 1 µg/ml PlyC. Cells were digested for 1 h at 37°C with constant rotation and then centrifuged for 8 min at 16,000 × *g*. The resulting supernatant containing the cell wall protein fraction was separated from the pelleted protoplasts containing the cellular protein fraction. The supernatant was recentrifuged for 1 min at 16,000 × *g*, and the supernatant from this second centrifugation step was used as the cell wall fraction. The pelleted protoplasts containing the cellular fraction were then resuspended in 1 ml PlyC lysis buffer (without sucrose). The protoplasts were lysed by adding 0.7 g of 0.1-mm-diameter silica beads to the sample and then beating the samples using a FastPrep instrument.

The protein concentrations in the cell wall and the cellular protein fractions were determined using an Advanced protein assay (Cytoskeleton, Denver, CO). A 20-µg volume of each protein sample was subsequently purified by trichloroacetic acid (TCA) precipitation. The precipitated proteins were then rehydrated in 250 µl of rehydration buffer {7.5 mM TCEP [tris(2-carboxyethyl)phosphine], 8 M urea, 100 mM ammonium bicarbonate} at 37°C for 1 h. After removing the rehydration buffer by centrifuging the samples in a 3-kDa-molecular-mass-cutoff filter (Sigma), the samples were alkylated by adding 250 µl of alkylation buffer (500 mM iodoacetamide, 8 M urea, 100 mM ammonium bicarbonate) for 1 h at room temperature. The samples were then washed with 50 mM ammonium bicarbonate by centrifugation in a 3-kDa-molecular-mass-cutoff filter and then subjected to trypsin digestion at 37°C using 1 µg of mass spectrometry-grade Trypsin Gold (Promega). After 12 h, 10% trifluoroacetic acid was added to the trypsin-digested protein samples to acidify the samples to a pH of less than 5 and to prevent further digestion.

### LC-MS/MS.

Quantitative proteomics data for all of the biofilm samples, along with the early log, late log, and late stationary planktonic samples, were generated by electrospray ionization in the positive ion mode on a hybrid quadrupole-Orbitrap mass spectrometer, Q Exactive (Thermo Scientific). Proteomics data for early stationary planktonic samples were generated using a Thermo Orbitrap Elite Hybrid Ion Trap-Orbitrap mass spectrometer (Thermo Scientific). Nanoflow high-pressure liquid chromatography (HPLC) was performed by using a Waters NanoAcquity HPLC system (Waters Corporation, Milford, MA). Peptides were trapped on a fused-silica precolumn (inner diameter [i.d.], 100 μm; o.d., 365 μm) packed with 2 cm of 5-μm-diameter (200-Å) Magic C_18_ reverse-phase particles (Michrom Bioresources, Inc., Auburn, CA). Subsequent peptide separation was conducted on a 75-μm-i.d.-by-180-mm-long analytical column constructed in-house using a Sutter Instruments P-2000 CO_2_ laser puller (Sutter Instrument Company, Novato, CA) and packed with 5-μm-diameter (100-Å) Magic C_18_ particles. Mobile phase A consisted of 0.1% formic acid–water, and mobile phase B consisted of 0.1% formic acid–acetonitrile. Peptide separation was performed at 250 nl/min in a 95-min run. Mobile phase B started at 5% and increased to 35% at 60 min and then 80% at 65 min, followed by a 5-min wash at 80% and a 25 min re-equilibration at 5%. Ion source conditions were optimized by using the tuning and calibration solution recommended by the instrument provider. Data were acquired by using Xcalibur (version 2.8; Thermo Scientific). MS data were collected by top-15 data-dependent acquisition. A full MS scan (range, 350 to 2,000 *m*/*z*) was performed with 60-K resolution in an Orbitrap followed by collision-induced dissociation (CID) fragmentation of precursors in an ion trap at a normalized collision energy level of 35. Technical triplicates of biological duplicates were analyzed for each time point.

### Proteome bioinformatic analysis.

The MS datasets were searched against a *S. pyogenes* serotype M1 database (UniProt) using the Andromeda search engine (92) from the MaxQuant software package (93). A bottom-up approach was employed, and MS1 peak intensity was used for the peptide quantification. MaxQuant LFQ values, which take MS1 peak intensity (extracted ion current) information, were used for the peptide quantification. Protein abundance profiles were assembled using the maximum possible information from MS signals, given that the presence of quantifiable peptides varies from sample to sample. Permutation-based methods for calculating *q* values and global FDRs were applied (94). Search results were filtered with a false-discovery-rate cutoff of 0.01. Label-free quantification (LFQ) was performed using MaxQuant (94). Because LC-MS/MS was performed on the early stationary proteomic samples using a different mass spectrometer, we were unable to include this time point in the LFQ analysis with the rest of the samples. Data from the early stationary time point were analyzed in a second, separate MaxQuant LFQ analysis and were therefore not adequate for comparison to the proteomic data from the other time points. Perseus v 1.5.1.6, a software package for shotgun proteomics data analysis (http://www.perseus-framework.org/), was used to calculate differential expression from the resulting LFQ intensity values. Differential expression values with a false-discovery-rate-adjusted *P* value (*q* value) of less than 0.01 were considered significant.

### Accession number(s).

The RNA-seq data and analysis discussed in this publication were deposited in the NCBI Gene Expression Omnibus (GEO) database under accession number GSE80659.

10.1128/mSystems.00149-16.1 Text S1  Supplemental Materials and Methods. Download Text S1, PDF file, 0.1 MB. Copyright © 2016 Freiberg et al. 2016 Freiberg et al. This content is distributed under the terms of the Creative Commons Attribution 4.0 International license.
