# Supplementary material for: Global Analysis and Comparison of the Transcriptomes and Proteomes of Group A Streptococcus Biofilms
Source: mSystems. 2016 Dec 6;1(6):e00149-16. doi: 10.1128/mSystems.00149-16 (PMC5141267; doi:10.1128/mSystems.00149-16)
Supplement: Figure S1 [file sys006162066sf2.pdf]

Supplemental Results

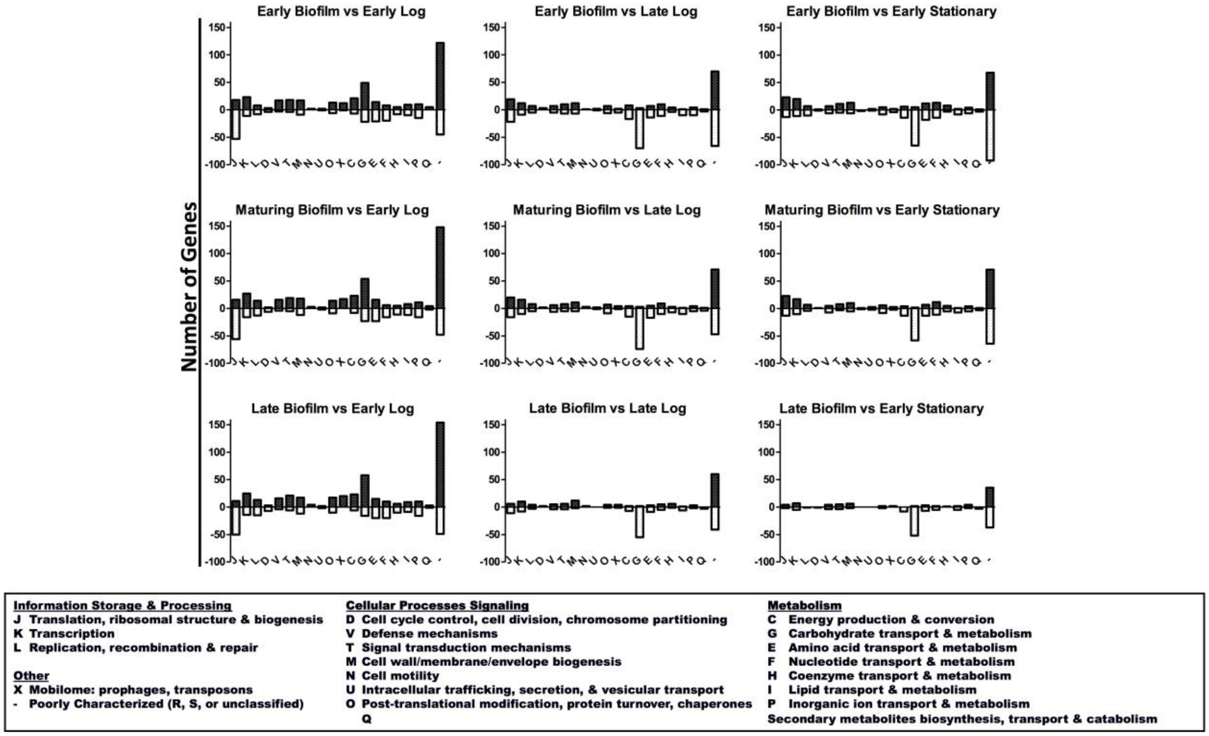

Figure S1. Differential regulation of biofilm versus planktonic transcriptome according to COG classifications. The numbers of genes in each COG differentially regulated at each biofilm versus planktonic time point are shown. Dark bars indicate the number of genes in the COG up-regulated and light bars indicate the number of genes down-regulated. COGs were analyzed with the R-package BOG (21) to identify COGs with a statistically greater than expected number of genes showing differential expression. *=adj. p value < 0.05 according to the Mann-Whitney Rank Sum test. The “Poorly Characterized” group includes COG classifications R (general function prediction only) and S (unknown function) in addition to unclassified genes.
