# Supplementary material for: Global Analysis and Comparison of the Transcriptomes and Proteomes of Group A Streptococcus Biofilms
Source: mSystems. 2016 Dec 6;1(6):e00149-16. doi: 10.1128/mSystems.00149-16 (PMC5141267; doi:10.1128/mSystems.00149-16)
Supplement: Table S1 [file sys006162066st8.pdf]

Table S1. Mean LFQ intensities for cellular protein fraction

	LFQ Intensity (Log_2 Value ± Standard Deviation)					
M5005 Locus	Early Log	Late Log	Late Stationary	Early Biofilm	Maturing Biofilm	Late Biofilm
**Spy0001**	24.86±0.0245	24.29±0.4117	24.29±0.1541	24.45±0.2453	24.5±0.3067	24.58±0.5251
**Spy0002**	27.97±0.0942	27.7±0.2607	27.66±0.2353	27.54±0.0935	27.32±0.0273	27.55±0.2942
**Spy0004**	26.89±0.0191	27.59±0.1875	27.25±0.1527	26.45±0.1268	26.47±0.0018	26.75±0.3257
**Spy0012**	24.85±1.2478	26.73±0.1806	27.42±0.0213	27.06±0.0118	27.14±0.039	28.61±0.2622
**Spy0013**	30.55±0.1162	30.7±0.2657	30.89±0.1866	30.31±0.2168	30.33±0.0284	30.84±0.1276
**Spy0017**	26.41±0.5322	26.31±0.3628	25.36±0.1559	25.51±0.5761	25.77±0.1227	25.56±0.6648
**Spy0018**	28.82±0.0808	28.13±0.3295	27.86±0.187	27.71±0.4789	27.66±0.3056	27.81±0.2408
**Spy0020**	24.6±0.0627	25.66±0.0743	26.71±0.1517	23.85±0.2084	24.24±0.1748	26.24±0.4414
**Spy0021**	23.65±0.5614	23.96±0.1502	25.61±0.0941	24.46±0.0096	24.58±0.0485	24.67±0.1929
**Spy0033**	25.59±0.5937	25.59±0.4978	24.15±0.1152	26.5±0.6141	26.3±0.2582	25.87±0.5245
**Spy0039**	26.18±1.347	28.85±0.1868	29.69±0.2541	27.71±0.2128	28.08±0.5971	29.2±0.0575
**Spy0040**	24.17±0.4173	25.5±1.0605	25.38±0.2251	27.66±0.0661	27.93±0.4976	26.19±0.6195
**Spy0043**	30.57±0.3301	30.91±0.5663	31.63±0.3099	30.46±0.4628	30.01±0.1094	30.49±0.1537
**Spy0044**	30.88±0.3602	30.11±0.3325	30.3±0.0929	30.14±0.19	30±0.0449	29.58±0.7017
**Spy0045**	30.3±0.1617	30.43±0.0467	31.15±0.0531	29.72±0.2041	29.6±0.0498	30.5±0.273
**Spy0046**	29.41±0.3603	29.5±0.4848	29.85±0.3373	29.06±0.5965	28.85±0.4563	29.25±0.2727
**Spy0047**	32.99±0.432	32.35±0.4882	32.87±0.0437	32.65±0.157	32.64±0.088	32.54±0.0125
**Spy0048**	30.62±0.8923	29.97±0.5955	30.89±0.1357	30.36±0.0645	31.08±0.2571	30.45±0.9166
**Spy0049**	30.75±0.2979	30.74±0.7416	31.2±0.3556	30.97±0.3737	30.55±0.0828	30.47±0.2176
**Spy0050**	30.26±0.1461	30.57±0.0216	31.21±0.0115	29.78±0.0177	29.74±0.0202	30.53±0.2553
**Spy0051**	28.88±0.1118	28.57±0.0743	28.93±0.023	28.62±0.3737	28.34±0.4526	28.47±0.2206
**Spy0052**	28.53±1.1554	27.43±0.7266	26.93±1.9016	26.98±1.1572	27.42±1.0539	26.89±2.5178
**Spy0053**	30.09±0.4662	28.36±0.9459	27.16±0.1712	27.39±0.5917	26.66±0.2075	28.11±1.8885
**Spy0054**	30.06±0.7863	29.46±0.5113	29.92±0.4227	29.5±0.9698	29.91±0.8079	29.64±0.3487
**Spy0055**	31.97±0.1259	31.03±0.099	31.35±0.2747	31.03±0.1653	31.23±0.4343	31.07±0.0371
**Spy0056**	30.49±0.0652	30.85±0.2527	31.62±0.0468	30.29±0.1106	29.89±0.1663	30.64±0.1523
**Spy0057**	29.37±0.2041	28.74±0.1798	28.93±0.7956	28.31±0.7207	28.17±0.7143	29.09±0.4232
**Spy0058**	29.7±0.6244	30.1±0.4919	30.85±0.4359	29.36±0.4474	29.23±0.2482	29.63±0.4984
**Spy0059**	31.01±0.4111	30.88±0.6273	31.27±0.3739	30.49±0.6159	30.14±0.0471	29.91±0.1771
**Spy0060**	31.42±0.2487	30.61±0.1372	30.73±0.2095	31.1±0.0458	30.99±0.1556	30.53±0.461
**Spy0061**	30.31±0.3116	30.77±0.2919	31.51±0.1427	30.01±0.1903	30.01±0.0436	30.53±0.355
**Spy0062**	28.78±0.4432	29.09±0.1362	29.7±0.0557	28.92±0.1312	28.62±0.1251	28.5±0.0853
**Spy0063**	32.63±0.3075	31.64±0.0929	32.06±0.1376	31.96±0.2562	31.76±0.3992	31.57±0.6254
**Spy0065**	25.09±0.7501	26.82±0.1733	27.39±0.1372	27.96±0.0416	27.82±0.1303	27.34±0.1785
**Spy0066**	27.6±0.0414	26.94±0.1345	28.19±0.1711	27.32±0.0084	27.31±0.2324	27.09±0.0261
**Spy0067**	30.8±0.0816	30.55±0.2045	30.97±0.1251	30.43±0.3385	30.37±0.7297	30±0.0331
**Spy0068**	30.68±0.1007	30.58±0.6262	31.44±0.3835	31.11±0.4494	30.78±0.0046	30.79±0.1223
**Spy0069**	28.9±0.5022	28.59±0.4307	29.01±0.0453	28.82±0.1474	29.02±0.0141	28.8±0.2864
**Spy0070**	30.91±0.6439	30.76±0.3034	30.6±0.3129	30.4±0.6914	30.29±0.442	30±0.2769

**Spy0071**	32.72±0.3476	32.23±0.0872	32.03±0.1268	32.23±0.0794	31.9±0.2494	31.9±0.4676
**Spy0083**	31.53±0.6907	30.95±0.0587	30.73±0.023	30.54±0.1189	30.52±0.0133	30.39±0.2946
**Spy0084**	31.19±0.6846	30.55±0.1485	30.37±0.1812	30.07±0.276	30.01±0.0555	29.83±0.2944
**Spy0094**	27.34±0.3284	27.97±0.1074	28.03±0.1141	27.96±0.4857	28.14±0.2829	28.57±0.2578
**Spy0097**	27.28±0.4554	26.66±0.4623	26.15±0.7989	26.79±0.7322	26.24±1.1245	27.23±0.5045
**Spy0104**	23.63±0.3585	23.85±0.4077	23.31±0.0303	24.36±0.0106	23.69±0.5202	23.46±0.4039
**Spy0105**	25.31±0.3459	25.39±0.1596	26.12±0.2045	24.93±0.7165	24.67±0.9792	25.87±0.19
**Spy0107**	27.87±0.5375	27.03±1.4051	26.69±0.3328	26.28±1.1195	25.89±1.4379	26.51±0.7518
**Spy0109**	27.05±0.0167	26.25±1.7832	26.03±0.2908	25.64±1.3205	25.29±0.6246	25.47±0.8727
**Spy0122**	24.2±0.3419	25.3±0.0813	24.28±0.6041	24.13±0.099	23.63±0.3444	24.24±0.0861
**Spy0134**	26.56±0.0003	26.87±0.0569	27.19±0.2283	25.67±0.3623	25.97±0.231	26.17±0.0667
**Spy0136**	30.45±0.5737	30.11±0.9246	30.24±0.4156	30.16±1.0802	30.39±0.8281	30.38±0.1564
**Spy0137**	29.74±0.2644	28.91±0.2607	26.96±0.4162	28.44±0.064	28.18±0.0054	27.83±0.1734
**Spy0138**	27.56±0.222	27.97±0.0531	28.27±0.1396	27.64±0.0487	27.61±0.1593	27.99±0.0951
**Spy0147**	25.39±1.0358	27.34±0.0021	29.13±0.12	26.72±0.174	27.37±0.069	28.48±0.3555
**Spy0157**	25.45±0.0358	25.04±0.2013	24.9±0.0672	25.2±0.3106	25.34±0.2477	25.4±0.1107
**Spy0159**	24.74±0.2499	25.23±0.5488	26.01±0.4708	24.38±0.1583	23.8±0.1923	26±0.2579
**Spy0161**	24.28±0.9419	24.49±0.8928	24.97±0.2657	23.81±0.336	24.3±0.1458	24.86±0.7179
**Spy0164**	25.64±0.1868	25.45±0.0434	24.55±0.0108	24.06±0.3803	24.15±0.3523	24.35±0.1175
**Spy0175**	26.38±0.4379	26.27±0.1959	25.71±0.8541	26.42±0.9536	26.44±0.9891	26.73±0.32
**Spy0178**	25.91±0.2488	26.22±0.2945	25.14±0.2399	27.23±0.722	27.1±0.3631	25.92±0.3
**Spy0185**	31.56±0.1484	30.8±0.1335	29.46±0.1894	30.96±0.133	30.65±0.1923	29.59±0.1506
**Spy0192**	27.48±0.2288	28.07±0.1101	28.07±0.0067	27.28±0.088	27.48±0.2357	27.95±0.2288
**Spy0194**	27.54±0.5129	27.84±0.0539	28.2±0.1201	27.56±0.5424	28.31±0.3486	28.4±0.3916
**Spy0199**	24.88±0.2469	24.75±0.2657	23.58±0.4498	25.19±0.1824	24.49±0.6008	24.26±0.1153
**Spy0203**	28.22±0.1914	28.76±0.0476	29.01±0.0438	28.08±0.278	28.15±0.2029	28.75±0.1817
**Spy0209**	29.43±0.0239	29.16±0.0316	28.96±0.265	28.68±0.1041	28.22±0.1018	27.91±0.1639
**Spy0222**	23.79±0.0352	24.6±0.2818	26.39±0.4838	24.24±0.3449	23.41±0.0338	25.39±0.4916
**Spy0224**	27.07±0.5886	27.4±0.2395	25.7±0.7399	28.15±0.6429	27.91±0.6433	27.4±0.5919
**Spy0226**	24.74±0.1742	25.33±0.3233	25.33±0.2598	23.92±0.173	24.08±0.1022	24.04±0.4489
**Spy0227**	25.41±0.5683	26.15±0.2887	26.16±0.083	25.51±0.4084	25.67±0.3214	26.34±0.0162
**Spy0228**	26.37±1.6969	27±0.8224	25.45±0.6058	25.91±0.5834	25.21±0.5805	24.98±0.5063
**Spy0229**	25.61±0.2832	23.99±0.032	23.92±0.0836	24.3±0.0074	23.61±0.2759	24.31±0.5116
**Spy0230**	29.05±0.112	28.79±0.3646	29.71±0.3347	28.87±0.0326	28.71±0.1583	29.57±0.0968
**Spy0231**	29.38±0.8429	29.68±1.0114	30.2±0.492	29.67±0.7992	29.04±0.585	29.62±0.2523
**Spy0232**	31.42±0.1475	32.26±0.0057	32.82±0.018	31.75±0.0374	32.07±0.0437	32.6±0.238
**Spy0233**	35.82±0.4389	35.02±0.0547	34.19±0.3528	34.99±0.4327	34.55±0.3325	33.4±0.1053
**Spy0235**	25.81±0.4886	26.94±0.3658	25.82±0.2663	24.96±0.1294	24.62±0.1628	25.39±0.2393
**Spy0236**	23.98±0.4833	23.62±0.3755	24.07±0.12	24.09±0.2087	23.77±0.6356	23.78±0.0136
**Spy0239**	24.77±0.2781	24.3±0.0613	23.9±0.1114	24.52±0.4692	23.97±0.213	24.42±0.4762
**Spy0242**	23.9±0.4947	24.72±0.4366	26.23±0.2473	25.37±0.3124	25.65±0.0946	26.88±0.0518
**Spy0243**	24.1±0.1458	24.4±0.2214	25.75±0.2223	24.61±0.2186	25.13±0.2251	25.83±0.4299
**Spy0244**	23.98±0.2991	23.99±0.1319	23.98±0.3202	24.66±0.8535	24.56±0.794	23.29±0.002

**Spy0246**	23.88±0.3383	23.57±0.1674	23.86±0.0491	23.98±0.1721	23.51±0.4788	24.19±0.1048
**Spy0249**	29.6±0.0224	29.33±0.3919	28.14±0.3251	27.37±0.1555	27.05±0.0839	26.81±0.1459
**Spy0252**	24.95±0.3198	25.72±0.2964	25.64±0.0342	24.35±0.6891	24.44±0.3985	25.7±0.2125
**Spy0253**	25.6±0.5358	26.09±0.0058	25.54±0.317	23.9±0.0676	24.07±0.108	25.99±0.0683
**Spy0263**	24.42±0.6639	24.05±0.4996	23.86±0.2814	23.91±0.1324	23.96±1.0714	24.61±0.3133
**Spy0265**	24.43±0.8904	23.92±0.6605	25.07±0.5742	23.55±0.4501	24.25±0.5153	24.47±0.5491
**Spy0269**	28.85±0.0724	29.8±0.3608	29.29±0.3689	29.91±0.2161	29.62±0.2528	29.29±0.1171
**Spy0270**	27.1±0.0261	27.62±0.2253	26.22±0.4263	28.55±0.1503	28.36±0.0466	27.43±0.0878
**Spy0272**	25.41±0.985	26.69±0.1082	26.62±0.0142	25.14±1.2605	24.84±1.9857	26.9±0.2099
**Spy0275**	24.07±0.455	23.97±0.1622	24.74±0.1646	24.18±0.0733	24.54±0.8021	23.86±0.1185
**Spy0276**	24.36±0.4009	25.04±0.134	24.9±0.0294	24.24±0.0587	24.31±0.7085	24.9±0.3209
**Spy0279**	27.33±0.0766	28.19±0.3975	27.75±0.2978	27.13±0.8189	27.15±0.2323	28.3±0.1863
**Spy0282**	30.48±0.9359	30.46±0.0881	29.81±0.1153	29.52±0.6482	29.44±0.5597	29.88±0.2598
**Spy0284**	23.73±0.1168	24.81±0.2908	24.4±0.2412	23.69±0.446	23.92±0.1551	24.32±0.1626
**Spy0286**	24.57±0.159	24.14±0.0093	24.67±0.5151	24.28±0.2621	24.11±0.8993	24.5±0.1205
**Spy0287**	26.06±0.2354	26.89±0.1366	27.19±0.2184	26.11±0.1603	26.23±0.109	26.92±0.0919
**Spy0289**	26±0.1819	25.08±0.8015	23.57±0.5981	24.86±0.932	25.27±0.4822	24.29±0.0458
**Spy0290**	24.28±0.148	25.92±0.0036	25.73±0.063	24.17±0.4466	24.81±0.8258	25.72±0.7593
**Spy0292**	26.92±0.4994	27.18±0.1662	25.35±1.6884	26.14±0.033	25.11±0.8637	24.44±0.5465
**Spy0293**	28.78±0.6992	28.61±0.2336	28.53±0.1761	29.76±0.3089	29.68±0.3037	28.76±0.4275
**Spy0306**	23.77±0.4544	23.98±0.4003	24.84±0.4715	23.4±0.032	23.84±0.0797	23.97±0.2902
**Spy0310**	25.15±1.095	24.49±0.5967	24.76±0.1036	24.33±0.0907	24.27±0.2943	23.53±0.2363
**Spy0319**	28.76±0.2089	29.53±0.1626	29.92±0.1141	29.46±0.3817	29.84±0.4406	30.24±0.1373
**Spy0327**	27.22±0.2018	27.76±0.9217	28.44±0.1351	27.61±0.9573	27.26±0.5989	27.74±0.1327
**Spy0328**	29.05±0.6309	29.03±0.2934	28.42±0.7604	28.78±0.5209	28.03±0.3104	29.13±0.7709
**Spy0329**	26.7±1.7364	27.66±1.0582	25.99±2.3346	25.42±0.7457	26.86±1.0859	25.85±0.032
**Spy0330**	23.86±0.3548	24.78±0.4038	26.27±0.754	24.28±0.2273	24.47±0.6591	25.08±0.3329
**Spy0334**	23.77±0.2412	23.88±0.0561	25.3±0.4938	23.98±0.5887	24.06±0.7212	24.23±0.1588
**Spy0337**	23.37±0.2358	24.71±1.0301	25.29±0.2451	26.19±0.8156	25.02±0.6886	26.11±0.303
**Spy0338**	27.31±0.2286	27.37±0.5431	26.04±0.0879	28.09±0.3276	27.77±0.204	26.13±0.3282
**Spy0339**	28.82±0.786	27±0.4938	23.96±0.0239	27.01±0.1355	27.04±0.1832	25.92±0.1547
**Spy0345**	26.3±0.0981	26.79±0.6047	27.25±0.5088	26.04±0.5348	26.22±0.3439	26.5±0.5085
**Spy0360**	23.45±0.7158	24.35±0.416	24.1±0.5975	24.09±0.5757	23.62±0.0628	24.09±0.2216
**Spy0362**	25.71±0.3607	26.39±0.1874	27.29±0.1049	25.54±0.1069	25.55±0.2172	26.61±0.409
**Spy0363**	24.3±0.3149	25.09±0.5633	26.13±0.4425	24.19±0.8382	24.67±0.4241	25.16±0.0938
**Spy0367**	24.06±0.3523	25.64±0.0605	25.31±0.1774	24.75±0.6118	24.86±0.5986	24.96±0.2227
**Spy0368**	28.65±0.2438	28.88±0.404	27.92±0.1133	28.26±0.4027	28.51±0.0152	28.71±0.0235
**Spy0369**	23.99±0.2955	25.64±0.338	25.81±0.0393	24.66±0.0046	24.6±0.0017	25.7±0.3405
**Spy0371**	27.03±0.2579	24.58±0.6736	24.11±0.3448	24.13±0.6835	24.86±0.222	23.85±0.2691
**Spy0374**	30.34±0.801	30.31±0.0192	30.95±0.0274	30.18±0.1655	29.92±0.5338	30.17±0.2355
**Spy0375**	29.72±0.1675	30.08±0.0188	31.08±0.2199	29.19±0.0252	29.16±0.0799	29.9±0.2884
**Spy0378**	24.35±0.0023	25.32±0.2024	26.72±0.0802	24.39±0.3876	25.03±0.1521	26.22±0.2207
**Spy0380**	28.75±0.246	28.84±0.4072	30.37±0.1952	29.8±0.0599	29.56±0.3489	29.84±0.1713

**Spy0381**	26.53±0.1884	24.52±0.2858	23.61±0.0821	25.08±0.1496	24.19±0.493	24.02±0.104
**Spy0382**	23.9±0.0867	24.01±0.0275	24.08±0.0136	24.44±0.5464	25.6±1.0297	23.55±0.2608
**Spy0384**	25.25±0.2416	24.58±1.9429	23.76±0.3898	24.07±0.2248	23.78±0.5603	23.68±0.3866
**Spy0385**	26.27±1.0362	27.59±0.001	28.44±0.1357	28.51±0.0622	28.64±0.1821	29.33±0.2195
**Spy0386**	24.02±0.1123	24.07±0.4108	24.62±0.9651	23.89±0.0448	23.99±0.0341	24.13±0.1778
**Spy0387**	25.22±0.8859	25.86±0.7621	25.6±0.4257	25.58±1.004	25±0.8429	25.62±0.2951
**Spy0388**	23.83±0.3405	23.4±0.1429	24.76±0.2445	24.17±0.172	23.61±0.3067	24.41±0.1975
**Spy0390**	24.9±0.0756	26.05±0.0399	25.87±0.0321	24.65±0.0684	24.64±0.0494	25.81±0.6546
**Spy0417**	29.78±0.0334	29.92±0.1587	29.13±0.1267	30.82±0.1541	30.42±0.1722	29.96±0.1137
**Spy0421**	26.45±1.6995	27.42±1.0741	27.23±0.5264	28.25±0.6256	28±0.5533	27.01±0.1134
**Spy0422**	24.52±0.7084	25.06±0.2028	24.55±0.0868	25.22±0.374	25.43±0.2968	25.53±0.2076
**Spy0423**	24.53±1.0295	27.14±1.0463	27.64±0.0676	26.81±1.6029	27.1±1.1941	28.13±0.2277
**Spy0424**	31.17±0.3179	30.57±0.4181	28.77±0.2293	30.85±0.1955	30.19±0.2112	28.96±0.0961
**Spy0427**	28.69±0.1211	28.92±0.3154	29.31±0.1311	28.63±0.275	28.46±0.1424	28.76±0.3596
**Spy0435**	26.97±0.3336	26.89±0.4676	25.65±0.3035	26.47±0.1103	26.35±0.0545	26.64±0.318
**Spy0438**	24.38±0.2265	24.97±0.0188	25.87±0.6511	23.76±0.5852	24.37±0.0725	24.38±0.5401
**Spy0439**	27.74±0.2002	27.13±0.05	27.42±0.1591	27.21±0.1281	26.95±0.0511	27.3±0.0452
**Spy0463**	25.92±0.1326	26.52±0.1313	27.33±0.3404	26.53±0.211	26.88±0.277	27.68±0.0494
**Spy0470**	23.2±0.3599	24.49±0.5017	24.36±0.504	24.5±0.2682	24.94±0.1497	26.39±0.9282
**Spy0472**	27.09±0.2331	27.42±0.2127	28.34±0.3489	27.43±0.0844	27.63±0.2954	28.03±0.1972
**Spy0484**	23.81±0.4489	24.02±0.3551	24.54±0.0396	23.55±0.4866	23.92±0.1634	25.51±0.1729
**Spy0487**	30.01±0.5991	29.8±0.6779	29.27±0.2545	30.28±0.8169	30.11±0.6494	29.57±0.4021
**Spy0493**	29.3±0.0913	29.34±0.6291	28.97±0.2914	30.44±0.0627	30.01±0.0054	28.7±0.4454
**Spy0495**	27.75±0.2014	28.26±0.0177	28.4±0.005	27.57±0.0455	27.75±0.1532	28.35±0.3749
**Spy0497**	23.99±0.293	24.12±0.3771	23.98±0.1876	23.46±0.167	23.82±0.0723	24.15±0.1439
**Spy0498**	24.63±0.1233	24.96±0.9315	26±0.3125	25.21±0.801	24.6±1.0133	24.51±0.0677
**Spy0503**	23.76±0.4646	24.26±0.4161	23.99±0.1295	24.2±0.3701	24.57±0.2688	24±0.1685
**Spy0504**	29.46±0.1785	29.25±0.0712	29.02±0.0635	29.5±0.2463	29.24±0.184	29.09±0.1438
**Spy0505**	24.99±0.982	23.3±0.2861	23.53±0.1893	24.18±0.9476	24.46±0.5459	24.75±1.0843
**Spy0508**	33.68±0.0804	33.8±0.3243	34.01±0.2574	33.44±0.091	34.23±0.2846	34.17±0.4012
**Spy0509**	33.53±0.3746	33.15±0.1546	32.59±0.0464	33.28±0.1735	33.65±0.0111	31.94±0.422
**Spy0516**	27.5±0.1253	27.54±0.481	27.88±0.1079	26.64±0.429	26.15±0.9132	27.29±0.2018
**Spy0517**	25.46±0.6144	26.32±0.4412	24.92±0.1394	26.33±0.1306	25.47±0.2212	25.14±0.3399
**Spy0530**	24.71±0.0632	25.74±0.0235	25.65±0.2557	25.75±0.3456	26.45±0.568	27.05±0.0153
**Spy0531**	25.78±0.7461	26.21±0.0299	25.87±0.2688	25.94±0.0565	25.77±0.2741	26.55±0.0521
**Spy0533**	23.9±0.4903	23.54±0.0588	23.5±0.3365	24±0.663	23.77±0.4105	23.95±0.2149
**Spy0538**	27.56±0.6317	28.1±0.5139	28.26±0.1622	27.48±0.5252	27.51±0.323	27.75±0.218
**Spy0539**	24.74±0.2316	26.09±0.0358	26.57±0.1264	24.53±0.1335	25.25±0.2564	26.38±0.2603
**Spy0545**	23.34±0.3308	24.43±0.0765	25.72±0.1223	24.98±1.1476	26±0.4202	27.24±0.2986
**Spy0546**	29.66±0.6608	29.4±0.0969	30.65±0.0389	29.78±0.025	30.01±0.1645	29.4±0.1128
**Spy0547**	26.12±0.1138	25.71±0.3377	26.02±0.19	25.56±0.36	25.64±0.134	26.23±0.153
**Spy0551**	31.05±0.7981	31.1±0.7022	31.58±0.2849	30.51±0.9313	30.48±0.6091	31.02±0.7651
**Spy0553**	25.72±0.0522	25.99±0.165	26.06±0.3328	24.5±0.4493	24.75±0.1147	26±0.0616

**Spy0554**	27.16±0.1947	27.9±0.2399	27.56±0.0472	27.07±0.3066	26.93±0.2669	27.13±0.2647
**Spy0556**	36.68±0.4065	35.81±0.3973	35.14±0.2637	35.61±0.5524	35.69±0.31	35.35±0.2181
**Spy0561**	31.52±0.5962	30.61±0.0313	30.57±0.0694	30.12±0.5155	30.3±0.7467	30.47±0.0955
**Spy0574**	24.74±0.2019	25.37±0.0055	24.38±0.3801	24.44±0.1408	23.93±0.0674	24.9±0.7907
**Spy0577**	28.04±0.3768	28.61±0.8855	28.08±0.147	26.65±1.3559	26.94±0.5635	26.74±0.9557
**Spy0578**	25.31±0.3222	24.89±0.7015	25.08±0.3629	24.41±1.2383	25.2±0.2108	23.79±0.4916
**Spy0579**	32.29±0.5057	32.45±0.4872	31.03±0.1624	31.75±0.3871	31.58±0.1137	30.83±0.3995
**Spy0580**	28.76±0.7737	29.07±0.3149	27.65±0.055	28.23±0.14	28.03±0.2718	27.61±0.4296
**Spy0581**	32.37±0.4077	32.38±0.6008	30.87±0.2586	31.68±0.3956	31.5±0.1269	30.86±0.2466
**Spy0582**	29.67±0.7717	29.59±0.1053	28.58±0.218	29.05±0.258	28.86±0.4292	27.98±0.401
**Spy0584**	24.51±0.1186	24.61±0.5526	25.34±0.1403	25.08±0.6357	25.22±0.7148	25.19±0.6235
**Spy0587**	24.1±0.3031	23.78±0.1729	24.07±0.3945	23.62±0.2785	23.82±0.1864	24.7±0.1225
**Spy0588**	25.14±0.5472	27.04±0.171	28.01±0.3102	25.74±0.1891	26.39±0.3774	27.4±0.1403
**Spy0597**	31±0.5175	30.05±0.6752	31.07±0.0139	30.42±0.0676	30.47±0.5031	30.26±0.4339
**Spy0598**	26.76±0.2656	27.87±0.9202	27.62±0.2093	27.85±0.4876	27.7±0.3022	27.1±0.6002
**Spy0600**	26.73±0.2741	26.82±0.2863	27.43±0.5018	27.08±0.4716	26.94±0.1146	26.69±0.2122
**Spy0602**	25.54±1.5788	27.01±0.6909	27.45±0.0808	26.99±0.5912	26.85±0.488	27.29±0.1144
**Spy0606**	25.36±0.3569	26.22±0.2178	26.18±0.2789	25.51±0.0415	25.23±0.4828	25.97±0.1748
**Spy0614**	24.26±0.6076	23.52±0.182	24.45±0.0469	24.07±0.2024	24.81±0.8106	25.25±0.4104
**Spy0618**	25.18±0.3598	25.53±0.4133	26.06±0.2019	25.21±0.3951	25.42±0.7609	25.66±0.8603
**Spy0619**	27.97±0.0176	27.7±0.5231	28.27±0.1335	27.46±0.2034	27.09±0.3702	27.37±0.1753
**Spy0620**	31.7±0.3152	30.68±0.1392	31.31±0.2585	31.11±0.3041	30.86±0.6155	31.01±0.4822
**Spy0621**	29.6±0.2525	28.89±0.3518	29.07±0.6956	29±0.5179	28.7±0.3223	29.48±0.4335
**Spy0624**	24.22±0.1299	24.08±0.6731	24.11±0.6072	24.74±0.601	25.33±0.0719	24.76±0.4571
**Spy0626**	27.2±2.4527	27.8±1.1013	28.36±0.5225	28.23±0.8781	28.68±1.2854	28.66±0.5853
**Spy0627**	28.35±0.3959	27.79±0.2458	27.27±0.1913	29.41±0.2094	28.87±0.065	28.58±0.5697
**Spy0633**	31.8±0.2535	31.11±0.3083	31.27±0.0195	31.05±0.1245	31.16±0.1419	30.77±0.2726
**Spy0635**	32.82±0.2208	32.23±0.3297	32.48±0.1686	32.11±0.082	31.89±0.4491	31.94±0.3409
**Spy0639**	23.86±0.472	25.56±0.4274	26.89±0.3874	24.48±0.2741	24.52±0.0461	24.82±0.5258
**Spy0644**	26.55±0.7743	27.68±0.2631	24.59±0.8261	27.02±0.29	26.25±0.209	23.38±0.3742
**Spy0645**	24.23±0.5338	24.95±0.4446	25.28±0.0586	24.38±0.2207	24.49±0.0766	25.1±0.6718
**Spy0648**	30.14±0.4785	29.67±0.1436	30.65±0.0314	30.61±0.1035	30.87±0.159	29.9±0.5984
**Spy0649**	24.82±0.5578	24.45±1.2224	24.3±0.7017	24.8±0.6533	24.5±0.8692	24.43±0.8386
**Spy0655**	25.01±0.3775	25.26±0.4494	24.8±0.6998	24.75±0.932	25.17±0.2949	24.15±0.1117
**Spy0659**	28.3±0.9419	28.47±0.9042	28.87±0.3175	26.77±0.9504	25.48±0.6138	27.03±0.4378
**Spy0660**	24.43±0.1693	24.2±0.186	24.13±0.38	24.34±0.2951	23.84±0.392	24.29±0.5384
**Spy0661**	24.88±0.0004	25.84±0.2451	25.41±0.2513	25.71±0.1602	26.31±0.4312	27.17±0.4236
**Spy0662**	29.08±0.1043	29.25±0.3613	29.02±0.0338	29.23±0.3227	29.31±0.1483	29.68±0.429
**Spy0687**	24.52±0.0092	25.63±0.0909	26.05±0.0477	25.89±0.0274	25.71±0.0119	26.84±0.5419
**Spy0691**	27.71±0.4083	28.14±0.1453	27.98±0.0415	27.32±0.1187	27.26±0.021	28.08±0.1769
**Spy0694**	28.95±0.1901	29.75±0.153	30.05±0.167	29.37±0.1058	30±0.2861	30.63±0.1081
**Spy0696**	28.68±0.3286	29.92±0.1572	29.75±0.2176	31.06±0.4101	31.15±0.1166	30.5±0.2393
**Spy0698**	29.02±0.4391	29.05±0.1427	28.3±0.3996	29.73±0.6263	29.58±0.6553	28.99±0.0221

**Spy0699**	28.71±1.0305	28.7±0.5696	29.14±0.1881	28.91±0.7772	29.43±0.5985	29.66±0.2492
**Spy0701**	25.16±0.9107	25.37±0.1676	24.05±0.2653	24.74±0.7142	24.87±0.265	24.31±0.5346
**Spy0704**	24.24±0.3757	23.67±0.1441	24.11±0.1876	23.35±0.132	23.91±0.1199	23.54±0.3606
**Spy0708**	24.84±0.9825	23.96±0.4183	23.61±0.1084	24.15±0.0555	24.58±0.1245	23.59±0.506
**Spy0709**	26.3±1.3873	26.85±0.6604	27.56±0.2055	26.3±1.6295	26.81±0.7503	27.52±0.0648
**Spy0711**	24.88±0.3465	27.06±0.2811	27.65±0.4276	25.12±0.3051	25.82±0.8852	26.67±0.9806
**Spy0712**	26.83±0.4526	27.7±0.2145	27.56±0.5389	26.52±0.479	25.7±0.8254	26.96±0.3811
**Spy0713**	28.74±0.3267	27.31±0.667	26.79±0.3564	27.75±0.433	28.13±0.2996	27.19±0.1823
**Spy0714**	26.7±0.3184	26.44±0.2541	27.31±0.4092	26.36±0.4199	26.36±0.3543	26.14±0.1382
**Spy0715**	31.88±0.1816	31.84±0.0653	31.69±0.1821	31.08±0.0334	31.31±0.1579	31.36±0.2484
**Spy0721**	27.25±0.5571	27.13±0.1002	26.54±0.4376	27.68±0.2189	27.31±0.253	26.45±0.3234
**Spy0725**	26.98±0.1319	26.42±0.0287	26.32±0.7526	26.71±0.5542	26.33±0.5039	26.55±0.3872
**Spy0728**	23.66±1.1867	24.38±0.012	25.82±0.427	24.87±0.0399	24.35±0.5158	25.77±0.1405
**Spy0731**	26.88±0.138	29.84±0.5697	26.33±0.7154	24.64±1.1721	27.45±1.643	29.93±1.4038
**Spy0732**	28.48±0.5736	27.57±0.0094	25.51±0.7393	27.74±0.4554	27.49±0.4524	26.82±0.2206
**Spy0734**	27.95±0.0241	28.72±0.0275	29.4±0.0404	28.71±0.0455	29.07±0.1804	29.95±0.1229
**Spy0735**	28.83±0.3252	28.27±0.0945	27.76±0.0092	29.07±0.4008	28.78±0.2847	27.88±0.2587
**Spy0736**	25.36±1.8598	26.9±0.5854	26.82±0.0787	26.93±0.3616	27.01±0.4227	27.07±0.4439
**Spy0737**	24.07±0.0045	24.74±0.5129	24.54±0.1814	25.8±0.0582	25.9±0.3642	25.49±0.0392
**Spy0747**	28.48±0.3891	28.45±0.0358	28.56±0.0571	28.37±0.0056	28.25±0.0305	28.44±0.0839
**Spy0751**	28.19±0.1243	28.77±0.0279	28.72±0.0262	29.88±0.3393	30.12±0.347	30.58±0.0774
**Spy0752**	30.04±0.0283	30.39±0.036	30.52±0.0163	31.68±0.3158	31.95±0.3641	32.29±0.173
**Spy0753**	28.12±0.1725	28.6±0.0046	29.26±0.0258	30.15±0.0645	30.11±0.2104	31.05±0.1585
**Spy0755**	32.15±0.3803	32.03±0.1385	32.09±0.2097	33.99±0.0162	34.25±0.0357	34.35±0.108
**Spy0758**	27.7±0.3532	27.85±0.0293	27.75±0.0953	27.27±0.1217	27.41±0.0418	27.8±0.0619
**Spy0759**	24.74±0.5622	24.65±0.0535	24.18±0.3073	24.74±0.2489	24.47±0.7691	24.51±0.5604
**Spy0763**	27.93±0.0728	28.66±0.1133	28.89±0.2604	28.21±0.1077	28.39±0.2687	28.53±0.4152
**Spy0767**	23.66±0.0613	24.17±0.6398	23.73±0.7598	24.26±0.0386	24.12±0.3146	24.74±0.0347
**Spy0776**	23.92±0.1213	25.66±0.733	26.94±0.1643	24.41±0.4361	25.46±0.3783	26.38±0.109
**Spy0778**	24.32±0.1128	25.02±0.0573	24.74±0.2564	28.42±0.1874	28.37±0.0991	27.12±0.0085
**Spy0781**	23.81±0.2524	24.05±0.06	27.22±0.3337	29.34±0.0753	29.1±0.1241	29±0.0875
**Spy0790**	24.15±0.0643	23.69±0.2776	23.91±0.4606	28.6±0.5856	28.73±0.1285	27.39±0.3554
**Spy0792**	26.64±0.3108	26.17±0.5009	26.65±0.317	28.21±0.6031	28.17±0.4627	28.22±0.1332
**Spy0793**	28.4±0.1213	29.49±0.5163	30.06±0.252	30.22±0.159	30.22±0.392	30.53±0.2344
**Spy0795**	29.82±0.8892	30.13±0.1772	30.7±0.1194	29.81±0.1242	29.69±0.1651	29.7±0.273
**Spy0796**	32.51±1.8915	32.94±0.8623	33.14±0.9484	33.12±0.7148	33.19±1.0082	32.59±0.8944
**Spy0821**	27.31±0.5661	28.35±0.3558	28.54±0.2562	28.16±0.4466	28.19±0.1573	28.23±0.2226
**Spy0822**	26.13±0.17	27.33±0.0186	28.07±0.0083	27.21±0.0948	27.58±0.1068	28.04±0.1064
**Spy0823**	23.64±0.2798	24.38±0.0145	25.34±0.0423	23.63±0.1506	23.9±0.1194	24.93±0.1813
**Spy0824**	24.1±0.2902	25.78±0.2586	27.3±0.0533	25.72±0.0934	26.03±0.0043	27.29±0.0858
**Spy0825**	23.84±0.1626	23.68±0.1712	23.63±0.0921	23.87±0.2388	24.15±0.2158	23.8±0.2378
**Spy0826**	24.91±0.2667	25.73±0.4723	24.02±0.3498	24.37±0.366	24.22±0.3412	24.28±0.0505
**Spy0845**	27.42±0.0581	26.77±0.3686	26.77±0.2454	26.92±0.5043	26.93±0.3346	27.14±0.105

**Spy0847**	24.72±0.2191	25.12±0.2084	26.36±0.1113	24.99±0.0736	25.02±0.1197	26.41±0.1996
**Spy0848**	27.32±0.1653	27.23±0.4482	26.83±0.1926	26.8±0.5653	26.43±0.4632	26.85±0.3471
**Spy0851**	27.86±0.0882	29.28±0.3207	29.26±0.0646	30.65±0.066	30.55±0.0619	30.18±0.2305
**Spy0858**	23.84±0.1077	24.25±0.0403	26.32±0.1343	24.43±0.5731	24.27±0.4415	25.09±0.8195
**Spy0863**	25.34±0.1902	25.73±0.1354	25.51±0.5723	25.59±0	25.57±0.0002	25.45±0.329
**Spy0865**	23.54±0.0521	24.17±0.0774	24.14±0.3902	24.27±0.4363	24.62±0.373	24.83±0.5165
**Spy0867**	26.21±0.3127	26.16±0.2734	25.6±0.0082	27.38±0.0371	27.7±0.177	27.62±0.2961
**Spy0872**	29.33±0.0215	29.95±0.0223	30.38±0.0078	30.65±0.2277	30.83±0.4283	30.55±0.1223
**Spy0873**	29.46±0.0851	30.44±0.0413	31.58±0.1807	29.79±0.3365	29.78±0.1959	30.24±0.2288
**Spy0874**	26.72±0.2549	26.99±0.079	26.45±0.2777	26.29±0.087	26.3±0.1714	26.77±0.1361
**Spy0875**	25.17±0.472	23.84±0.1789	23.91±0.3752	23.52±0.4333	24.23±0.2932	24.01±0.2942
**Spy0876**	25.88±0.3757	25.51±0.4594	24.63±0.4073	26.54±0.0727	26.23±0.0375	24.85±0.0955
**Spy0878**	28.69±0.6108	29.06±0.6936	28.4±0.3302	30.15±0.4966	30.21±0.0153	29.3±0.0976
**Spy0885**	27±1.3348	28.05±0.3512	27.4±0.2289	27.86±0.1804	27.59±0.1773	27.47±0.0149
**Spy0893**	24.08±0.0586	24.81±0.6143	25.28±0.0525	26.28±0.4554	25.78±0.0068	25.34±0.0475
**Spy0910**	24.1±0.3957	23.49±0.0292	24.15±0.0384	23.76±0.5375	23.37±0.1962	24.02±0.1177
**Spy0911**	24.05±0.3848	23.96±0.1968	24.51±0.1369	23.63±0.05	23.95±0.1487	24.35±0.0714
**Spy0915**	27.06±0.1853	28.02±0.3246	28.48±0.2971	26.95±0.0847	27.07±0.1891	27.72±0.2006
**Spy0919**	25.84±0.6744	27.74±0.4747	28.83±0.1679	27.31±0.3177	27.83±0.2505	28.42±0.4827
**Spy0927**	27.48±0.699	27.11±0.0044	26.04±0.0559	27.82±0.0414	27.99±0.0507	27.54±0.3669
**Spy0928**	24.03±0.1734	24.75±0.7682	24.48±0.1832	24.12±0.4528	25.5±0.2888	26.32±0.3205
**Spy0931**	23.66±0.5176	24.47±0.3429	23.8±0.1661	27.63±0.1609	26.94±0.6805	25.58±1.0713
**Spy0938**	28.95±0.2606	29.39±0.0953	29.45±0.0517	30.46±0.0734	30.15±0.0545	29.88±0.1847
**Spy0941**	28.8±0.1145	29.1±0.1781	28.45±0.0239	28.37±0.042	27.24±1.6132	27.58±1.0901
**Spy0942**	29.12±0.2955	28.88±0.039	28.32±0.6032	28.13±0.0782	27.78±0.1532	27.7±0.0116
**Spy0946**	31.33±0.3642	30.42±0.5518	30.92±0.425	30.33±0.2438	30.49±0.3543	30.57±0.1967
**Spy0948**	24.11±0.248	23.64±0.3951	24.18±0.5333	25.66±0.0319	24.58±0.954	26.99±0.2148
**Spy0949**	29.14±0.6724	29.02±0.6505	29.02±0.2412	29.51±0.1292	29.64±0.0528	28.94±0.3714
**Spy0950**	25.52±0.2061	26.14±0.1281	27.24±0.0795	24.98±0.1998	24.73±0.0272	26.64±0.0443
**Spy0951**	25.04±0.2851	25±0.1924	25.24±0.0657	24.53±0.0674	24.15±0.7664	24.58±0.2427
**Spy0952**	26.33±0.1922	26.85±0.3751	26.38±0.2056	25.39±0.4693	25.63±0.2317	26.28±0.2868
**Spy0955**	26.15±0.2137	26.51±0.2028	24.96±0.6638	24.34±0.3742	24.72±0.1535	23.72±0.0156
**Spy0962**	27.52±0.8002	26.98±0.3761	27.42±0.6009	26.78±0.672	26.95±1.0068	26.98±1.2333
**Spy0971**	29.55±0.9846	29.97±0.1746	30.84±0.0851	30.85±0.0882	31.25±0.2619	31.78±0.1189
**Spy0972**	24.29±0.2464	23.99±0.5569	23.83±0.6736	25.29±0.366	25.38±0.8312	24.08±0.3481
**Spy0973**	31.64±1.0188	31.81±0.6773	31.95±0.2649	32.8±0.5121	32.85±0.2156	33.07±0.2464
**Spy0975**	25.63±0.3694	26.83±0.185	26.99±0.1599	26.76±0.2783	26.98±0.0589	27.54±0.1202
**Spy0977**	23.71±0.4081	24.83±0.1999	24.57±0.9289	23.68±0.4396	23.75±0.2221	23.8±0.1405
**Spy0981**	23.9±0.4903	24.83±0.7797	24.95±0.0485	25.36±1.0395	24.48±0.3955	24.01±0.0739
**Spy0985**	25.71±2.1161	23.63±0.0697	24.06±0.4827	23.96±0.1198	23.25±0.4477	24.13±0.3067
**Spy0986**	28.23±0.4179	28.6±0.2388	28.33±0.1203	27.58±0.2981	27.79±0.0259	28.27±0.25
**Spy0987**	24.11±0.6218	24.17±0.1999	23.81±0.0668	23.93±0.2008	23.96±0.2399	24.02±0.2315
**Spy0988**	31.5±0.0579	32±0.0034	33.11±0.0533	31.66±0.4394	31.65±0.1393	32.04±0.159

**Spy0989**	31.68±0.2685	31.62±0.4066	30.89±0.3974	31.38±0.1534	31.4±0.0092	30.47±0.2411
**Spy0991**	28.44±0.2108	28.54±0.4847	27.25±0.2746	28.06±0.4294	27.32±0.7204	27.61±0.3516
**Spy0992**	26.13±0.6001	27.99±0.2677	27.64±0.1213	27.4±0.0392	27.32±0.2476	28.01±0.254
**476_1104**[1]	26.68±0.5767	27.91±0.589	27.8±0.1068	29.92±0.1296	29.63±0.0969	29.59±0.3382
**Spy1050**	25.25±1.1501	24.47±0.157	24.4±0.2507	23.97±0.5096	24.92±0.6093	24.24±0.4218
**Spy1058**	28.29±0.4727	28.21±0.1847	23.88±0.3739	26.98±0.2084	26.16±0.3794	23.7±0.1347
**Spy1067**	24.9±0.2998	25.69±0.072	26.63±0.4295	28.75±0.0723	28.46±0.2177	29.03±0.0009
**Spy1069**	25.15±0.1933	25.53±0.1723	25.19±0.3524	24.38±0.2907	23.61±0.4621	24.06±0.3576
**Spy1070**	24.24±0.4027	23.61±0.4548	24.17±0.1562	23.86±0.8154	23.84±0.0123	23.96±0.3339
**Spy1071**	24.11±0.5035	24.74±0.0083	25.65±1.1843	25.87±0.3141	25.09±0.5852	26.74±0.1028
**Spy1073**	26.13±0.4817	26.77±0.0804	27.95±0.1823	26.65±0.0286	27.33±0.2563	27.97±0.2919
**Spy1075**	24.45±0.2947	24.4±0.3716	24.16±0.085	23.93±0.001	24.54±0.2256	25.61±0.0734
**Spy1076**	28.85±0.1032	28.57±0.6489	27.57±0.0565	26.57±0.4161	26.47±0.4323	24.78±0.2293
**Spy1077**	29.66±0.0748	29.88±0.4711	29.4±0.1425	28.52±0.2486	28.55±0.0515	28.33±0.1352
**Spy1088**	24.75±0.0162	25.4±0.0766	25.2±0.1021	23.87±0.0466	23.69±0.0718	25.02±0.2029
**Spy1095**	24.82±0.3969	25.95±0.039	25.47±0.2756	24.56±0.2016	25.06±0.0598	26.26±0.155
**Spy1098**	25.76±0.4951	25.57±0.2402	24.85±0.065	26.07±0.4725	25.8±0.1408	25.2±0.366
**Spy1103**	28.35±0.7904	28.05±0.3495	27.72±0.0976	28.46±0.28	28.58±0.617	28.17±0.3304
**Spy1106**	29.75±0.3993	27.19±3.3597	26.83±1.6395	25±1.3006	25.05±1.1392	24.1±0.1689
**Spy1107**	24.04±0.2576	24.46±0.3528	24.86±0.4913	23.86±0.119	24.19±0.1548	24.69±0.0813
**Spy1108**	25.37±0.1935	26.88±0.6568	27.2±0.4241	27.25±0.824	27.04±0.7045	27.36±0.3574
**Spy1116**	24.9±0.1629	24.68±0.3129	25.22±0.1086	23.81±0.1711	23.89±0.2477	24.3±0.474
**Spy1119**	33.56±0.1646	32.84±0.0343	32.34±0.1051	33.01±0.3347	32.97±0.2996	32.29±0.1885
**Spy1120**	27.58±0.2714	28.15±0.3221	29.63±0.1666	28.26±0.4148	28.36±0.0607	29.11±0.1941
**Spy1121**	26.35±2.0995	28.48±0.1861	28.65±0.1936	29.23±0.2937	30.26±0.0685	28.89±0.0954
**Spy1123**	26.48±1.2921	27.31±0.3368	27.37±0.2583	27.49±0.6173	27.69±0.7498	28.35±0.2483
**Spy1124**	25.65±0.379	26.39±0.3357	26.58±0.098	26.13±0.5447	26.03±0.5414	26.51±0.3267
**Spy1132**	27.25±0.1589	27.79±0.1368	28.71±0.1187	27.7±0.1097	27.94±0.0392	28.43±0.2691
**Spy1133**	28.36±0.3646	28.7±0.2811	24.51±0.7183	26.95±0.1412	26.35±0.0919	23.93±0.2702
**Spy1136**	25.76±1.0254	25.11±1.0679	25.62±0.3481	24.74±1.5437	24.72±1.3811	25.1±0.9608
**Spy1145**	27.83±0.1333	27.96±0.026	26.74±0.097	29.61±0.0536	29.6±0.1669	28.21±0.9606
**Spy1154**	26.02±0.087	25.67±0.1012	26.57±0.1202	26.02±0.3227	26.11±0.0311	26.6±0.0305
**Spy1155**	26.15±0.5018	27.02±0.1653	27.39±0.0366	25.7±0.3745	26.25±0.3031	27.39±0.2257
**Spy1158**	25.32±0.6419	25.06±0.0045	24.44±0.5699	25.55±0.5195	25.37±0.265	25.45±0.0144
**Spy1163**	26.09±0.207	26.83±0.5714	24.28±0.1748	25.68±0.9527	25.95±0.2129	24.47±0.0451
**Spy1164**	31.9±0.361	31.74±0.2724	31±0.0639	31.73±0.0026	31.76±0.0266	30.82±0.2194
**Spy1165**	26.74±0.0067	26.5±0.2308	26.06±0.0068	26.59±0.1468	26.44±0.0737	26.36±0.1724
**Spy1167**	26.68±1.8046	26.77±0.5693	28.77±1.1446	23.78±0.1513	26.44±0.5126	24.8±0.903
**Spy1219**	26.58±0.3268	25.68±0.6375	24.76±0.4362	25.04±0.176	25.22±0.1665	24.25±0.1155
**Spy1221**	25.35±0.1979	26.03±0.2764	26.48±0.018	25.74±0.3133	25.19±0.7177	24.88±0.4212
**Spy1223**	32.97±0.1107	32.34±0.8351	31.01±0.7913	31.96±0.531	32.5±0.2537	31.24±0.5815
**Spy1226**	24.14±0.2464	24.45±0.0722	24.2±0.3094	24.12±0.2433	23.69±0.1797	24.29±0.0521
**Spy1229**	24.9±0.9896	25.33±0.7154	25.25±0.9966	24.17±0.8576	24.58±0.0544	23.86±0.132

**Spy1235**	25.76±1.6645	28.21±0.4374	28.18±0.0082	30.64±0.1233	30.73±0.1433	30.17±0.2797
**Spy1236**	25.43±0.8732	25.28±0.5555	23.96±0.0135	26.69±0.0568	26.59±0.2573	25.24±0.5269
**Spy1240**	28.22±0.6152	28.81±0.3524	29.23±0.111	29.13±0.2154	29.61±0.0589	30.2±0.1434
**Spy1242**	27.39±0.4402	28.05±0.0052	27.65±0.3753	27.16±0.1555	27.23±0.1779	27.65±0.4451
**Spy1243**	27.94±1.923	28.55±0.9655	28.41±0.1407	28.93±0.7832	29.09±0.642	28.49±0.477
**Spy1244**	31.28±0.998	30.93±0.4647	31.28±0.1617	31.39±0.3903	31.36±0.3361	30.86±0.2575
**Spy1247**	24.83±0.6903	25.78±1.3675	25.79±0.1124	25.6±0.223	26.05±0.3233	26.48±0.0245
**Spy1249**	29.12±0.0241	29.55±0.006	30.25±0.2162	29.55±0.009	29.66±0.2049	30.14±0.1779
**Spy1250**	25.68±0.1238	26.2±0.0919	27.54±0.0065	25.95±0.1579	26.03±0.01	26.74±0.3039
**Spy1255**	25.3±1.0241	27.65±0.0546	28.52±0.3035	27.51±0.2201	27.66±0.0268	28.62±0.187
**Spy1257**	23.7±0.4899	25.07±1.4904	26.79±0.1808	25.25±1.3043	25.8±1.0415	26.44±0.3353
**Spy1259**	29.97±0.491	31.51±0.2615	31.05±0.654	30.01±0.501	30.13±0.2693	30.63±0.3169
**Spy1262**	25.01±1.332	25.85±0.1231	25.66±0.4918	24.86±1.2341	25.15±0.8022	25.04±0.3586
**Spy1269**	23.97±0.2088	24.31±0.1913	24.78±0.0476	24.41±0.5469	24.97±0.4201	26.67±0.3339
**Spy1270**	23.87±0.0549	26.31±1.3268	26.22±0.8022	29.86±1.0409	29.64±0.7949	28.64±0.2903
**Spy1271**	23.82±0.1498	24.07±0.2446	24.9±0.2399	28.55±0.2796	28.69±0.6575	27.49±0.2869
**Spy1273**	26.07±1.1999	28.53±0.3566	27.9±0.7401	31.88±0.6747	31.94±0.3088	31.15±0.5607
**Spy1275**	25.23±0.9745	27.34±1.5834	29.76±0.0143	32.73±0.0131	32.52±0.2254	31.87±0.4313
**Spy1278**	24.25±0.151	24.83±0.9024	25.86±0.4968	26.36±0.4209	26.19±0.2	26.58±0.2264
**Spy1288**	25.29±0.4179	24.04±0.1696	23.67±0.067	23.86±0.2076	23.6±0.1556	24.2±0.4628
**Spy1292**	27.09±0.1802	27.89±0.8117	28.85±0.3703	28.14±0.5554	27.99±0.3574	28.49±0.2305
**Spy1295**	27.92±0.0846	27.02±0.1575	27.25±0.0095	26.93±0.0078	26.55±0.5016	25.85±0.3322
**Spy1320**	25.41±0.3011	26.25±0.5372	26.45±0.3514	25.29±0.4712	26.11±0.2219	26.37±0.1411
**Spy1325**	29.2±0.5247	29.72±0.1523	30.81±0.1273	30.76±0.1992	30.57±0.2879	31.05±0.1497
**Spy1329**	29.01±0.5105	29.29±0.3709	29.25±0.0705	30.92±0.138	30.9±0.1543	30.93±0.2813
**Spy1331**	25.77±0.817	26.17±0.0072	24.91±0.4016	25.75±0.0915	26.15±0.2246	25.44±0.6772
**Spy1335**	27.44±0.4204	28.06±0.2142	27.8±0.0604	27.23±0.2846	27.25±0.02	27.79±0.1926
**Spy1336**	26.2±0.39	27.53±0.3029	29.12±0.2561	28.08±0.2413	28.19±0.2399	28.88±0.1663
**Spy1337**	25.02±0.158	24.04±0.11	23.97±0.2595	24.14±0.1495	23.73±0.5081	24.01±0.1185
**Spy1338**	23.79±0.0319	23.9±0.0543	23.73±0.2904	23.72±0.2255	23.31±0.2569	24.81±0.1061
**Spy1340**	29.61±0.3974	28.26±0.2813	28.39±0.083	28.45±0.4013	28.6±0.1024	27.43±0.1911
**Spy1341**	24.04±0.4633	23.72±0.0274	24.12±0.2558	23.87±0.3003	23.43±0.0636	24.56±0.4137
**Spy1342**	27.6±0.2655	27.9±0.275	27.64±0.0048	27.31±0.1728	27.34±0.078	27.72±0.1741
**Spy1344**	26.17±0.064	25.99±0.0624	25.97±0.1169	26.92±0.2733	26.7±0.6335	26.61±0.045
**Spy1347**	23.47±0.3127	23.8±0.683	24.59±0.079	23.85±0.1684	24.79±0.3466	24.66±0.0893
**Spy1350**	27.7±0.0418	27.52±0.7547	26.87±0.2114	26.8±0.481	26.15±0.7153	24.75±0.5822
**Spy1351**	24.27±0.5041	24.21±0.4592	23.7±0.2894	23.91±0.304	23.99±0.3974	23.78±0.0802
**Spy1352**	28.93±0.7022	28.46±0.2836	27.79±0.3846	27.17±0.8196	27.13±0.605	26.58±0.2166
**Spy1355**	26.34±0.3133	24.54±0.9562	23.6±0.0485	24.33±0.0246	24.04±0.2727	24.37±0.3876
**Spy1356**	31.44±0.29	31.05±0.1122	30.4±0.3372	32.4±0.2986	32.31±0.2387	32.48±0.3088
**Spy1357**	24.02±0.2536	24±0.3754	25.92±0.1663	24.85±0.4217	24.93±0.2509	26.25±0.1086
**Spy1358**	26.51±0.3609	27.38±0.0735	28.89±0.0478	27.05±0.2735	27.49±0.0359	29±0.0827
**Spy1360**	29.56±0.8208	29.1±0.0141	28.86±0.0226	30.35±0.3589	30.04±0.2981	29.39±0.0918

**Spy1362**	24.02±0.0071	24.78±0.6662	23.98±0.1023	24.34±0.6608	24.14±0.0558	24.79±0.7784
**Spy1364**	23.33±0.6695	26.12±2.3323	25.62±2.1511	23.26±0.1165	23.72±0.0403	24.35±0.6567
**Spy1372**	24.95±0.3812	25.02±0.0771	23.68±0.3006	24.24±0.8559	24.5±0.3034	24.12±0.428
**Spy1375**	29.74±0.238	30.07±0.7615	30.76±0.0062	30.16±0.6123	30.09±0.3952	30±0.0497
**Spy1376**	24.25±0.093	24.66±0.2799	24.52±0.1427	24.47±0.064	24.35±0.374	24.43±0.3342
**Spy1382**	23.61±0.1168	24.67±1.1656	23.96±0.5397	23.46±0.5823	23.8±0.5221	24.34±0.1536
**Spy1383**	29.1±0.6596	28.12±0.216	28.26±0.5012	28.5±0.4951	28.34±0.8213	28.61±0.4925
**Spy1384**	27.88±0.0514	28.58±0.0624	29.18±0.0419	28.16±0.3159	28.45±0.0788	29.23±0.3105
**Spy1385**	25.85±0.6131	27.09±0.2835	27.49±0.0324	26.91±0.1795	27.14±0.3736	27.57±0.459
**Spy1387**	24.85±0.4697	26.37±0.227	26.37±0.0081	27.15±0.2141	27.44±0.402	28.02±0.0612
**Spy1388**	25.86±0.3458	26.03±0.0152	26.14±0.0744	27.89±0.1496	27.87±0.3476	27.05±0.046
**Spy1390**	24.96±0.3009	25.46±0.2182	24.61±0.1731	24.5±0.3569	23.8±0.2003	24.17±0.3636
**Spy1393**	24.06±0.1541	23.71±0.161	24.47±0.5006	23.66±0.0146	23.89±0.1455	24.4±0.7515
**Spy1397**	23.63±0.2008	23.88±0.1274	24.37±0.0101	24.9±0.3216	23.65±0.1806	24.1±0.0713
**Spy1400**	24.32±0.2278	23.65±0.4117	25.19±0.3108	27.24±0.0882	27.4±0.0591	26.86±0.3549
**Spy1408**	25.71±0.3352	26.06±0.4263	26.06±0.2329	25.86±0.1214	25.41±0.9384	25.48±0.6586
**Spy1409**	28.77±0.0273	29.2±0.0494	29.86±0.1761	28.94±0.0758	29.3±0.1342	29.9±0.0996
**Spy1411**	27.45±0.2397	26.54±0.3183	26.03±0.0947	26.7±0.0764	26.34±0.3866	24.7±1.1068
**Spy1412**	29.44±0.3916	29.29±0.0156	28.79±0.2057	28.7±0.3861	28.64±0.288	28.42±0.2642
**Spy1424**	25.21±0.1587	25.22±0.7224	25.67±0.7772	25.11±0.2164	24.33±0.6491	24.16±0.8641
**Spy1440**	24.43±0.289	24.62±0.5591	24.97±0.7491	24.44±0.1489	23.4±0.3045	24.52±0.8344
**Spy1451**	25.15±0.4761	24.49±0.3484	23.62±0.3333	23.63±0.0563	24.31±0.1903	23.65±0.0536
**Spy1466**	25.63±0.55	25.52±0.0008	25.13±0.3515	24.85±0.0811	24.74±0.0654	24.63±0.2903
**Spy1469**	24.79±0.0057	25.37±0.3587	25.34±0.2976	24.74±0.4546	24.2±0.1911	25.15±0.2395
**Spy1471**	24.03±0.018	24.4±0.5733	24.88±0.0471	23.26±0.1946	23.85±0.1131	24.4±0.3513
**Spy1479**	30.6±0.1244	31.02±0.3702	30.4±0.0612	29.87±0.0658	29.94±0.0326	29.88±0.2263
**Spy1481**	24.76±1.1939	27.75±0.3116	27.14±0.2937	24.4±0.766	24.71±0.0169	24.54±1.2472
**Spy1483**	28.34±0.3347	29.36±0.8207	30.11±0.2948	29.07±0.9294	28.73±0.5524	29.23±0.2128
**Spy1484**	26.6±0.2272	26.93±0.3951	27.24±0.0383	27.08±0.5738	26.88±0.4815	27.11±0.1538
**Spy1485**	26.53±0.3546	27.33±0.3342	27.74±0.0049	26.98±0.6163	27.01±0.4296	27.12±0.3873
**Spy1486**	25.92±0.6115	26.71±0.3702	25.7±0.8635	25.16±0.4852	25.43±0.0601	26.44±0.4892
**Spy1487**	28.04±0.0297	27.62±0.3216	27.26±0.1567	27.19±0.9114	27.67±0.28	27.42±0.4033
**Spy1488**	26.82±1.5146	27.52±0.3168	27.19±0.0992	27.87±0.1412	27.74±0.1086	26.99±0.0254
**Spy1489**	29.03±0.2724	29.4±0.0594	29.25±0.1414	29.19±0.1048	29.16±0.0695	29.55±0.192
**Spy1490**	27.53±0.1395	27.63±0.1067	28.54±0.0325	27.17±0.375	26.82±0.0111	28.4±0.329
**Spy1491**	25.74±0.1253	27.05±0.1686	28.11±0.0461	26.35±0.6156	26.69±0.0729	27.71±0.5871
**Spy1492**	27.59±0.1414	27.84±0.2841	27.8±0.0905	27.89±0.2956	27.59±0.0368	27.66±0.2998
**Spy1496**	25.76±0.6125	26.31±0.3732	27.41±0.4398	26.01±0.0466	26.45±0.0807	26.16±0.2536
**Spy1497**	29.02±0.1312	29.85±0.0563	29.92±0.1448	28.75±0.0259	29.11±0.2349	29.36±0.0317
**Spy1498**	33.6±0.3301	34.27±0.3007	34.83±0.1481	34.15±0.0826	34.04±0.0501	34.01±0.1429
**Spy1499**	30.63±1.1042	30.53±0.112	30.22±0.0073	30.89±0.4725	30.78±0.4708	30.23±0.3761
**Spy1503**	24.05±0.2397	23.78±0.5747	25.19±0.0419	23.72±0.1002	24.17±0.0016	24.76±1.0149
**Spy1506**	25.14±0.4281	26.15±0.0955	26.04±0.0266	26.15±0.046	26.14±0.0008	26.57±0.1806

**Spy1507**	25.76±0.0265	26.51±0.2318	26.57±0.1828	26.24±0.1954	26.5±0.0409	26.99±0.1508
**Spy1508**	24.86±0.9392	24.48±0.2504	23.97±0.2265	25.26±0.2767	24.68±0.5545	24.41±0.682
**Spy1512**	27.52±0.0996	28.28±0.4817	28.56±0.0806	26.82±0.3104	26.86±0.0962	27.81±0.2652
**Spy1513**	24.21±0.3464	25.84±0.8879	27.37±0.1492	26.14±0.7748	26.47±0.289	27.92±0.2908
**Spy1514**	27.36±0.3776	28.38±0.4516	28.87±0.07	27.88±0.2047	27.91±0.1652	28.91±0.0462
**Spy1515**	24.04±0.8418	24.55±0.5554	24.73±0.9727	24.2±0.3044	24.2±0.3539	23.68±0.3828
**Spy1534**	29.85±0.2315	30.45±0.2154	30.2±0.1106	29.51±0.0134	29.59±0.1279	30.05±0.2968
**Spy1538**	24.09±0.1638	24.71±0.4935	25.04±0.1422	25.12±0.727	24.83±0.8778	25.33±0.3998
**Spy1546**	24.02±1.0019	25.38±0.2716	25.99±0.8902	25.46±0.9751	25.76±0.4845	26.05±0.4538
**Spy1547**	27.86±0.1326	28.66±0.3285	28.86±0.5511	28.65±0.4107	28.69±0.2864	28.41±0.1016
**Spy1550**	27.89±0.9233	28.55±0.0421	27.94±0.0622	28.23±0.3929	28.15±0.5838	28.71±0.256
**Spy1551**	24.18±0.2734	24.17±0.2365	24.1±0.1281	23.41±0.176	23.78±0.3682	23.89±0.1294
**Spy1552**	24.19±0.3958	24.42±0.5679	23.61±0.1618	23.45±0.1501	23.89±0.1504	24.11±0.2295
**Spy1553**	30.1±0.3209	29.07±0.6914	29.17±0.3936	29.12±0.2931	28.67±0.0637	28.19±1.0151
**Spy1554**	29.43±0.3861	28.33±0.6311	26.97±0.2023	27.54±0.9148	27.15±0.5328	26.94±0.1361
**Spy1555**	30.41±0.6141	30.46±0.2565	29.66±0.4382	30.32±0.0172	29.62±0.0601	28.4±1.7517
**Spy1559**	28.33±0.1894	29.56±0.5268	29.16±0.021	30.96±0.2935	30.68±0.3701	30±0.1146
**Spy1565**	26.15±0.5878	26.61±0.0348	24.25±0.4834	24.55±0.3121	24.08±0.1726	24±0.2277
**Spy1569**	28.12±0.1418	28.98±0.0434	29.08±0.175	28.77±0.2561	29.16±0.0232	30.34±0.5131
**Spy1571**	25.06±0.7126	23.74±0.0773	26.38±0.3169	25.04±0.2861	25.98±0.0931	25.89±0.2021
**Spy1576**	24.99±0.3177	24.02±0.758	23.75±0.2033	23.79±0.366	24.12±0.3011	23.99±0.4748
**Spy1577**	23.57±0.7517	25.99±0.3545	27.76±0.1562	26.3±1.2369	25.9±1.9202	27.14±0.3663
**Spy1579**	26.34±0.3936	24.85±0.1395	24.47±0.3545	24.35±0.0799	24.06±0.1326	24.08±0.1985
**Spy1580**	27.86±1.3897	28.18±0.443	29.01±0.2799	28.54±0.7946	28.26±0.7116	28.11±0.4552
**Spy1583**	28.68±0.925	27.45±0.3908	26.63±0.0127	26.74±0.3933	26.49±0.6916	24.64±0.982
**Spy1585**	27.33±0.4491	28.21±0.2798	27.43±0.1942	29.53±0.0937	29.22±0.1577	28.13±0.1203
**Spy1587**	24.74±0.3483	26.53±0.1558	26.5±0.1053	28.89±0.1821	28.8±0.0631	27.3±0.5289
**Spy1591**	24.13±0.2373	24.66±0.2319	25.26±0.1068	23.97±0.5128	24.73±0.3571	25.9±0.3807
**Spy1595**	27.43±0.4541	27.4±0.1841	27.82±0.028	27.57±0.1051	27.42±0.1937	27.78±0.0886
**Spy1596**	31.39±0.5882	30.39±0.2169	29.67±0.623	30.11±0.5953	29.9±0.6866	29.22±0.1828
**Spy1597**	28.58±0.4953	27.96±0.1923	27.07±0.5501	25.37±0.3166	25.37±0.0992	24.62±0.1674
**Spy1599**	34.11±0.2512	33.85±0.5687	33.51±0.2672	34.04±0.3047	33.98±0.1471	33.12±0.2003
**Spy1600**	26.82±0.0473	23.53±0.1268	23.81±0.1431	23.53±0.0579	24.37±0.3221	24.54±0.3189
**Spy1601**	29.35±0.3031	29.75±0.0324	29.91±0.0025	28.48±0.0908	28.07±0.0357	28.81±0.087
**Spy1602**	27.92±0.1871	28.46±0.0481	28.6±0.0619	27.97±0.0276	28.03±0.0657	28.46±0.2559
**Spy1603**	24.85±0.2521	25.3±0.0862	26.85±0.0595	25.42±0.0951	25.83±0.6168	26.19±0.9132
**Spy1606**	31.71±0.2864	30.98±0.167	30.99±0.0446	31.1±0.0945	30.87±0.6676	30.33±1.142
**Spy1607**	29.93±0.619	30.4±0.0697	29.97±0.1261	29.83±0.0669	29.32±0.1803	29.08±0.0392
**Spy1608**	25.04±0.1484	23.91±0.0481	23.81±0.435	23.57±0.3443	23.89±0.2337	24.01±0.083
**Spy1610**	25.8±0.3136	25.37±0.2549	23.97±0.64	25.38±0.4019	24.92±0.474	24.73±0.0909
**Spy1611**	28.89±0.3256	27.63±0.4571	26.83±0.2292	27.4±0.4064	27.36±0.694	26.61±0.2123
**Spy1612**	31.71±0.6946	31.59±0.2771	31.78±0.0515	31.9±0.2288	32.13±0.3139	31.46±0.0826
**Spy1614**	24.1±0.1691	24.54±0.445	24.4±0.0064	23.77±0.1262	23.71±0.1955	24.21±0.2819

**Spy1622**	25.5±0.308	25.26±0.2017	23.58±0.0413	24.74±0.4343	24.19±0.1641	24.26±0.2025
**Spy1623**	27.25±1.2908	27.72±0.1488	26.64±0.1099	27.23±0.2863	27.43±0.2416	26.49±0.0712
**Spy1635**	23.6±0.6519	24.07±0.4406	24.82±0.3124	27.23±0.1024	27.29±0.2073	25.87±0.2325
**Spy1646**	31.27±0.8466	30.89±0.8278	31.36±0.2885	31.18±0.2081	31.4±0.1396	30.78±0.4083
**Spy1647**	29.37±0.2495	29.35±0.3528	30.29±0.4032	28.93±0.4147	28.68±0.1329	29.66±0.4252
**Spy1650**	24.07±0.1495	25.73±0.3391	26.85±0.154	25.46±0.871	25.49±0.9401	27.09±0.1648
**Spy1652**	25.05±1.4471	26.02±0.8346	26.08±0.5003	25.66±1.511	25.77±0.7717	25.84±0.4334
**Spy1659**	23.88±0.0963	25.02±0.3365	25.22±0.0905	24.33±0.0574	24.72±0.168	25.36±0.2936
**Spy1660**	27.95±0.2653	27.8±0.3249	27.47±0.2747	27.92±0.2678	27.42±0.1325	27.04±0.4712
**Spy1666**	30.75±1.1666	30.23±1.1263	30.85±0.1475	30.53±0.8252	30.6±0.4804	29.96±0.6853
**Spy1669**	26.38±0.0332	26.96±0.1181	28.23±0.0723	27.33±0.2214	27.08±0.3077	28.14±0.1594
**Spy1670**	23.81±0.0008	23.6±0.1333	23.46±0.2658	23.79±0.3024	23.66±0.0055	23.75±0.0968
**Spy1672**	24.29±0.2621	25.03±0.0764	25.3±0.1184	24.79±0.0354	24.86±0.2746	25.79±0.1637
**Spy1673**	30.25±0.8763	30.38±0.3616	30.19±0.0364	30.53±0.1028	30.67±0.0985	29.89±0.3124
**Spy1674**	24.86±0.5231	25.34±0.0878	25.02±0.0847	24.24±0.3341	24.41±0.1856	24.55±0.5649
**Spy1678**	23.77±0.2175	25.12±0.2161	25.08±0.0939	27.03±0.073	27.11±0.2759	26.39±0.1032
**Spy1682**	25.61±0.532	26.54±0.0574	25.88±0.2154	25.9±0.2042	26.17±0.3266	26.3±0.1102
**Spy1686**	24.21±0.205	24.38±0.0079	24.55±0.2426	24.27±0.136	23.93±0.244	24.07±0.3421
**Spy1704**	25.07±0.3399	24.57±0.5431	23.19±0.5103	23.57±0.2337	23.42±0.1097	24.08±0.2675
**Spy1709**	25.28±0.6453	24.31±0.6723	24.06±0.0913	24.29±0.3112	24.1±0.3328	24.01±0.1695
**Spy1714**	28.53±1.3242	26.71±1.4447	27.04±0.3307	23.42±0.4368	23.83±0.0079	24.35±0.3209
**Spy1715**	28.32±1.7714	25.86±1.8419	24.38±0.0232	23.55±0.3811	23.62±0.3638	24.26±0.0657
**Spy1719**	34.4±0.8921	34.1±0.5605	30.97±0.6557	27.94±0.2408	25.24±1.5727	21.47±0.6536
**Spy1727**	24.44±0.4544	24.91±0.0959	23.75±0.1706	24.91±0.6432	25.59±0.6312	26.18±0.267
**Spy1728**	29.17±0.1399	28.29±0.4335	24.66±0.3644	28.94±0.1188	28.87±0.4671	26.37±0.1614
**Spy1729**	28.63±0.8328	24.2±0.8413	23.85±0.3214	24.92±0.9474	24.69±0.5975	24.37±0.0252
**Spy1732**	32.36±0.3169	32.89±0.4507	28.97±0.272	32.76±0.0101	32.69±0.1926	30.35±0.3991
**Spy1734**	24.22±0.24	27.12±0.0267	27.17±0.1053	30.71±0.2019	31.19±0.0196	29.86±0.3494
**Spy1735**	25.37±1.2349	28.77±0.6068	28.11±0.4601	29.97±0.5823	29.81±0.2158	29.61±0.063
**Spy1741**	23.35±0.6945	25.37±0.3221	24.24±0.6495	26.14±0.5543	25.1±1.0036	25.81±0.5847
**Spy1742**	24.1±0.4123	24.73±0.299	24.46±0.1661	25.31±0.4461	24.78±0.4924	24.25±0.0961
**Spy1752**	26.55±0.0916	25.43±0.3855	25.9±0.0099	25.07±0.2312	25.33±0.1388	24.92±0.3203
**Spy1753**	24.68±0.3504	24.75±0.1286	24.44±0.4078	24.11±0.4662	23.79±0.6586	24.47±0.1149
**Spy1755**	25.22±0.5725	26.16±1.2753	25.75±0.591	25.75±1.7437	26.09±1.4436	24.43±0.0238
**Spy1761**	33.23±0.4167	33.61±0.2979	34.54±0.0705	33.1±0.3521	32.85±0.0889	34.18±0.0205
**Spy1762**	31.97±0.7075	31.85±0.3636	31.68±0.399	31.94±0.2746	32.41±0.2504	31.53±0.2912
**Spy1763**	27.23±0.0506	27.79±0.0918	26.88±0.3798	26.03±0.5019	26.54±0.1302	26.28±0.1785
**Spy1768**	29.99±0.7048	30.21±0.0381	31.3±0.0003	32.25±0.2314	32.4±0.2368	32.61±0.1639
**Spy1769**	28.83±0.4258	29.5±0.3736	30.44±0.0954	30.61±0.5821	30.79±0.4994	31.42±0.005
**Spy1780**	30.7±0.0223	31.05±0.1782	31.64±0.148	30.6±0.0386	30.51±0.1279	30.98±0.0122
**Spy1781**	30.69±0.0522	31.23±0.1296	31.77±0.2648	30.81±0.1224	31.01±0.1362	31.05±0.2522
**Spy1782**	29.28±0.4501	28.89±0.4965	28.57±0.013	29.13±0.4729	29.25±0.4181	28.86±0.5815
**Spy1783**	24.05±0.1616	24±0.2907	24.04±0.1825	24.59±0.5412	24.78±0.5716	24.01±0.9647

**Spy1791**	25.27±0.3278	25.81±0.3294	25.46±0.5677	24.23±0.5563	24.64±0.1299	25.28±0.4453
**Spy1793**	24.27±0.0264	25.56±0.1665	24.19±0.4499	23.56±0.0381	24.17±0.1528	24.55±0.02
**Spy1797**	27.34±0.8477	27.3±0.1572	27.65±0.2887	28.15±0.2186	28.64±0.1321	27.56±0.4034
**Spy1799**	26.83±0.4025	27.96±0.2251	27.88±0.1547	27.25±0.0144	27.31±0.1307	27.43±0.135
**Spy1808**	28.51±0.4746	29.53±0.1227	30.45±0.0533	29.4±0.1186	29.69±0.1142	30.64±0.3458
**Spy1813**	26.18±0.1099	27.07±0.3963	28.44±0.154	26.43±1.0821	26.71±0.424	27.93±0.1216
**Spy1814**	27.44±0.0563	27.6±0.3063	28.26±0.1334	27.81±0.0297	27.77±0.0175	28.04±0.1601
**Spy1816**	29.47±0.3563	28.21±0.3138	29.03±0.1012	28.08±0.1525	27.31±1.1183	28.5±1.1324
**Spy1831**	29.47±0.192	29.99±0.496	30.94±0.595	29.13±0.3553	28.81±0.0712	29.61±0.2514
**Spy1836**	28.73±0.208	28.54±0.5218	29.35±0.3022	28.58±0.2812	28.67±0.0086	28.58±0.1189
**Spy1837**	24.07±0.4517	24.23±0.0985	24.04±0.3971	23.85±0.2638	24.5±0.0106	24.46±0.4644
**Spy1838**	25.79±0.4386	26.71±0.1857	26.76±0.1228	26.06±0.0898	25.47±0.9003	26.86±0.3165
**Spy1840**	24.95±0.2333	27.08±0.1996	27.39±0.0851	24.53±0.1	25.19±0.4931	27.31±0.6732
**Spy1842**	25.28±0.1352	25.76±0.2912	25.33±0.2092	23.88±0.2841	23.74±0.5226	23.55±0.4418
**Spy1848**	28.88±0.2947	28.73±0.1529	28.69±0.2486	27.19±0.0042	27.06±0.1736	27.11±0.1743
**Spy1857**	30.49±0.1502	30.68±0.0904	31.13±0.0789	29.81±0.0125	29.95±0.0729	30.45±0.2533
**Spy1858**	24.22±0.1515	24.45±0.2221	25.83±0.0928	24.05±0.0203	24.22±0.0826	25.34±0.2246
**Spy1861**	27.79±0.1748	28.56±0.0409	29.25±0.1062	27.41±0.2093	27.92±0.0715	28.76±0.3429
**Spy1865**	30.41±0.4694	31.12±0.4405	25.32±0.858	30.65±0.0829	30.49±0.0348	27.48±0.0282
**Spy1866**	23.79±0.5866	25.75±0.4119	26.02±0.1306	25.01±0.2125	24.68±0.8008	25.5±0.5938

[1] Protein not annotated in M5005 genome, annotation refers to *S. pyogenes* M1 strain 476
