# Supplementary material for: Global Analysis and Comparison of the Transcriptomes and Proteomes of Group A Streptococcus Biofilms
Source: mSystems. 2016 Dec 6;1(6):e00149-16. doi: 10.1128/mSystems.00149-16 (PMC5141267; doi:10.1128/mSystems.00149-16)
Supplement: Table S2 [file sys006162066st9.pdf]

Table S2. Mean LFQ intensities for extracellular protein fraction

	LFQ Intensity (Log_2 Value ± Standard Deviation)					
**M5005 Locus**	**Early Log**	**Late Log**	**Late Stationary**	**Early Biofilm**	**Maturing Biofilm**	**Late Biofilm**
**Spy0002**	29.39±0.221	29.61±0.1646	32.07±0.013	29.96±0.2462	30.4±0.0187	32.24±0.1418
**Spy0004**	30.92±0.2639	29.76±0.5902	25.91±0.6758	29.03±0.0709	28.43±0.0823	26.21±0.1957
**Spy0012**	29.6±0.2241	29.34±0.47	26.52±0.0706	29.92±0.1951	30±0.2997	28.31±1.2875
**Spy0013**	28.19±0.6601	28.83±0.0822	29.64±0.0316	29.32±0.1753	30.03±0.2756	29.85±0.1972
**Spy0017**	27.28±0.8402	26.2±0.5516	26.19±0.6454	25.47±0.211	25.77±0.2924	25.87±0.5608
**Spy0018**	29.76±0.1308	30.11±0.0845	28.67±0.1613	29.69±0.1168	30.04±0.2629	29.8±0.4656
**Spy0020**	26.05±0.923	26.18±0.0675	25.27±0.4319	25.74±0.3209	24.84±0.1903	25.84±0.2619
**Spy0033**	25.78±0.7268	25.61±0.4212	26.44±0.0211	25.91±0.6542	25.36±0.0765	25.39±0.0455
**Spy0039**	30.09±1.0581	31.95±0.3213	29.33±0.6035	30.27±0.0887	29.49±1.1028	28.38±1.6123
**Spy0040**	25.66±0.1538	27.61±0.3098	26.25±0.0428	27.95±0.1868	28.31±0.0687	25.64±0.6072
**Spy0043**	33.01±0.096	32.85±0.2279	33±1.1127	33.05±0.197	33.17±0.4733	33.63±0.6913
**Spy0044**	31.63±0.0725	31.73±0.0852	32.69±0.5244	31.37±0.285	31.94±0.178	33.11±0.0008
**Spy0045**	32.93±0.6159	32.71±0.0746	32.71±0.2719	32.12±0.1451	32.54±0.2068	32.89±0.1143
**Spy0046**	31.57±0.2692	30.96±0.3941	33.16±0.1519	31.31±0.1383	31.59±0.0004	33.03±0.3616
**Spy0047**	33.87±0.1645	33.91±0.0677	35.48±0.0235	34.13±0.1766	34.48±0.1389	35.57±0.0093
**Spy0048**	30.44±0.6532	30.78±1.2421	31.06±1.7433	30.34±0.4195	30.97±0.3136	32.71±0.2194
**Spy0049**	32.19±0.1001	31.77±0.0435	33.51±0.176	32.28±0.1512	32.55±0.1117	33.89±0.1738
**Spy0050**	32.92±0.0583	32.67±0.2179	30.55±0.8802	32.6±0.222	33.22±0.2631	33.31±0.0975
**Spy0051**	30.06±0.7942	30.38±0.3733	30.61±1.0722	29.96±0.231	30.55±0.1084	30.31±0.1744
**Spy0052**	29.62±0.282	28.57±0.0352	30.34±1.2493	29.13±0.425	28.78±0.1341	29.85±0.358
**Spy0053**	29.99±0.2422	30.24±0.1577	31.02±0.7115	29.79±0.5337	30.3±0.7234	31.15±0.5504
**Spy0054**	32.6±0.0522	32.53±0.1091	33.87±0.1413	32.69±0.0008	33.02±0.1487	33.16±0.5591
**Spy0055**	31.76±0.2118	31.89±0.265	33.3±0.0345	31.52±0.0781	32.14±0.233	33.52±0.3434
**Spy0056**	33.61±0.085	33.49±0.136	34.39±0.0055	33.91±0.0698	34.12±0.1085	35.1±0.1568
**Spy0057**	30.52±0.757	30.42±0.2346	26.64±1.0485	29.71±0.9653	29.83±0.9088	30.86±0.6494
**Spy0058**	32.91±0.0169	32.69±0.094	32.84±0.1392	32.84±0.1071	33.1±0.2207	34.09±0.1569
**Spy0059**	31.77±0.4387	31.39±0.2475	32.26±0.0543	31.24±0.0493	31.88±0.1197	33.05±0.1501
**Spy0060**	32.56±0.1984	31.9±0.0364	32.96±0.0208	32.14±0.3366	32.72±0.0206	33.63±0.0821
**Spy0061**	33.57±0.292	33.24±0.3456	31.88±0.5807	33.14±0.2383	33.35±0.3456	34.01±0.2669
**Spy0062**	30.63±0.645	30.17±0.3466	31.14±0.0995	29.94±0.1866	30.39±0.0873	31.68±0.2494
**Spy0063**	33.22±0.2214	32.92±0.1071	34.28±0.0431	32.97±0.3034	33.52±0.0984	34.26±0.2365
**Spy0065**	29.23±0.0155	28.97±0.1796	27.9±0.3487	29.16±0.2949	28.55±0.1507	26.91±0.2642
**Spy0066**	28.75±1.2095	27.65±0.6188	30.15±0.4007	27.38±0.4002	28.31±0.339	29.94±0.0492
**Spy0067**	28.17±1.9507	27.87±2.0353	26.76±1.4097	27.38±1.0011	26.59±0.586	28.63±1.4716
**Spy0068**	30.94±0.5615	30.55±0.1899	31.32±0.4585	30.28±0.0539	30.85±0.0516	31.25±0.0801
**Spy0069**	30.98±0.244	31.45±0.2333	32.53±0.0499	31.2±0.3448	31.72±0.5805	32.8±0.467
**Spy0070**	30.96±0.268	31.61±0.0219	31.4±0.0858	31.4±0.0289	31.67±0.0152	30.9±0.3307
**Spy0071**	33.52±0.129	33.69±0.2033	34.5±0.09	33.43±0.1406	33.81±0.0654	34.21±0.2432
**Spy0077**	27.17±1.085	26.24±0.0281	26.38±0.3135	26.25±0.1583	27.25±0.5212	28.26±1.7144

**Spy0081**	29.57±0.1285	29.45±0.3632	31.48±0.148	29.55±0.1565	29.94±0.206	31.12±0.2251
**Spy0083**	30.75±0.044	31.38±0.5186	30.39±0.4953	31.11±0.215	31.26±0.3526	30.88±0.3091
**Spy0084**	30.73±0.2999	31.09±0.0663	30.76±0.2639	30.91±0.1836	31.14±0.0521	30.65±0.2129
**Spy0094**	30.32±0.0597	30.03±0.0766	26.92±0.6482	30.16±0.1247	29.98±0.2066	28.75±0.3963
**Spy0097**	29.24±0.155	30.18±0.3251	31.56±0.0056	29.95±0.4587	30.01±0.1312	30.74±0.1199
**Spy0104**	26.74±0.0608	25.92±0.8711	25.38±0.4048	26.29±0.3871	25.97±0.2435	25.61±0.0272
**Spy0105**	28.43±0.1772	28.44±0.824	26.1±0.2819	28.51±0.2126	27.89±0.2302	26.46±0.0943
**Spy0107**	27.18±1.2122	29.05±0.5556	31.01±0.2787	27.79±0.0775	28.43±0.2888	28.86±0.2456
**Spy0109**	25.08±0.5791	25.34±0.0698	26.36±0.6158	25.34±0.0561	25.31±0.2186	25.6±0.0092
**Spy0123**	25.71±0.4817	25.05±0.654	26.16±0.4967	25.72±0.0724	25.13±0.3133	26.24±0.7189
**Spy0134**	28.68±0.2711	29.01±0.1181	29.29±0.2182	28.06±0.8509	28.71±0.2865	26.97±0.5314
**Spy0136**	31.73±0.1527	32.16±0.1656	32.61±0.2465	32.34±0.2642	32.69±0.1576	32.02±0.1145
**Spy0137**	30.04±0.3262	29.24±0.2933	29.58±0.272	29.92±0.0151	30.49±0.0988	31.39±0.0897
**Spy0138**	28.89±0.7868	29.04±0.6885	26.24±0.1964	28.44±0.9792	28±1.002	27.29±1.527
**Spy0147**	31.57±0.2164	31.41±0.1663	28.43±0.4268	31.15±0.1384	31.06±0.0553	28.26±1.407
**Spy0163**	26.12±0.1406	24.84±0.52	25.92±0.8529	25.4±0.5223	24.89±0.0584	25.71±0.0973
**Spy0164**	26.64±0.5156	25.53±0.0755	25.74±0.0971	25.39±0.3548	25.05±0.0115	25.21±0.3521
**Spy0175**	27.06±0.6728	28.42±0.2412	30.3±0.1981	28.59±0.0635	29.08±0.167	30.86±0.0505
**Spy0178**	26.29±0.763	27.44±0.0949	25.87±0.5815	27.62±0.0609	27.61±0.1787	25.68±0.1205
**Spy0185**	32.99±0.0893	33.3±0.1391	35.42±0.0946	33±0.0177	33.46±0.0302	34.49±0.1331
**Spy0192**	29.95±0.0043	29.43±0.4456	28.01±0.3862	29±0.1449	29.05±0.3663	27.78±0.3084
**Spy0194**	31.21±0.2744	31.55±0.1677	29.91±0.4494	31.22±0.1934	31.1±0.1676	30.14±0.4919
**Spy0199**	26.34±0.1704	27.2±0.0646	29.4±0.0195	27.4±0.0385	27.05±1.089	28.8±0.2435
**Spy0202**	27.52±0.7714	29.6±1.166	29.39±0.355	29.71±0.0647	28.16±0.5374	28.99±0.1658
**Spy0203**	31.48±0.2827	31.46±0.1334	29.08±0.1118	31.11±0.135	30.74±0.148	29.61±0.4372
**Spy0209**	30.42±0.1908	29.91±0.0525	30.54±0.2779	29.5±0.1662	29.49±0.0174	29.05±0.1457
**Spy0220**	28.45±0.9605	29.37±0.2446	27.28±1.7491	29.02±0.2902	29.17±0.1007	26±0.1435
**Spy0222**	27.81±0.3803	27.16±0.4776	25.7±0.6349	27.66±0.326	27.55±0.0384	27.58±0.0126
**Spy0224**	28.48±0.6772	29.17±0.2095	31.8±0.4286	29.44±0.5218	29.92±0.211	32.05±0.2011
**Spy0227**	27.86±0.0247	27.72±0.2489	26.16±0.8457	27.31±0.1836	27.18±0.695	25.62±0.0995
**Spy0229**	26.05±0.5118	25.69±0.249	25.58±0.0483	25.18±0.3813	25.77±0.1986	25.74±0.3072
**Spy0230**	29.36±1.9463	28.21±1.7041	25.88±0.3795	27.28±0.5597	27.35±0.3741	28.54±0.9041
**Spy0231**	32.4±0.153	31.98±0.2769	33.07±1.1727	32.59±0.1481	32.86±0.3323	33.3±0.045
**Spy0232**	34.89±0.0925	35.03±0.0911	33.06±0.1175	34.43±0.0585	34.37±0.2386	33.13±0.4115
**Spy0233**	35.62±0.2942	35.67±0.4045	36.78±0.0419	35.14±0.0113	35.49±0.0585	34.16±0.0774
**Spy0242**	27.52±0.0216	27.47±0.0357	26.52±0.3691	27.36±0.4458	27.81±0.0801	25.56±0.0534
**Spy0243**	28.21±0.3016	28.43±0.3208	26.83±0.6983	28.83±0.2001	28.55±0.0149	28.2±0.1037
**Spy0246**	25.15±0.7433	25.26±0.3999	25.9±0.6419	25.89±0.2404	25.41±0.2261	25.98±0.0119
**Spy0249**	29.63±0.3598	29.51±0.1049	31.56±0.0249	29.19±0.0013	29.59±0.1257	30.43±0.0722
**Spy0261**	25.8±0.1258	25.29±0.0373	25.5±0.6259	25.16±0.3	25.54±0.4079	24.66±0.4638
**Spy0262**	26.03±0.8686	24.99±0.5211	27.74±0.1689	26.78±1.2735	26.48±0.9594	27.25±0.5229
**Spy0263**	26.68±0.0698	26.82±0.3777	25.29±0.6017	26.99±0.0774	26.58±0.3362	26.11±0.0905
**Spy0264**	25.91±0.5141	26.25±0.4688	25.59±0.1536	26.34±0.0066	26.27±0.294	25.61±0.266

**Spy0265**	26.43±0.6573	25.96±0.594	26.62±0.6234	25.7±0.3369	26.24±0.0957	25.84±0.1759
**Spy0266**	25.48±0.4429	25.12±0.2745	26.09±0.0171	25.31±0.0307	25.4±0.1052	26.08±0.1229
**Spy0269**	30.64±0.537	30.11±0.6726	26.76±1.1259	29.98±0.0845	29.08±0.771	25.65±0.0936
**Spy0270**	28.29±0.7264	28.67±0.1465	30.65±0.1227	30.42±0.0519	30.72±0.1053	31.76±0.0911
**Spy0276**	28.58±0.0637	28.61±0.4089	26.87±0.6824	28.71±0.0332	28.59±0.218	27.29±0.3578
**Spy0279**	26.53±0.3566	27.52±0.1948	28.39±0.5424	27.74±0.3089	27.82±0.2327	28.68±0.1809
**Spy0282**	29.56±0.4685	30.18±0.0259	30.11±0.0021	30.5±0.2855	30.65±0.0423	30.16±0.1525
**Spy0284**	25.33±0.1005	25.03±0.0153	25.15±0.4409	25.39±0.0422	24.72±0.3697	25.05±0.1074
**Spy0287**	29.5±0.3774	29.01±0.4576	26.29±0.1249	28.77±0.2914	28.27±0.0061	27.06±0.3119
**Spy0290**	29.28±0.2093	28.8±0.2162	26.94±0.2365	28.56±0.3272	28.14±0.2133	26.77±0.8259
**Spy0293**	30.9±0.4677	30.72±0.1373	30.22±0.6791	30.8±0.1938	30.64±0.1936	30.09±0.2003
**Spy0306**	25.64±0.2373	24.9±0.6571	25.53±0.2621	25.78±0.5638	25.66±0.2251	25.82±0.4712
**Spy0310**	26.28±0.7046	24.98±0.1473	25.63±0.6392	25.27±0.3899	25.37±0.2758	25.77±0.2089
**Spy0319**	32.62±0.3058	32.77±0.1003	31.95±0.1964	32.51±0.072	32.39±0.1036	32.07±0.4384
**Spy0327**	30.5±0.3021	30.17±0.1823	31.72±0.1135	30.37±0.1814	30.28±0.2282	29.62±0.2075
**Spy0328**	26.4±1.1186	28.6±0.079	27.75±0.3922	26.24±0.6952	27.43±1.051	28.19±0.3483
**Spy0330**	27.77±0.1326	27.42±0.4242	26.08±0.55	26.98±0.2477	26.37±0.1992	25.77±0.3656
**Spy0334**	26.47±0.1478	26.13±0.7839	25.84±0.2801	25.15±0.3314	26.28±0.1309	25.37±0.0541
**Spy0335**	25.84±0.2031	25.48±0.095	25.77±0.2652	25.77±0.3415	25.8±0.2126	25.47±0.2103
**Spy0337**	27.42±0.8656	28.82±0.2239	29.44±0.1161	29.46±0.3961	29.9±0.2186	29.2±0.3913
**Spy0345**	28.94±0.3482	28.57±0.2846	26.04±0.3365	28.32±0.0917	27.54±0.3829	26.25±0.0717
**Spy0349**	26.01±0.2942	26.05±0.1211	25.95±0.038	25.95±0.1823	26.12±0.0345	25.64±0.1795
**Spy0358**	25.12±0.3092	26.95±0.2711	25.78±0.4504	26.56±0.4191	26.7±0.2213	26.02±0.3947
**Spy0360**	25.53±0.0752	25.5±0.2318	26.21±0.3761	25.65±0.356	25.39±0.5857	25.43±0.0907
**Spy0362**	29.39±0.0767	28.62±0.1403	25.74±0.0308	28.17±0.0294	27.79±0.1837	25.46±0.1795
**Spy0363**	28.07±0.3885	28.4±0.0365	29.32±0.3516	28.32±0.0971	28.26±0.3743	27.97±0.3027
**Spy0365**	25.2±0.0899	27.4±0.0732	25.85±0.1021	25.28±0.9266	25.1±0.2297	25.85±0.1834
**Spy0368**	30.23±0.1242	31.23±0.0738	34.66±0.4674	32.22±0.1693	33.13±0.0986	35.09±0.323
**Spy0371**	27.75±0.7248	25.42±0.2399	25.76±0.15	26.04±0.3626	26.32±0.1266	25.74±0.5426
**Spy0374**	31.71±0.4147	31.64±0.3689	32.6±0.8054	31.56±0.2484	31.64±0.2954	32.79±0.1366
**Spy0375**	33.02±0.0985	32.91±0.0512	32.34±0.2649	33±0.1659	33.41±0.1022	33.83±0.07
**Spy0378**	28.25±0.5389	27.79±0.2484	26.6±0.1846	27.5±0.4832	27.26±0.5742	26.66±0.6539
**Spy0380**	32.36±0.6331	32.48±0.2606	33.87±0.5819	32.85±0.3711	33±0.209	33.97±0.3371
**Spy0382**	25.32±1.063	24.8±0.1953	26.11±0.1025	27.27±0.1874	25.9±0.9866	25.76±0.0477
**Spy0385**	29.64±0.1017	31.3±0.0682	29.29±0.3968	31.54±0.1591	31.73±0.0509	30.85±0.1211
**Spy0390**	28.99±0.3855	28.45±0.5556	26.23±0.1936	27.89±0.4232	27.49±0.3659	26.71±0.4149
**Spy0417**	30.95±0.2254	31.5±0.1536	33.14±0.1096	32.19±0.1914	32.27±0.0824	33.13±0.0269
**Spy0421**	28.84±0.0602	29.44±0.2622	26.84±0.4678	29.41±0.177	29.02±0.5685	27.12±0.7214
**Spy0422**	24.91±0.6351	25.06±0.3965	26.03±0.0982	26.08±0.2327	26.2±0.2598	26.72±0.4334
**Spy0423**	28.8±0.1252	29.73±0.3873	31.13±0.2028	29.85±0.2586	30.39±0.1054	30.21±0.173
**Spy0427**	30.47±0.5051	30.68±0.3551	27.26±0.3845	29.55±0.5305	29.11±0.1337	27.66±0.1976
**Spy0435**	28.03±0.4341	27.61±0.3594	26.25±0.5027	27.53±0.255	27.46±0.0689	26.73±0.722
**Spy0463**	28.23±0.329	28.38±0.1311	26.11±0.0504	28.64±0.1944	28.48±0.0735	27.92±0.0993

**Spy0470**	26.35±0.664	26.75±0.8533	25.54±0.3504	27.87±0.2634	26.84±0.3853	25.18±0.3104
**Spy0472**	29.98±0.4978	29.71±0.352	26.2±0.6257	29.72±0.0503	29.37±0.1492	28.42±0.3543
**Spy0480**	25.75±0.4547	25.4±0.043	25.52±0.0441	25.19±0.6018	25.43±0.1245	25.42±0.637
**Spy0484**	25.81±0.5234	25.08±0.1204	25.78±0.1542	26.07±0.6948	26.29±0.1949	24.92±0.0435
**Spy0487**	28.49±0.364	28.82±0.3574	27.15±0.2608	28.74±0.3512	28.2±0.2804	26.59±0.2895
**Spy0489**	26.06±1.1252	26.57±0.1899	25.72±0.4381	25.84±0.1785	25.65±0.0329	25.55±0.1352
**Spy0491**	25.59±0.2261	25.46±0.0958	25.52±0.4607	25.31±0.406	24.92±0.2364	25.56±0.4057
**Spy0493**	29.33±0.1415	29.35±0.2273	29.64±1.8122	30.34±0.9347	30.22±1.2256	29.88±0.9942
**Spy0495**	30.51±0.3266	29.85±0.3287	27.49±0.4067	29.88±0.4205	29.67±0.2658	28.17±0.2018
**Spy0498**	28.83±0.2501	27.92±0.1396	25.85±0.5405	27.67±0.0023	26.74±0.1036	26.15±0.1096
**Spy0504**	30.71±0.3484	31.1±0.032	31.79±0.2212	31.2±0.2509	31.48±0.1858	32.94±0.1962
**Spy0505**	26.42±0.1128	26.32±0.8441	25.93±0.194	26.44±0.0997	26.76±0.2653	25.77±0.004
**Spy0508**	36.78±0.0773	36.88±0.1125	34.82±0.2127	36.41±0.1051	36.24±0.1499	35.13±0.5522
**Spy0509**	34.37±0.0321	34.36±0.4596	32.52±0.0143	33.92±0.2924	33.91±0.2217	32.61±0.1762
**Spy0511**	27.2±0.1711	25.26±0.1314	25.48±0.1143	25.65±0.0491	25.87±0.5302	25.9±0.5227
**Spy0512**	27.42±0.2624	28.28±0.07	26.76±0.4525	28.92±0.5033	28.08±0.0949	27.37±0.2266
**Spy0514**	25.23±0.6066	25.82±0.7662	25.87±0.1954	25.9±0.2803	25.36±0.0838	25.49±0.0694
**Spy0516**	25.11±0.3297	24.73±0.0285	25.95±0.1587	25.7±0.6321	25.97±0.4539	25.39±0.1499
**Spy0530**	28.46±0.2444	28.48±0.5359	26.9±0.0977	28.89±0.2683	28.72±0.1082	26.65±0.6354
**Spy0531**	26.11±0.1703	26.7±0.3021	25.96±0.1492	27.56±0.1362	27.18±0.1662	26.91±0.0859
**Spy0533**	24.79±0.2781	25.64±0.1635	26.33±0.1956	26.3±0.3305	26.89±0.2338	25.65±0.0765
**Spy0538**	29.48±0.1732	29.39±0.0695	27.11±0.4831	29.2±0.107	28.95±0.0779	26.53±0.9251
**Spy0539**	28.48±0.0754	28.86±0.2376	25.73±0.4674	28.31±0.1365	28.01±0.0578	26.49±1.0652
**Spy0540**	25.76±0.5569	25.83±0.5938	25.77±0.0266	25.16±0.3595	24.85±0.0438	25.16±0.0784
**Spy0545**	25.45±0.0869	26.99±0.3295	26.09±0.5961	27.93±0.1452	27.2±0.1288	25.69±0.112
**Spy0546**	32.95±0.0079	33.11±0.2032	33.72±0.018	32.95±0.0983	33.27±0.0569	33.66±0.0458
**Spy0547**	27.63±0.0789	28.06±0.0063	27.12±0.578	28.14±0.2174	27.94±0.1088	28.29±0.4915
**Spy0551**	32.91±0.1034	32.58±0.2758	33.57±0.491	32.51±0.0151	32.72±0.1112	34.1±0.2973
**Spy0554**	24.95±0.397	25.12±0.5978	25.83±0.2509	25.53±0.7781	25.88±1.0315	26.29±0.3612
**Spy0556**	36.63±0.1252	37.2±0.0813	37.42±0.0006	36.4±0.1709	36.73±0.3415	37.42±0.3937
**Spy0574**	26.09±0.283	25.7±0.6165	25.54±0.0117	25.32±0.0711	25.81±0.1086	25.85±0.2804
**Spy0577**	25.17±0.1693	25.32±1.1502	26.71±1.234	25.42±0.4224	26.45±1.2178	25.27±1.0964
**Spy0579**	30.24±0.4566	30.92±0.0417	34.42±0.03	31.27±0.1393	31.88±0.2092	33.4±0.096
**Spy0580**	25.9±0.0523	26.22±0.1366	30.44±0.2849	26.28±0.0521	27.51±0.2517	29.74±0.1996
**Spy0581**	30.94±0.1073	31.56±0.1596	34.41±0.0244	31.54±0.0975	32.07±0.1392	33.41±0.1592
**Spy0582**	27.37±1.4851	27.69±1.0315	29.05±0.2318	26.4±0.4156	27.57±0.1216	28.92±0.8661
**Spy0584**	27.82±0.5114	27.45±0.4885	26.08±0.0298	28.13±0.1681	27.88±0.0762	26.46±0.0922
**Spy0587**	28.37±0.2379	27.66±0.381	25.68±0.5909	28.3±0.0984	28.44±0.0201	25.26±0.497
**Spy0588**	30.64±0.1558	30.54±0.1648	27.55±0.4919	30.86±0.0401	30.49±0.2248	28.89±0.2669
**Spy0597**	28.44±0.5549	28.32±0.6863	29.02±0.212	29.74±0.0121	29.57±0.162	30.42±0.4625
**Spy0598**	26.63±0.5531	28.5±0.1942	29.71±0.7468	28.4±0.4539	29.05±1.0654	29.15±0.8541
**Spy0600**	28.97±0.3953	29.32±0.1853	29.78±0.0945	29.56±0.0874	29.42±0.1122	29.32±0.0111
**Spy0602**	29.16±0.2444	29.46±0.0357	26.27±0.1248	29.39±0.0495	28.88±0.1972	27.24±0.3644

**Spy0606**	25.83±0.4286	25.39±0.158	25.68±0.5502	25.18±0.3016	25.54±0.0654	25.53±0.4362
**Spy0614**	28.15±0.2505	28.36±0.2641	25.87±0.1192	28.41±0.0023	28.65±0.0631	26.5±0.2476
**Spy0618**	28.25±0.0063	28.33±0.3843	25.55±0.0737	28.21±0.1333	27.66±0.2238	25.57±0.3593
**Spy0619**	29.88±0.1934	29.43±0.1406	29.62±1.2917	29.61±0.4827	29.49±0.3984	30.27±1.0662
**Spy0620**	29.92±0.0421	29.07±0.0566	29.79±0.5928	28.53±0.9158	28.93±0.8003	30.8±0.0212
**Spy0621**	29.41±0.0447	28.72±0.1767	30.87±0.4953	28.73±0.3512	29.59±0.6244	30.72±0.9113
**Spy0624**	26.45±0.0277	26.81±1.0804	26.57±0.611	26.3±0.1175	25.63±0.1366	26.37±0.0674
**Spy0626**	30.44±0.949	30.23±1.2455	30.13±0.5103	29.94±0.913	29.96±1.7213	30.6±0.8729
**Spy0627**	28.13±0.2629	28.85±0.124	30.85±0.4171	30.11±0.0255	30.47±0.2841	31.85±0.2913
**Spy0633**	32.03±0.1133	31.9±0.0654	33.81±0.1282	32.35±0.0139	32.88±0.0348	33.78±0.4748
**Spy0635**	31.31±1.3429	31.45±1.4911	29.95±0.0516	31.02±0.4808	30.69±0.0903	32.58±1.3506
**Spy0639**	27.94±0.1027	28.56±0.3187	26.56±0.3547	27.61±0.1597	26.32±0.3968	25.18±0.0508
**Spy0643**	25.27±0.0003	25.63±0.3432	25.76±0.4122	25.27±0.323	25.47±0.3557	25.64±0.1617
**Spy0644**	26.23±0.021	26.51±0.0143	25.7±0.2479	26.2±0.4496	25.1±0.2953	25.54±0.1861
**Spy0645**	26.15±0.8002	26.6±1.3949	26.31±0.6121	26.59±0.3523	25.66±0.2575	25.46±0.1569
**Spy0648**	31.96±0.0565	31.89±0.0769	32.24±0.1995	31.85±0.1992	32.43±0.0874	32.44±0.2038
**Spy0649**	26.97±0.1071	26.69±0.166	25.75±0.193	26.26±0.26	26.02±0.634	26.05±0.6904
**Spy0652**	25.88±0.5898	25.33±0.0197	25.98±0.5112	25.34±0.5076	25.78±0.3337	25.91±0.131
**Spy0655**	27.23±0.0423	26.9±0.3011	25.58±0.0023	26.14±0.1925	26.05±0.0768	25.61±0.4696
**Spy0659**	27.83±1.6549	28.45±2.0346	26.73±0.3291	26.28±0.3055	26.09±0.574	26.07±0.0849
**Spy0661**	28.93±0.3214	28.08±0.0652	25.86±0.5182	28.45±0.0749	28.38±0.2852	27.34±0.3379
**Spy0662**	27.63±0.4463	27.94±0.2144	28.49±0.0266	28.55±0.2668	29.42±0.3246	29.71±0.3485
**Spy0671**	26.46±1.11	26.1±1.3989	27.24±0.5673	26.5±0.6517	26.23±0.3522	28.61±0.9613
**Spy0674**	25.82±0.1519	25.42±0.7945	25.55±0.2042	25.52±0.2626	25.63±0.0987	25.44±0.0768
**Spy0677**	26.4±0.4674	26.1±1.0277	25.74±0.1826	27.57±0.3758	27.91±0.1192	26.17±0.0705
**Spy0685**	26.92±1.9777	28.41±0.9826	28.41±0.1899	27.58±1.4839	28.74±0.2689	28.95±0.1926
**Spy0686**	26.85±0.839	25.72±0.0239	25.69±0.6914	25.48±0.3959	24.58±0.3074	25.85±0.4435
**Spy0687**	28.37±0.2529	28.08±0.1802	25.93±0.4914	28.36±0.0676	27.82±0.2232	26.35±0.3695
**Spy0688**	26.1±0.4098	24.95±0.1206	26.62±0.4571	25.39±0.1833	25.72±0.1646	25.55±0.5118
**Spy0691**	30.45±0.0009	30.21±0.255	26.62±0.2771	29.69±0.2734	28.75±0.2898	27.18±0.9658
**Spy0692**	28.48±0.3739	25.57±0.0484	25.87±0.2424	25.64±0.0041	25.64±0.0822	24.95±0.0205
**Spy0694**	31.05±0.4823	31.15±0.235	30.59±0.372	31.38±0.1207	31.98±0.0741	31.04±0.5572
**Spy0696**	31.27±0.5909	31.79±0.2524	30.19±0.6108	32.02±0.3099	31.99±0.191	31.08±0.6218
**Spy0698**	28.69±0.5513	29.35±0.8935	29.91±0.3642	28.75±0.4227	29.16±0.0197	28.88±0.4186
**Spy0699**	30.7±0.2509	31.21±0.0949	29.56±0.4008	31.44±0.1756	31.85±0.0104	30.69±0.2492
**Spy0704**	26.19±0.2852	25.39±0.2158	26.09±0.3361	25.37±0.8248	25.43±0.2012	25.58±0.0083
**Spy0709**	27.2±1.9283	25.47±0.4712	26.3±0.0551	27.27±0.5729	26.17±0.8656	25.18±0.6948
**Spy0712**	25.82±0.0199	26.48±0.1034	26.01±0.4718	25.34±0.2808	25.81±0.188	26.08±0.2166
**Spy0713**	30.64±0.3626	30.49±0.2729	28.47±0.7092	30.09±0.0893	29.76±0.1341	29.59±0.0802
**Spy0714**	28.5±0.2917	28.14±0.4305	28.97±0.0772	28.12±0.0619	27.58±0.5762	26.69±0.1642
**Spy0715**	33.49±0.057	33.23±0.1717	31.76±0.3289	32.61±0.1452	32.43±0.2402	31.62±0.2479
**Spy0721**	27.11±0.3559	26.49±0.7086	25.96±0.8674	27.58±0.2736	26.23±1.2288	25.88±0.73
**Spy0725**	28.34±0.0389	28.24±0.0936	29.98±0.2605	28.5±0.1343	28.85±0.0682	30.01±0.0176

**Spy0728**	27.75±0.2754	28.19±0.9498	28.41±0.2246	29.29±0.0649	27.45±0.4592	26.27±0.1411
**Spy0731**	27.43±0.5656	26.95±0.3721	25.12±0.4439	26.25±0.6012	26.25±0.0682	25.9±0.5365
**Spy0732**	28.92±0.3477	29.16±0.0694	31.5±0.1026	29.21±0.2096	29.59±0.1763	31.49±0.0572
**Spy0734**	31.19±0.4507	31.35±0.0944	30.56±0.281	31.59±0.1495	31.4±0.0991	30.92±0.1267
**Spy0735**	30.74±0.4915	31.11±0.211	31.83±0.0292	31.18±0.1046	31.32±0.0764	30.43±0.1466
**Spy0736**	29.67±0.2754	29.32±0.205	26.1±0.018	28.82±0.3649	28.6±0.1684	26.42±0.8157
**Spy0737**	25.79±0.8805	25.64±0.493	25.83±0.9733	26.89±0.5142	26.71±0.6484	25.68±0.496
**Spy0739**	26.08±0.1614	25.09±0.0741	25.55±0.2564	25.47±0.0941	24.94±0.1554	26.14±0.2337
**Spy0747**	29.85±0.3412	29.66±0.1557	30.45±0.0259	30.32±0.228	30.68±0.0215	30.5±0.2683
**Spy0751**	30.43±0.3688	30.81±0.2953	28.24±0.5801	31.92±0.1437	31.87±0.0553	30.92±0.4531
**Spy0752**	32.14±0.4282	32.64±0.3533	30.74±0.7934	33.98±0.0738	33.76±0.1331	32.81±0.6091
**Spy0753**	30.17±0.4786	30.55±0.249	28.19±0.6811	31.89±0.0187	31.8±0.2046	31.29±0.22
**Spy0755**	32.96±0.2396	33.26±0.123	32.74±0.411	34.57±0.1034	34.52±0.0313	35.57±0.3072
**Spy0758**	30.37±0.2964	29.98±0.2646	26.28±0.1039	29.7±0.0841	29.24±0.1369	27.86±0.2348
**Spy0763**	31.45±0.0532	31.26±0.1987	28.73±0.4612	31.03±0.0847	30.75±0.1029	29.57±0.1333
**Spy0766**	25.21±0.3382	25.07±0.4697	26.03±0.0751	26.03±0.069	25.86±0.1028	24.98±0.1748
**Spy0767**	27.72±0.2076	28.08±0.0882	30.37±0.1335	28.48±0.1084	28.95±0.0694	30.73±0.354
**Spy0776**	28.98±0.3518	28.61±0.3011	25.77±0.5383	28.7±0.0825	28.66±0.0105	27.31±0.4271
**Spy0778**	25.68±0.1064	26±0.396	25.76±0.529	27.58±0.2916	26.83±0.1123	26.28±0.3596
**Spy0781**	25.21±0.1991	25.66±1.0411	27.48±0.5139	31.63±0.0373	31.14±0.1961	29.64±0.4971
**Spy0790**	25.29±0.6666	25.76±0.0133	29.64±0.107	30.21±0.2394	30.7±0.4678	32.42±0.4194
**Spy0792**	29.21±0.3242	27.69±0.2275	25.32±0.5553	30.72±0.1259	30.56±0.067	29.27±0.274
**Spy0793**	31.7±0.1391	32.32±0.2776	32.34±0.5252	32.27±0.0147	32.56±0.0579	31.64±0.1267
**Spy0794**	25.2±0.0589	24.51±0.0847	25.79±0.1823	24.89±0.336	24.93±0.1055	25.39±0.0463
**Spy0795**	32.99±0.0778	32.58±0.0387	33.84±0.1488	32.57±0.0006	32.77±0.0535	33.53±0.2393
**Spy0796**	35.06±0.0551	34.74±0.3062	35.79±0.1514	34.58±0.3078	34.79±0.6796	35.12±0.3755
**Spy0821**	29.92±0.1383	29.83±0.2492	28.38±0.0458	30.1±0.0676	29.98±0.0608	28.68±0.2078
**Spy0822**	29.48±0.1091	30.3±0.5433	26.58±0.3921	30.16±0.0704	29.48±0.4484	27.98±0.8821
**Spy0824**	28.77±0.6289	28.39±0.2875	28.31±0.3024	27.51±0.6604	28.36±0.2451	26.85±0.0785
**Spy0826**	27.3±0.0495	26.74±0.5074	27.44±0.1693	26.51±0.7343	27.61±0.2384	26.33±1.3467
**Spy0845**	28.99±0.3337	29.16±0.4409	27.93±0.265	29.23±0.3681	29.68±0.2819	29.46±0.3575
**Spy0847**	28.02±0.3166	28.32±0.2816	29.28±0.0441	28.5±0.0769	28.55±0.0805	29.05±0.2622
**Spy0848**	26.72±0.0313	26.61±0.283	27.5±1.279	27.13±0.0941	27.1±0.422	27.61±0.713
**Spy0851**	30.14±0.1462	30.81±0.061	28.55±0.2773	31.39±0.0864	31.02±0.1157	30.07±0.4828
**Spy0857**	25.53±0.909	25.06±0.5282	25.94±0.2382	25.01±0.3415	24.97±0.2927	25.92±0.0251
**Spy0858**	28.75±0.1837	28.44±0.1694	25.63±0.0056	28.55±0.2731	27.76±0.9698	26.34±0.0038
**Spy0863**	27.95±0.241	27.82±0.2876	25.83±0.4419	27.8±0.0822	27.84±0.0292	26.53±0.0964
**Spy0865**	25.14±0.5275	24.92±0.1987	25.91±0.462	26.32±0.5643	25.4±0.7917	25.89±0.1352
**Spy0867**	28.17±0.3706	28.51±0.0306	25.51±0.7123	29.61±0.0391	29.25±0.0782	28.17±0.6
**Spy0872**	31.8±0.0682	31.92±0.0338	29.88±0.1631	32.31±0.0276	31.98±0.0792	30.22±0.9143
**Spy0873**	33.46±0.2532	33.48±0.1721	32.04±0.5153	32.85±0.1904	32.56±0.227	31.32±0.4031
**Spy0876**	27.07±0.6729	27.02±0.1155	28.71±0.2911	27.51±0.4571	27.69±0.6664	29.49±0.5513
**Spy0878**	29.24±0.0302	29.19±0.4897	28.56±0.6728	30.43±0.3914	30.5±0.5074	29.59±0.0348

**Spy0885**	28.18±0.2591	27.99±0.312	28.42±0.0986	27.75±0.1128	27.84±0.2357	27.99±0.7355
**Spy0893**	28.07±0.1069	28.76±0.6163	25.04±0.694	29.64±0.0392	29.14±0.4887	27.85±0.2859
**Spy0907**	25.27±0.3728	24.91±0.5948	26.92±0.4373	25.32±0.0294	25.46±0.0644	27.65±0.1116
**Spy0910**	24.92±0.3384	25.54±0.469	25.8±0.3823	25.56±0.4055	25.17±0.716	25.85±0.1845
**Spy0915**	29.2±0.0207	28.23±0.7948	26.45±0.8324	27.97±0.0281	27.8±0.2744	26.86±0.1424
**Spy0919**	31.43±0.2981	31.27±0.0234	27.94±0.375	30.95±0.1836	30.81±0.1625	29.11±0.7589
**Spy0925**	27.14±0.7987	27.66±0.5291	25.88±0.1027	26.6±1.5186	27.42±0.6751	26.01±0.1119
**Spy0927**	27.01±0.0327	28.15±0.1656	25.62±0.3542	29.24±0.2123	28.96±0.1443	27.82±1.0181
**Spy0928**	26.78±0.5518	27.12±0.4752	25.79±0.4929	28.98±0.1818	29.02±0.0959	26.47±0.7179
**Spy0931**	24.76±0.5361	25.01±0.2369	25.83±0.2337	27.26±0.0896	27.16±0.0544	25.66±0.4835
**Spy0938**	30.67±0.0969	31.3±0.3092	28.39±0.2916	31.99±0.0927	31.14±0.11	29.92±0.0205
**Spy0941**	25.27±0.4777	25.19±0.021	25.66±0.6764	25.25±0.2962	25.26±0.8247	25.63±0.0293
**Spy0942**	27.26±0.4999	26.72±0.6504	30.51±0.1398	27.42±0.3462	28.55±0.1155	30.66±0.2118
**Spy0946**	29.95±1.6448	30.8±1.2876	33.69±0.095	31.25±1.6427	32.89±0.3745	33.97±0.4564
**Spy0949**	30.97±0.2948	30.86±0.0553	27.81±0.5004	30.87±0.0182	30.85±0.0337	29.44±0.4112
**Spy0950**	29.52±0.2178	29.22±0.4513	27.04±1.3963	29.47±0.1859	29.55±0.1166	29.59±0.5569
**Spy0951**	25.6±0.3178	25.78±0.057	26.08±0.0324	25.77±0.4093	25.45±0.4952	24.95±0.0798
**Spy0962**	27.57±0.5245	26.86±0.024	26.17±0.1517	25.26±0.0063	25.93±0.3191	25.64±0.6829
**Spy0971**	31.57±0.3293	31.16±0.7706	31.47±0.0012	32.13±0.0719	32.37±0.2522	31.52±0.1005
**Spy0972**	26.81±0.5639	26.26±0.2162	29.11±0.7086	27.14±0.4411	27.39±0.3482	28.79±0.1279
**Spy0973**	30.87±0.6691	30.85±0.9978	30.67±0.1402	31.79±0.1768	31.86±0.2096	31.52±0.084
**Spy0975**	24.84±0.9337	25.08±0.3866	26.37±0.4622	26.57±0.6394	27.24±0.8329	26.91±0.119
**Spy0977**	25.85±0.2225	25.64±0.4975	26.6±0.3026	24.95±0.4021	25.31±0.2274	25.39±0.1305
**Spy0986**	31.76±0.1917	31.27±0.5052	29.61±0.018	30.75±0.2763	30.56±0.2601	28.98±0.728
**Spy0988**	34.44±0.0172	34.59±0.011	32.99±0.0043	33.74±0.0774	33.55±0.0814	32.28±0.2785
**Spy0989**	32.75±0.0243	33.65±0.304	35.14±0.1878	32.86±0.0289	33.3±0.3424	33.54±0.0302
**Spy0991**	25.04±0.7294	25.29±0.4257	25.79±0.2449	25.45±0.2902	25.86±0.1882	25.54±0.0756
**Spy0992**	27.35±0.1735	27.77±0.243	25.48±0.3389	28.04±0.0273	27.3±0.7215	27.26±0.5733
**476_1104**[1]	26.51±1.5889	26.78±1.5654	25.46±0.3477	26.31±0.4828	24.82±0.0662	26.75±0.3985
**Spy1058**	27.48±0.2111	27.15±0.6507	25.5±0.0174	27.33±0.1536	27.24±0.1246	25.71±0.2365
**Spy1067**	27.21±0.5247	29.9±0.2362	32.21±0.1709	32.37±0.19	32.89±0.1757	33.27±0.1864
**Spy1073**	30.57±0.1138	30.65±0.1744	26.78±0.3925	30.52±0.0552	30.07±0.1688	28.4±0.2257
**Spy1075**	27.33±0.3711	25.89±0.231	24.82±0.4338	27.22±0.1078	26.42±0.4398	25.19±0.0268
**Spy1076**	26.01±0.3553	28.06±0.1506	30.9±0.7272	27.13±0.1723	28.77±0.0045	31.09±0.1763
**Spy1077**	29.48±0.3101	29.52±0.4553	29.64±0.215	29.76±0.0283	29.12±0.294	28.38±0.0524
**Spy1088**	28.45±0.4035	27.75±0.8318	25.51±0.5945	26.93±0.2937	26.17±0.7262	25.81±0.0306
**Spy1093**	25.16±0.6039	25.31±0.3237	25.49±0.4638	25.19±0.2247	25.43±0.0187	26.17±0.2196
**Spy1095**	28.34±0.0997	28.58±0.1931	26.08±0.0997	28.3±0.0771	28.13±0.0492	26.49±0.6415
**Spy1098**	26.27±0.7613	25.75±0.073	25.67±0.265	25.01±0.1422	24.56±0.1774	25.95±0.0698
**Spy1103**	29.17±0.1088	29.5±0.0287	26.25±0.9945	29.16±0.1301	29.14±0.0946	27.48±0.426
**Spy1106**	27.86±1.7912	28.12±1.4696	28.25±0.726	26.1±0.3153	25.43±0.4213	25.46±0.2716
**Spy1107**	27.52±0.0911	27.25±0.2844	25.64±0.5003	27.3±0.0782	26.32±0.3332	25.71±0.382
**Spy1108**	29.77±0.0744	29.3±0.2745	25.76±0.3382	29.92±0.0774	29.14±0.1529	27.68±0.1954

**Spy1116**	27.64±0.5663	26.9±0.2127	25.5±0.241	26.5±0.0674	26.01±0.6136	25.79±0.568
**Spy1117**	25.71±0.592	25.3±0.2403	25.83±0.3009	25.49±0.096	25.15±0.2492	25.99±0.052
**Spy1119**	33.99±0.0811	34.23±0.0245	32.9±0.071	33.84±0.0763	34.09±0.1443	32.74±0.0297
**Spy1120**	31.76±0.1679	31.79±0.0018	29.69±0.0589	31.61±0.0291	31.75±0.0963	30.34±0.331
**Spy1121**	29.74±0.607	30.91±0.2432	29.98±1.2517	31.08±0.6404	30.55±0.7227	29.1±1.6965
**Spy1122**	29.29±0.7437	28.06±0.0942	29.41±0.5015	27.68±0.6579	26.96±1.0751	25.79±0.7248
**Spy1123**	29.97±0.3291	29.51±0.1665	26.9±0.0268	30.1±0.0713	30.11±0.0623	28.35±0.1897
**Spy1124**	29.87±0.3198	29.95±0.0482	30.7±0.3057	29.75±0.0978	30.26±0.1383	31.66±0.1002
**Spy1132**	30.89±0.3649	30.9±0.0779	29.38±0.5464	30.77±0.1186	30.57±0.2033	28.54±0.8742
**Spy1133**	25.94±0.0039	26.33±0.1563	25.38±0.6261	26.01±0.7761	25.56±0.3166	25.8±0.2498
**Spy1136**	27.76±0.1823	28.08±0.0097	27.68±1.0152	26.99±0.9092	28.11±0.3148	27.66±0.0864
**Spy1139**	25.51±0.1367	24.97±0.0149	26.06±0.1389	26.13±0.356	24.9±0.0516	25.39±0.1743
**Spy1140**	25.81±0.0702	24.58±0.0142	25.74±0.4571	24.9±0.1018	24.73±0.2825	25.69±0.0234
**Spy1145**	29.1±0.4111	30.44±0.3991	31.35±0.701	31.49±0.3566	31.63±0.0751	32.67±0.077
**Spy1154**	29.89±0.3278	29.28±0.3649	26.65±0.8427	29.62±0.3269	29.24±0.4055	26.87±0.3849
**Spy1155**	30.07±0.2117	29.98±0.2893	25.98±0.8901	29.41±0.1524	28.83±0.3128	27.18±0.3564
**Spy1158**	28.03±0.0687	28.24±0.3406	30.45±0.1644	28.17±0.1168	28.47±0.2065	29.95±0.1363
**Spy1164**	33.04±0.1285	33.14±0.0014	33.88±0.3517	32.54±0.1457	32.62±0.2047	32.58±0.0108
**Spy1165**	27.88±0.8431	27.33±0.7127	25.6±0.0054	26.75±0.5035	26.44±0.487	25.75±0.1612
**Spy1214**	26.92±0.7276	26.5±0.3527	25.88±0.0806	25.25±0.4054	25.81±0.1847	26.14±0.2928
**Spy1223**	30.65±0.1689	31.31±0.2414	32.89±0.5023	30.77±0.1394	31.71±0.0178	32.33±0.8836
**Spy1226**	25.82±0.5169	24.94±0.8965	25.34±0.4808	25.27±0.2501	25.87±0.1197	24.98±0.2004
**Spy1235**	28.95±0.0584	30.28±0.1163	26.13±0.4645	32.07±0.1257	32.02±0.1458	30.76±0.349
**Spy1240**	30.92±0.0853	31.28±0.2324	28.92±0.5067	31.9±0.0203	31.8±0.0972	30.42±0.5758
**Spy1242**	32.21±0.3907	32.65±0.0263	31.36±1.5619	32.65±0.0675	32.84±0.0102	32.81±0.3936
**Spy1243**	30.71±0.1027	30.57±0.2822	25.94±0.8755	30.48±0.1411	30.19±0.0313	28.66±0.4309
**Spy1244**	31.57±0.325	30.96±0.1805	30.99±0.1156	30.91±0.0413	31.09±0.184	30.31±0.2119
**Spy1249**	31.82±0.3528	31.43±0.1294	30.29±0.0145	31.49±0.0166	31.23±0.0404	30.58±0.2136
**Spy1250**	29.21±0.0473	28.83±0.0222	26.62±0.3349	28.82±0.3741	28.65±0.2633	26.2±0.2026
**Spy1253**	27.29±0.2132	25.78±0.4444	26.18±0.3036	26.45±0.0251	25.94±0.0415	26.02±0.032
**Spy1255**	30.74±0.2458	30.76±0.2682	27.93±0.2314	31.08±0.1054	30.65±0.2194	29.34±0.2575
**Spy1257**	28.88±0.2267	29.05±0.3267	27.44±0.8991	29.4±0.0943	29.29±0.057	28.25±0.0895
**Spy1259**	27.38±0.2875	28.96±0.0871	29.89±1.4914	28.74±0.0707	29.3±0.2169	30.16±0.1635
**Spy1262**	25.53±0.011	25.25±0.1763	25.45±0.5369	25.35±0.1031	25.72±0.2107	25.64±0.161
**Spy1269**	26.44±0.2594	26.57±0.0391	25.87±0.3223	28±0.0838	26.69±0.8341	26.04±0.3856
**Spy1270**	25.64±0.2644	27.55±0.4307	31.12±0.3912	31.05±0.1388	31.85±0.5043	32.94±0.2529
**Spy1271**	25.79±0.0352	25.33±0.2238	27.22±0.768	29.9±0.2577	30.54±0.4175	29.74±0.4519
**Spy1273**	26.79±0.4544	31.1±0.2824	33.91±0.1703	33.89±0.174	34.56±0.1401	35.83±0.2025
**Spy1275**	27.55±0.6733	31.3±0.1331	33.56±0.4349	34.02±0.1408	34.62±0.4853	35.79±0.0128
**Spy1278**	28.33±0.4395	29.24±0.2742	26.08±0.445	29.66±0.1612	29.29±0.0338	26.8±0.5847
**Spy1292**	30.49±0.1308	30.36±0.1485	27.36±0.2262	30.28±0.1892	29.76±0.1672	28.19±0.813
**Spy1295**	27.3±0.3315	27.42±0.6497	29.24±0.1345	26.49±0.0076	26.25±0.6552	27.02±0.212
**Spy1296**	26.81±0.8088	24.82±0.1401	26.03±0.2943	25.41±0.0603	25.38±0.0406	25.63±0.1596

**Spy1298**	26.99±0.9557	27.07±1.1743	25.52±0.0394	26±0.781	25.29±0.3063	25.45±0.1209
**Spy1320**	27.11±0.1624	26.73±0.0261	26.05±0.8549	26.04±0.3838	25.92±0.1709	25.16±0.2946
**Spy1325**	31.6±0.2276	31.92±0.179	31.47±0.1218	32.23±0.1657	32.02±0.1724	32.07±0.2814
**Spy1329**	31.73±0.4353	31.3±0.342	29.65±0.2387	33.22±0.1963	32.65±0.1374	31.71±0.5087
**Spy1331**	30.42±0.3381	27.88±0.722	26.26±0.1284	29.02±0.123	28.72±0.1629	27.37±0.3231
**Spy1332**	25.85±0.3832	25.1±0.3248	26.11±0.3657	25.43±0.6472	25.59±0.0701	25.27±0.1664
**Spy1335**	25.21±0.3475	26.1±0.5298	25.77±0.3707	25.42±0.0509	26.29±0.238	26.68±0.5082
**Spy1336**	30.8±0.5733	30.7±0.0796	29.84±0.1716	30.99±0.0605	31.11±0.0207	29.93±0.1225
**Spy1338**	27.39±0.4899	26.22±0.1465	25.88±0.3577	26.7±0.4008	26.4±0.0912	26.12±0.1836
**Spy1340**	28.47±0.7461	29.03±1.0832	27.89±0.5596	28.97±0.6602	28.51±0.5859	28.11±0.2679
**Spy1341**	27.9±0.4482	27.76±0.2975	25.24±0.2483	27.14±0.4362	26.53±0.0381	25.85±0.1338
**Spy1344**	27.93±0.1922	27.67±0.002	25.79±0.3414	28.9±0.2058	29.08±0.2259	27.27±0.6042
**Spy1347**	27.11±0.1907	27.29±0.0311	26.87±0.323	28.1±0.2018	28.45±0.1689	27.1±0.2201
**Spy1350**	26.07±0.8851	27.74±0.1294	28.94±0.8873	27.77±0.8123	27.5±0.5665	27.24±1.1297
**Spy1352**	29.66±0.2772	28.22±0.3863	28.05±0.8339	27.34±0.057	27.63±0.3202	26.09±0.3599
**Spy1356**	32.16±0.1814	32.69±0.4073	34.05±0.1628	33.29±0.0009	33.68±0.0325	34.99±0.0179
**Spy1357**	28.33±0.1911	28.69±0.4087	25.97±0.1408	28.79±0.0748	28.66±0.4366	25.94±0.228
**Spy1358**	30.36±0.3868	30.57±0.3633	30.54±0.1299	30.86±0.2854	30.75±0.2046	29.47±0.2983
**Spy1360**	30.28±0.1571	30.7±0.0091	30.61±0.4367	30.83±0.1777	30.85±0.0479	30.73±0.0769
**Spy1364**	30.14±0.177	28.64±0.6697	26±0.0159	27.89±1.2912	25.94±0.4292	26.52±1.0566
**Spy1375**	32.96±0.1133	33.33±0.1431	34.65±0.0585	32.91±0.0742	33.51±0.1949	34.04±0.2173
**Spy1376**	25.3±0.0436	25.24±0.0929	28.37±0.457	28.41±0.4097	28.79±0.1029	29.86±0.3909
**Spy1382**	25.35±0.4192	24.92±0.7736	26.03±0.553	26±0.005	25.51±0.5748	25.46±0.1586
**Spy1383**	27.64±0.4756	28.59±0.8084	29.33±0.5851	28.06±0.2555	27.91±0.4188	29.34±0.1978
**Spy1384**	31.15±0.0191	31.19±0.0185	28.78±0.4983	30.92±0.0101	30.63±0.1151	28.84±0.0296
**Spy1385**	29.87±0.2791	30.28±0.1668	28.47±0.1277	30.09±0.2867	30.01±0.2122	28.8±0.4933
**Spy1387**	26.47±0.4904	27.69±0.0531	25.98±0.6205	28.66±0.1843	28.23±0.126	26.83±0.9858
**Spy1388**	26.53±0.1253	27.69±0.2574	25.77±0.5708	28.41±0.0424	28.32±0.4692	25.72±0.0148
**Spy1393**	25.29±0.6648	25.14±0.5325	26.2±0.6464	26.24±0.2448	24.89±0.5542	25.86±0.0483
**Spy1397**	25.3±0.4585	25.45±0.3974	25.84±0.1174	26.04±0.2369	26.18±1.3236	25.74±0.0074
**Spy1400**	25.05±0.351	25.05±0.0226	25.27±0.9009	27.03±0.408	27.19±0.0641	26.77±0.0678
**Spy1408**	27.28±0.2676	26.84±0.5542	26.58±1.211	27.49±0.2365	27.97±0.1096	27.02±0.2817
**Spy1409**	31.28±0.2517	31.08±0.0761	29.44±0.1134	31.26±0.1393	30.97±0.0399	30.19±0.3257
**Spy1411**	26.01±0.6592	25.75±0.5233	25.53±0.5	25.02±0.246	25.25±0.056	25.98±0.0669
**Spy1412**	30.02±0.1701	30.23±0.0917	26.39±1.318	29.55±0.0004	29.76±0.2078	28.25±0.4474
**Spy1469**	26.75±0.103	26.47±0.6048	25.8±0.3	26.94±0.2814	26.87±0.255	25.82±0.5104
**Spy1479**	29.54±0.3104	30.29±0.2873	30.68±0.1767	29.54±0.0691	30.12±0.3278	30.15±0.4049
**Spy1483**	30.73±0.1717	31.39±0.4069	30.83±0.2814	30.86±0.1003	31.24±0.0472	30.16±0.0106
**Spy1484**	29.5±0.7805	29.02±0.1496	26.44±0.1271	27.87±0.3792	28.21±0.1611	26.15±0.6684
**Spy1485**	28.84±0.4779	27.98±0.0128	25.96±0.782	28.24±0.0011	28.25±0.0113	27.1±0.0661
**Spy1486**	29.11±0.0325	28.5±0.6059	26.96±1.3393	28.55±0.2975	28.15±0.1745	27.74±0.7627
**Spy1487**	28.09±0.3534	27.72±0.2517	29.92±0.0787	27.53±0.5794	28.4±0.4384	28.61±0.8556
**Spy1488**	26±0.1659	26.99±1.1432	25.48±0.0394	25.96±0.4339	24.9±0.3197	26.13±0.2231

**Spy1489**	30.92±0.2775	30.82±0.6025	28.51±0.2529	30.86±0.2277	30.43±0.2621	29.65±0.3649
**Spy1490**	28.77±0.3407	28.48±0.4329	28.71±0.0295	28.48±0.0409	28.5±0.1883	28.37±0.0561
**Spy1491**	30.23±0.0287	30.06±0.2468	27.17±0.0449	29.7±0.0675	29.26±0.0115	27.79±0.138
**Spy1492**	29.37±0.0798	29.08±0.1642	26.28±0.6396	28.77±0.1276	28.63±0.0112	26.44±0.8533
**Spy1494**	25.68±0.1388	25.66±0.6264	25.82±0.1471	25.52±0.3714	24.74±0.0968	25.43±0.0513
**Spy1496**	28.68±0.0669	28.04±0.1778	30.05±0.1204	28.54±0.1689	28.72±0.0501	29.24±0.1421
**Spy1497**	30.59±0.062	30.35±0.0308	29.8±0.3476	29.56±0.0627	29.49±0.4634	29.13±0.5753
**Spy1498**	35.28±0.1864	35.72±0.1481	35.06±0.1152	34.95±0.0471	34.84±0.2783	34.42±0.1334
**Spy1499**	31.48±0.3866	31.88±0.0665	31.34±0.3813	31.4±0.0788	31.14±0.2377	30.17±0.4115
**Spy1506**	29.55±0.3353	28.98±0.283	26.8±0.3794	29.26±0.056	29.01±0.0663	27.35±0.7751
**Spy1507**	29.81±0.1842	29.31±0.2237	26.6±0.0444	29.15±0.0177	29.01±0.1727	27.81±0.1936
**Spy1512**	26.94±1.0202	26.93±1.1797	26.51±0.0575	25.78±0.1711	26.77±1.1068	27.22±0.597
**Spy1513**	30.29±0.2336	30.61±0.2001	28.97±0.2074	30.7±0.1162	30.74±0.112	29.59±0.1789
**Spy1514**	28.53±0.01	29.68±0.0804	29.54±0.0179	29.73±0.2551	29.64±0.2593	29.14±0.2246
**Spy1515**	25.94±0.4308	25.97±0.1244	25.7±0.274	25.56±0.3148	25.29±0.3905	25.63±0.1806
**Spy1532**	25.37±0.0581	25.76±0.0693	25.95±0.3654	25.56±0.3858	25.32±0.3245	25.18±0.2501
**Spy1534**	30.98±0.0349	30.52±0.1934	29.66±0.3793	30.3±0.1014	30.41±0.0123	30.19±0.1251
**Spy1538**	27.98±0.3785	28.25±0.1653	26.01±0.3237	28.14±0.2013	27.8±0.4411	27.23±0.128
**Spy1546**	29.64±0.1065	29.69±0.2946	28.74±0.2215	29.45±0.2975	29.62±0.2469	28.47±0.4128
**Spy1547**	31.56±0.2292	31.44±0.4235	30.73±0.3279	31.54±0.0592	31.29±0.0855	30.43±0.322
**Spy1553**	30.47±0.1461	30.42±0.0956	31.85±0.1319	30.7±0.0083	31.19±0.0141	32.02±0.2542
**Spy1554**	28.29±0.1343	28.64±0.1484	27.69±0.1831	27.84±0.0001	27.94±0.0871	27.32±0.3315
**Spy1555**	31.8±0.1363	31.45±0.3712	31.59±0.0656	31.95±0.0443	32.07±0.2995	32.43±0.4715
**Spy1559**	28.32±0.796	28.94±0.1182	29.53±0.1766	29.91±0.1761	29.73±0.2411	29.37±0.7131
**Spy1563**	26.11±0.2454	25.31±0.0385	26.33±0.5314	25.68±0.2911	25.24±0.0529	25.93±0.1835
**Spy1569**	30.08±0.0439	30.9±0.4234	28.78±0.3123	30.49±0.0374	30.38±0.3296	30.62±0.166
**Spy1571**	29.09±0.1833	29.52±0.4484	26.78±0.428	29.14±0.4285	28.74±0.4143	26.03±0.2154
**Spy1577**	29.02±0.5208	28.56±0.3637	26.35±0.5794	28.03±0.5045	27.83±0.3294	26.54±0.2306
**Spy1580**	31.25±0.4757	31.18±0.0598	31.96±0.2123	31.11±0.0221	31.17±0.1125	30.92±0.2655
**Spy1585**	27.66±0.5557	27.37±1.1787	26.16±0.3045	27.82±0.4853	27.6±0.638	27.04±0.0469
**Spy1587**	26.73±0.4764	27.1±0.0503	25.73±0.0164	28.79±0.1702	28.9±0.2463	26.54±1.2207
**Spy1591**	24.71±1.0632	26.27±0.1698	25.62±0.3426	26.8±0.0995	26.69±0.1605	25.83±0.3118
**Spy1595**	29.19±0.2969	28.87±0.0596	30.06±0.1565	29.6±0.2111	30.16±0.0016	30.38±0.292
**Spy1596**	32.19±0.1143	32.39±0.1841	34.13±0.0565	31.64±0.1999	32.16±0.3292	33.03±0.1641
**Spy1597**	26.97±1.0472	26.57±1.3112	25.96±0.0473	26.03±0.201	25.22±0.1514	25.57±0.1458
**Spy1599**	34.96±0.2726	35.19±0.0933	33.59±0.1669	34.7±0.0887	34.28±0.0153	33.29±0.1682
**Spy1600**	27.09±0.8051	25.47±0.3139	26.05±0.0907	25.49±0.1355	25.96±0.2056	25.74±0.0408
**Spy1601**	26.88±0.2153	26.99±0.4316	30.4±0.0637	26.75±0.1745	26.97±0.3747	28.72±0.1346
**Spy1602**	31±0.4431	30.72±0.2645	29.15±0.073	30.45±0.1453	30.16±0.269	29.2±0.3704
**Spy1603**	29.73±0.3386	29.7±0.2764	29.83±0.1339	29.91±0.0992	30.12±0.1126	29.81±0.329
**Spy1606**	29.19±0.006	28.4±0.1353	29.73±0.4189	28.64±0.3723	29.26±0.1853	30.59±0.1151
**Spy1607**	33.48±0.1331	33.8±0.0802	35.48±0.1036	32.95±0.0061	33.31±0.0803	33.91±0.2588
**Spy1610**	27.28±0.0015	25.91±0.2261	25.21±0.6129	26.36±0.1959	25.76±0.1886	25.55±0.469

**Spy1611**	27.12±0.4245	28.19±0.1134	26.12±0.2429	26.8±0.5412	27.02±0.328	25.53±0.0273
**Spy1612**	33.5±0.0573	33.27±0.0588	32.95±0.3624	32.86±0.1277	32.94±0.0349	32±0.2896
**Spy1614**	26.25±0.5536	26.76±0.842	25.76±0.3811	25.33±0.1222	24.84±0.0533	25.44±0.0877
**Spy1623**	24.9±0.3922	25.51±0.2597	25.83±0.1466	26.15±0.3607	25.88±0.7164	25.52±0.0509
**Spy1635**	25.58±0.1971	25.5±0.1885	26.93±0.262	27.07±0.1388	27.21±0.2434	26.39±0.2678
**Spy1646**	33.12±0.2425	33.29±0.0501	34.85±0.3036	33.17±0.3244	33.52±0.3375	34.68±0.3115
**Spy1647**	32.35±0.429	32.61±0.4804	33.8±0.7686	32.61±0.2388	33.12±0.3704	34.14±0.3064
**Spy1650**	28.83±0.5688	28.42±0.0356	25.88±0.9323	28.5±0.0452	27.87±0.6486	27.45±0.4557
**Spy1652**	26.38±0.9005	26.87±0.1609	26.29±0.3103	26.55±0.2142	25.56±0.1471	26.25±0.2071
**Spy1655**	28.3±0.1836	26.83±0.4099	25.79±0.5979	27.23±0.3114	25.87±0.5811	25.52±0.3312
**Spy1659**	27.62±0.2756	27.41±0.2338	26.01±0.6934	27.21±0.033	26.14±0.5653	25.46±0.0039
**Spy1660**	30.06±0.131	30±0.0869	29.53±0.025	30.13±0.1638	29.85±0.1276	29.04±0.219
**Spy1666**	32.38±0.3099	31.86±0.2215	33.76±0.0763	32.15±0.172	32.51±0.2272	33.52±0.3727
**Spy1669**	31.05±0.1761	31.55±0.0208	33.44±0.0146	31.85±0.2955	32.3±0.1816	33.36±0.14
**Spy1672**	27.44±0.3019	27.16±0.3647	26.59±0.3589	27.24±0.0327	26.76±0.0696	26.25±0.0516
**Spy1673**	31.79±0.1621	31.84±0.0909	31.47±0.2253	32.12±0.1608	32.15±0.0667	31.18±0.0123
**Spy1678**	26.23±1.1282	26.9±0.6555	25.82±0.0541	27.04±0.5317	27.29±1.002	26.48±0.4337
**Spy1682**	28.67±0.3343	28.34±0.1383	25.92±0.0832	28.58±0.228	28.43±0.2181	27.61±0.5906
**Spy1686**	25.85±0.2372	25.88±0.1184	26.2±0.1392	26.29±0.1004	25.33±0.0358	26.03±0.3495
**Spy1709**	28.96±0.2908	27.78±0.1818	30.13±0.4175	27.29±0.0745	27.31±0.3099	26.36±0.8461
**Spy1714**	28.93±0.2404	28.83±0.9095	28.57±1.3026	25.5±0.0877	25.25±0.0127	25.8±0.1138
**Spy1715**	28.81±0.641	28.33±0.5004	26.37±0.7959	25.91±0.0125	25.84±0.2166	25.88±0.1418
**Spy1719**	33.61±0.9607	34.01±0.86	33.86±0.4675	30.56±0.7493	29.35±1.0295	27.88±1.1964
**Spy1727**	26.34±1.3644	25.74±0.6182	25.64±0.3139	27.78±0.274	27.29±0.6543	25.49±0.4248
**Spy1728**	28.43±0.024	27.27±0.5451	25.2±0.0791	28.4±0.0642	28.7±0.2697	26.94±1.0603
**Spy1729**	27.87±0.3668	24.99±0.1527	25.89±0.2854	26.08±0.0604	26.18±0.7613	26.16±0.2746
**Spy1730**	25.97±0.1242	26.3±1.2369	27.4±0.5247	25.74±0.5862	27.27±1.5048	25.74±0.1508
**Spy1732**	30.64±0.2396	31.52±0.2151	29.79±0.6612	32.51±0.1863	33.06±0.1913	31.77±0.2617
**Spy1734**	25.52±0.1017	26.24±0.2195	27.62±0.2841	29.27±0.1591	29.81±0.1618	29.83±0.4236
**Spy1735**	28.69±2.2739	32.48±0.1208	33.12±0.3495	33.58±0.3183	33.63±0.0121	33.96±0.2505
**Spy1740**	25.9±0.9684	25.36±0.3027	27.57±0.0547	26.54±0.362	26.71±0.4814	27.89±0.1902
**Spy1741**	25.9±0.0588	28.02±0.3465	30.09±0.0893	26.89±0.1582	27.95±0.0179	29.61±0.1689
**Spy1742**	25.15±0.6773	25.64±0.2138	28.83±0.2711	26.14±0.0651	26.05±0.5813	28.38±0.0254
**Spy1752**	27.08±0.4734	26.56±0.2612	25.54±0.6268	25.66±0.9663	25.82±0.7985	25.05±0.1351
**Spy1761**	35.92±0.3394	36.35±0.4118	37.58±0.213	35.53±0.225	35.73±0.0497	36.46±0.1684
**Spy1762**	34.06±0.0685	34.49±0.1647	36.11±0.088	33.83±0.106	34.28±0.0589	34.95±0.2467
**Spy1763**	26.13±0.4334	25.26±0.1227	25.99±0.3349	25.72±0.0413	25.8±0.1957	25.72±0.2033
**Spy1768**	33.3±0.0975	34.21±0.1762	34.92±0.0231	35.29±0.1034	35.71±0.2046	35.71±0.4579
**Spy1769**	32.36±0.3463	32.41±0.1586	31.05±0.2492	33.9±0.0027	33.96±0.2279	32.47±0.505
**Spy1780**	32.52±0.0312	32.48±0.0662	31.74±0.0389	32.34±0.0135	32.66±0.1793	32.76±0.3605
**Spy1781**	34.17±0.2185	34.05±0.0728	32.8±0.4144	33.37±0.0117	33.14±0.2774	32.45±0.3704
**Spy1782**	31.59±0.5525	31.24±0.1559	28.36±0.4097	31.37±0.1765	30.95±0.2251	30.02±0.6104
**Spy1783**	24.9±0.3037	25.39±0.0536	25.95±0.1645	26.41±0.2141	25.41±0.3757	25.67±0.1455

**Spy1791**	25.45±0.2309	25.16±0.0736	27.3±0.5321	25.54±0.1344	25.19±0.4655	25.91±0.0119
**Spy1793**	27.93±0.1486	26.08±0.2444	25.97±0.299	25.65±0.1605	24.88±0.2909	25.44±0.0585
**Spy1796**	25.38±0.5738	25.7±0.8195	25.82±0.2239	25.29±0.0289	25.62±0.8328	25.92±0.0888
**Spy1797**	30.79±0.3826	30.74±0.1509	31.4±0.1767	30.96±0.066	30.82±0.004	30.27±0.128
**Spy1799**	30.59±0.1325	29.74±0.3771	27.86±0.2161	29.61±0.0836	29.35±0.1387	28.23±0.1504
**Spy1808**	32.43±0.2912	32.46±0.1526	30.64±0.386	32.52±0.2083	32.2±0.1898	31.26±0.5953
**Spy1813**	30.71±0.2021	30.61±0.0871	30.21±0.1345	30.7±0.1938	30.92±0.1676	29.77±0.0763
**Spy1814**	30.42±0.622	30.42±0.8613	29.44±0.1553	30.54±0.303	30.24±0.41	29.2±0.2134
**Spy1816**	27.52±0.1898	27.08±0.6153	27.1±0.0676	27.22±0.2568	27.56±0.3434	29.03±0.0405
**Spy1831**	31.86±0.7855	31.37±0.7245	33.26±0.3238	31.72±0.1209	32.01±0.0434	33.71±0.1343
**Spy1836**	30.65±0.3943	30.56±0.2112	31.93±0.0919	31.21±0.0297	31.51±0.0788	32.57±0.2138
**Spy1838**	26.71±1.0964	26.05±0.0574	25.8±0.4549	25.89±0.4845	26.19±0.8779	25.36±0.1542
**Spy1840**	29.49±0.7688	30.54±0.2694	28.5±0.1467	29.02±0.074	29.03±0.0751	28.09±0.5062
**Spy1842**	28.12±0.3104	27.19±0.2057	25.78±0.1721	25.61±0.068	25.17±0.2023	25.67±0.5329
**Spy1848**	26.81±0.6184	27.07±0.9042	25.8±0.0448	25.84±0.5142	25.67±0.1133	25.54±0.0197
**Spy1857**	32.86±0.2555	32.81±0.0632	30.93±0.0695	31.93±0.1268	31.65±0.1272	30.71±0.3648
**Spy1858**	28.31±0.217	27.69±0.365	25.73±0.2917	27.52±0.3342	27.19±0.1464	25.48±0.4332
**Spy1861**	31.87±0.2697	31.61±0.1	29.97±0.1154	31.01±0.0694	30.61±0.0261	29.51±0.4119
**Spy1865**	27.79±0.1724	29.28±0.1455	27.23±0.3568	29.38±0.0823	30.14±0.0729	29.74±0.0134
**Spy1866**	27.38±0.0804	27.03±0.2131	25.67±0.121	27.03±0.1944	27.24±0.0678	26.13±0.1689

[1] Protein not annotated in M5005 genome, annotation refers to *S. pyogenes* M1 strain 476
