# Supplementary material for: Global Analysis and Comparison of the Transcriptomes and Proteomes of Group A Streptococcus Biofilms
Source: mSystems. 2016 Dec 6;1(6):e00149-16. doi: 10.1128/mSystems.00149-16 (PMC5141267; doi:10.1128/mSystems.00149-16)
Supplement: Table S3 [file sys006162066st10.pdf]

Table S3. Real-time RT-PCR primers used in this study

Primer Name	Primer Sequence
arcC M1 RT L	5’-ATTCTGGCGTTGTTGTGGT-3’
arcC M1 RT R	5’-AGCTTCCACACCAGTCAGG-3’
emm1 M1 RT L	5’-ACTCCAGCTGTTGCCATAACAG-3’
emm1 M1 RT R	5’-GAGACAGTTACCATCAACAGGTGAA-3’
gyrA M1 RT L	5’-GCTCCATCACTTCCTGATTATTGAC-3’
gyrA M1 RT R	5’-CGACTTGTCTGAACGCCAAAG-3’
speB M1 RT L	5’-GGTAAAGTAGGCGGACATGCC-3’
speB M1 RT R	5’-CACCCCAACCCCAGTTAACA-3’
