# Supplementary material for: Global Analysis and Comparison of the Transcriptomes and Proteomes of Group A Streptococcus Biofilms
Source: mSystems. 2016 Dec 6;1(6):e00149-16. doi: 10.1128/mSystems.00149-16 (PMC5141267; doi:10.1128/mSystems.00149-16)
Supplement: Figure S2 [file sys006162066sf3.pdf]

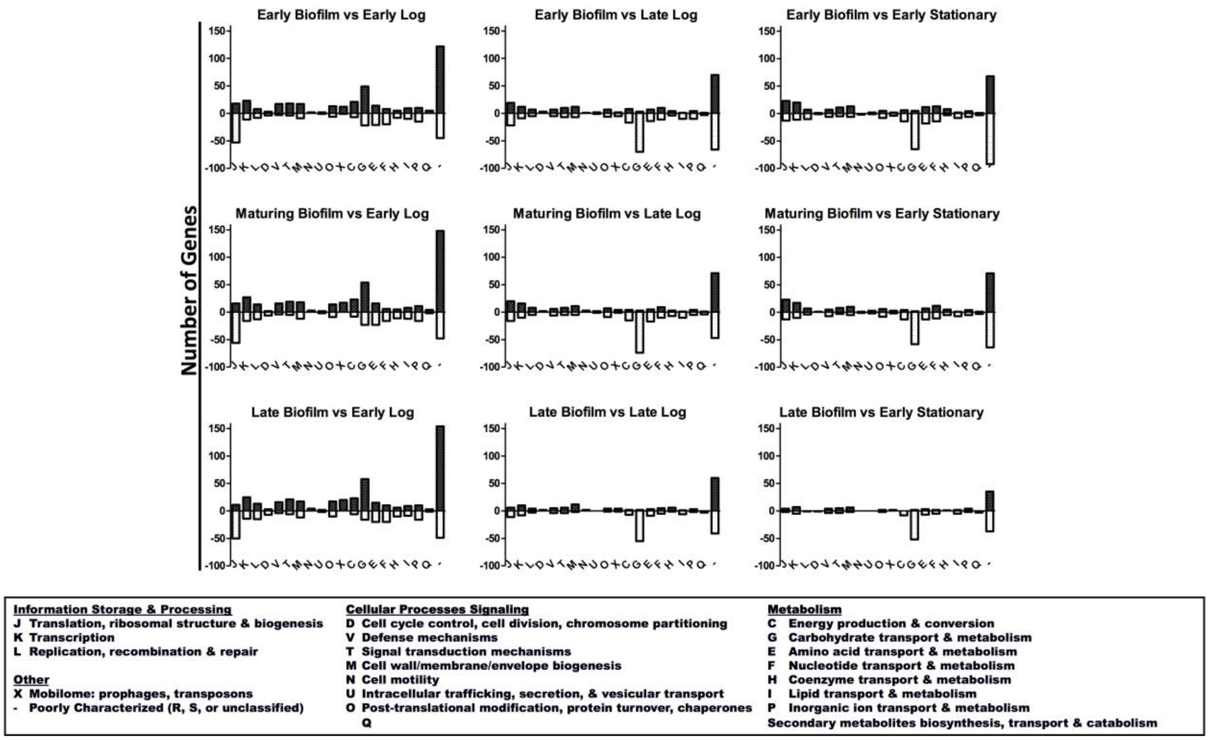

Figure S2. Differential regulation of biofilm versus planktonic cellular proteome according to COG classifications. The number of cellular proteins in each COG differentially regulated at each biofilm versus planktonic time point are shown. Dark bars indicate the number of cellular proteins in the COG up-regulated and light bars indicate the number of cellular proteins down-regulated. COGs were analyzed with the R-package BOG (21) to identify COGs with a statistically greater than expected number of cellular proteins showing differential expression. *=adj. p value < 0.05 according to the Mann-Whitney Rank Sum test. The “Poorly Characterized” group includes COG classifications R (general function prediction only) and S (unknown function) in addition to unclassified proteins.
