# Supplementary material for: Global Analysis and Comparison of the Transcriptomes and Proteomes of Group A Streptococcus Biofilms
Source: mSystems. 2016 Dec 6;1(6):e00149-16. doi: 10.1128/mSystems.00149-16 (PMC5141267; doi:10.1128/mSystems.00149-16)
Supplement: Figure S3 [file sys006162066sf4.pdf]

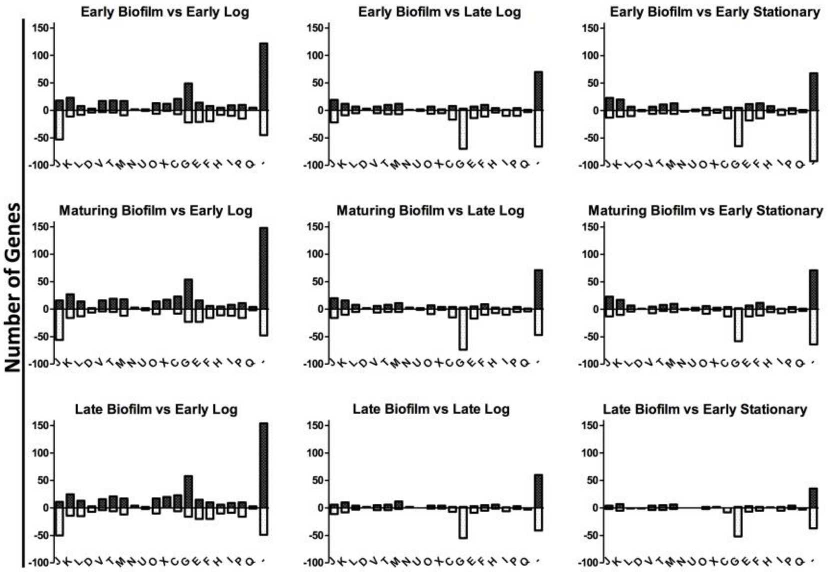

Information Storage & Processing
J Translation, ribosomal structure & biogenesis
K Transcription
L Replication, recombination & repair

Other
X Mobilome: prophages, transposons
- Poorly Characterized (R, S, or unclassified)

Cellular Processes Signaling
D Cell cycle control, cell division, chromosome partitioning
V Defense mechanisms
T Signal transduction mechanisms
M Cell wall/membrane/envelope biogenesis
N Cell motility
U Intracellular trafficking, secretion, & vesicular transport
O Post-translational modification, protein turnover, chaperones
Q

Metabolism
C Energy production & conversion
G Carbohydrate transport & metabolism
E Amino acid transport & metabolism
F Nucleotide transport & metabolism
H Coenzyme transport & metabolism
I Lipid transport & metabolism
P Inorganic ion transport & metabolism
Secondary metabolites biosynthesis, transport & catabolism

Figure S3. Differential regulation of biofilm versus planktonic cell wall proteome according to COG classifications. The number of cell wall proteins in each COG differentially regulated at each biofilm versus planktonic time point are shown. Dark bars indicate the number of cell wall proteins in the COG up-regulated and light bars indicate the number of cell wall proteins down-regulated. COGs were analyzed with the R-package BOG (21) to identify COGs with a statistically greater than expected number of cell wall proteins showing differential expression. *=adj. p value < 0.05 according to the Mann-Whitney Rank Sum test. The “Poorly Characterized” group includes COG classifications R (general function prediction only) and S (unknown function) in addition to unclassified proteins.
