# Supplementary material for: Global Analysis and Comparison of the Transcriptomes and Proteomes of Group A Streptococcus Biofilms
Source: mSystems. 2016 Dec 6;1(6):e00149-16. doi: 10.1128/mSystems.00149-16 (PMC5141267; doi:10.1128/mSystems.00149-16)
Supplement: Figure S4 [file sys006162066sf5.pdf]

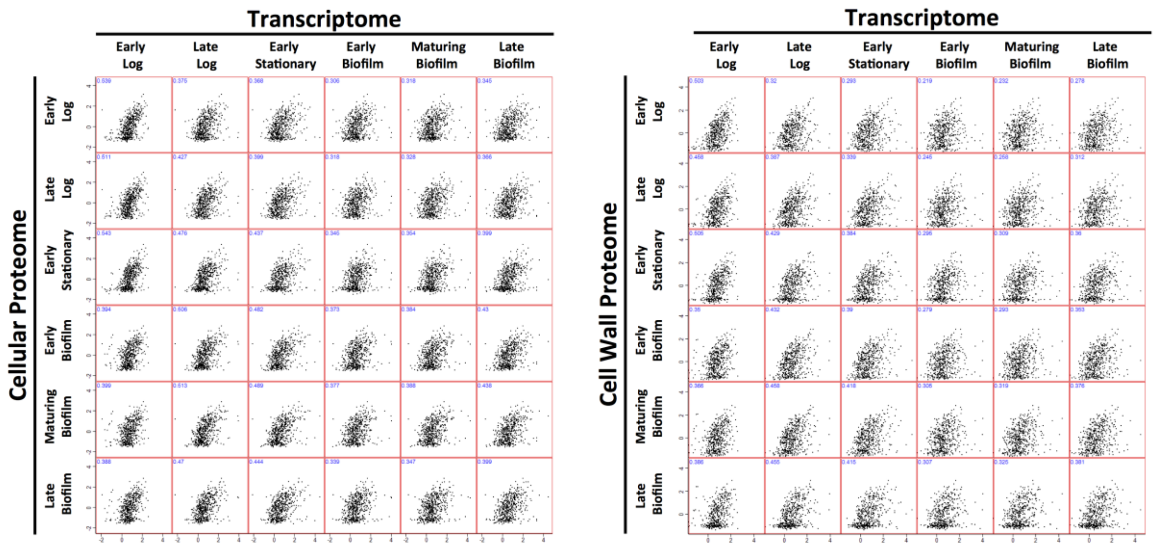

Figure S4. Multiple scatter plots showing correlation between planktonic and biofilm time points. Scatter plots show the z-scored proteome expression values plotted against the transcriptome expression values for all possible time point combinations. All genes with corresponding proteins identified in either the cellular proteome (A), or the cell wall proteome (B) are shown. The numbers in the upper left-hand of each box indicate Pearson correlation coefficients.
