# Supplementary material for: Global Analysis and Comparison of the Transcriptomes and Proteomes of Group A Streptococcus Biofilms
Source: mSystems. 2016 Dec 6;1(6):e00149-16. doi: 10.1128/mSystems.00149-16 (PMC5141267; doi:10.1128/mSystems.00149-16)
Supplement: Figure S5 [file sys006162066sf6.pdf]

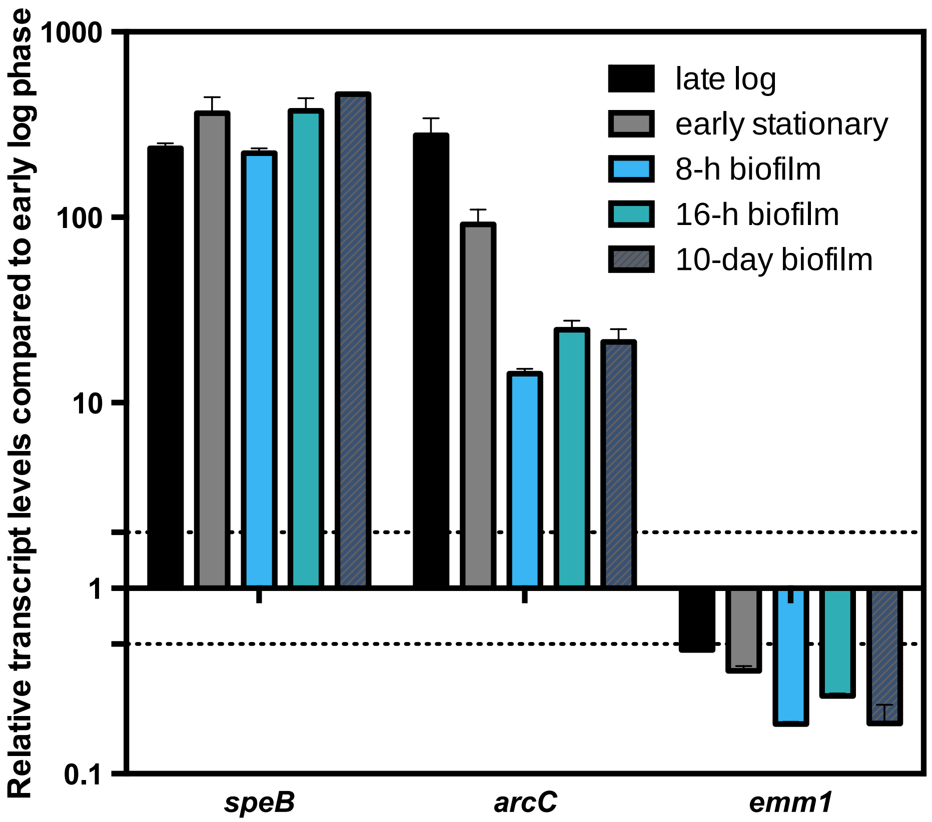

Figure S5. Real-time RT-PCR measurement of gene expression. Expression of the *speB*, *arcC* and *emm1* gene transcriptions were measured in total cellular RNA extracts from early log planktonic, late log planktonic, early stationary planktonic, early biofilm (8-h), maturing biofilm (16-h), or late biofilm (10-day) cultures. Transcript levels are represented as the ratio of expression at a given time point versus expression in early log phase. Error bars indicate standard error.
