# Supplementary material for: Global Analysis and Comparison of the Transcriptomes and Proteomes of Group A Streptococcus Biofilms
Source: mSystems. 2016 Dec 6;1(6):e00149-16. doi: 10.1128/mSystems.00149-16 (PMC5141267; doi:10.1128/mSystems.00149-16)
Supplement: Figure S6 [file sys006162066sf7.pdf]

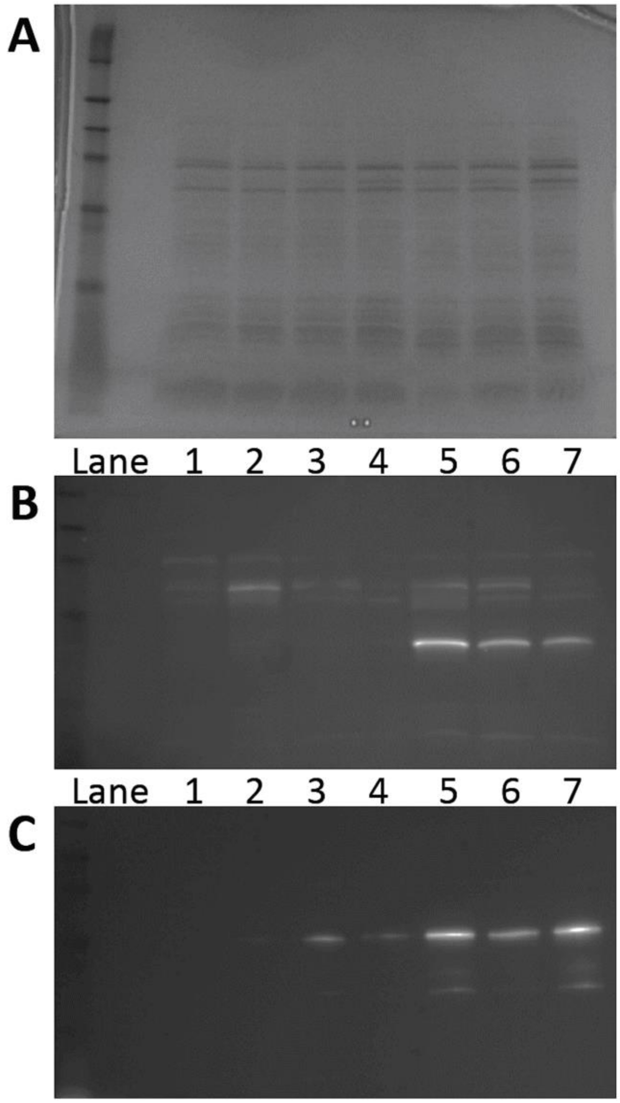

Figure S6. Western blotting of total cellular protein extracts. 1 µg of total cellular protein extracted from either an early log planktonic (lane 1), late log planktonic (lane 2), early stationary planktonic (lane 3), late stationary planktonic (lane 4), early (8-h) biofilm (lane 5), maturing (16-h) biofilm (lane 6), or late (10-day) biofilm (lane 7) culture was separated by SDS-PAGE. Gels were either stained with coomassie blue as a control (A), or transferred to a PVDF membrane and probed with anti-SpeB (B) or anti-ArcC (C) antibody.
