# Supplementary material for: Global Analysis and Comparison of the Transcriptomes and Proteomes of Group A Streptococcus Biofilms
Source: mSystems. 2016 Dec 6;1(6):e00149-16. doi: 10.1128/mSystems.00149-16 (PMC5141267; doi:10.1128/mSystems.00149-16)
Supplement: Text S1 [file sys006162066s1.pdf]

Text S1. Supplemental Materials and Methods

Quantification of expression by real-time RT-PCR.

For RNA-seq data validation, real-time RT-PCR analysis was carried out using primers in Table S3 as follows: 25 ng of DNase I-treated total RNAs were added to SYBR green master mix (Applied Biosystems) along with 250 nM of each gene-specific real-time primer (20-nM) stock using the one-step protocol on a LightCycler 480 instrument (Roche). Each sample was assessed in triplicate and processed on a Lightcycler 480 real-time PCR system (Roche). Transcript levels of *speB*, *arcC* and *emm1* in the different growth conditions (late log phase, early stationary, 8-h biofilm, 16-h biofilm and 10-day biofilm) were compared to those of found in GAS 5448 grown at early log phase relative to the level of expression of *gyrA* transcript and data is represented as the ratio of the mean with standard error.

Validation of protein expression by western blot.

For LC-MS/MS data validation, western blot analysis was carried out as follows: 1 µg of total cellular protein extracted from different growth conditions (early log phase, late log phase, early stationary, late stationary, 8-h biofilm, 16-h biofilm and 10-day biofilm) was separated by SDS-PAGE using 10-20% Tris-HCl resolving gels (Bio-Rad). Proteins were then either stained with coomassie blue as a control, or transferred to a polyvinylidene difluoride (PVDF) membrane for Western blotting. Bacterial proteins that were transferred to a PVDF membrane were blocked in 5% nonfat dry milk overnight at 4°C. The membranes were treated with a 1:500 dilution of either rabbit anti-SpeB antibody (obtained from Matak Kolb) or mouse anti-ArcC antibody (produced as described below), followed by a 1:2000 dilution of the appropriate secondary antibody (either Goat anti-rabbit or Goat anti-mouse IgG horseradish peroxidase (HRP)-conjugated antibody). Bound secondary antibodies were detected using

SuperSignal chemiluminescent substrate (Pierce) as described by the manufacturer.

Production of anti-ArcC mouse IgG.

Recombinant ArcC protein was produced by cloning the *S. pyogenes* 5448 *arcC* using the PCR primers BsaI-5'-arcC (ACTCTAGGTCTCACTCCATGACGAAACAAAAAATCGTAGTCG) and BsaI-3'-arcC (ATGGTAGGTCTCATATCTTACCCTGCGATAATTTGTGTTCC). The resulting PCR product was digested with BsaI and ligated into the pASK-IBA14 expression vector (IBA) to make pJAF102. pJAF102 was transformed into *E. coli* BL21/DE3 and expression of recombinant ArcC was induced using anhydrotetracycline. The recombinant ArcC was purified from *E. coli* cell lysates using Strep-Tactin spin columns (IBA). The purified ArcC protein was mixed with alum and injected into CD1 mice. The mice received a second booster shot of ArcC protein. Four weeks after injection, serum containing anti-ArcC IgG was harvested from the mice.

The mouse anti-ArcC IgG was tested against both recombinant ArcC and *S. pyogenes* whole cell lysate in order to confirm its specificity for ArcC. All mice were housed in the University of Maryland, Baltimore Dental School Animal Biosafety Level 2 facility. Animal use was approved by the University of Maryland, Baltimore Institutional Animal Care and Use Committee.
